# Cooperative assembly of H-bonded rosettes inside a porphyrin nanoring†

**DOI:** 10.1039/d0sc06097f

**Published:** 2020-12-08

**Authors:** Petr Motloch, Pernille S. Bols, Harry L. Anderson, Christopher A. Hunter

**Affiliations:** Department of Chemistry, University of Cambridge Lensfield Road Cambridge CB2 1EW UK herchelsmith.orgchem@ch.cam.ac.uk; Department of Chemistry, Chemistry Research Laboratory, Oxford University Oxford OX1 3TA UK harry.anderson@chem.ox.ac.uk

## Abstract

The melamine·barbiturate H-bonded rosette motif is of comparable dimensions and symmetry to the cavity of a butadiyne-linked 6-porphyrin nanoring. Functionalisation of each of the barbiturate components and the pyrimidine components of a H-bonded rosette with a pyridine ligand leads to a self-assembled hexapyridine ligand, which binds cooperatively to the zinc porphyrin nanoring. UV-vis-NIR and ^1^H NMR experiments show that the 7-component assembly forms at concentrations at which neither the H-bonding interactions nor the zinc porphyrin–pyridine interactions are formed in the absence of one of the three components. The mean effective molarities of these rosette complexes are around 200 mM in chloroform at 298 K.

## Abstract 2

Mixing barbiturates and pyrimidines equipped with pyridine ligands to leads to self-assembly of a hexadentate rosette ligand, which is complementary to a hexameric zinc porphyrin macrocycle.

## Graphical abstract

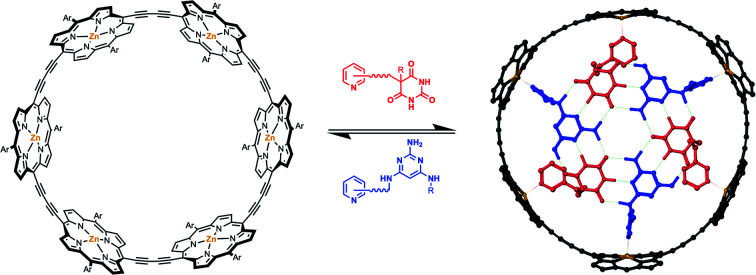

## Introduction

**Fig. 1:**
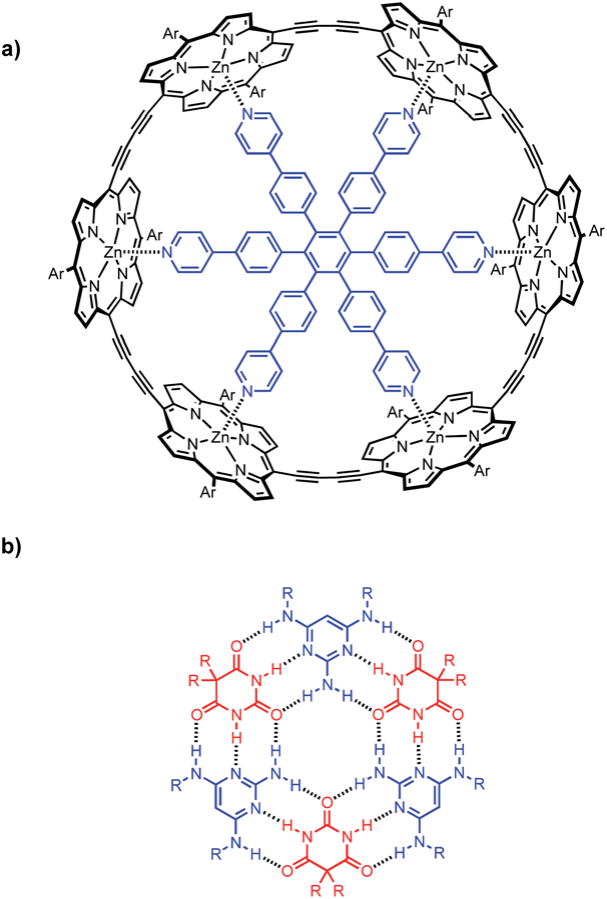
(a) Complex of c-P6 (black) with hexapyridine ligand T (blue). (b) H-bonded rosette formed from pyrimidines (blue) and barbiturates (red). Ar is 3,5-bis(*t*-butyl)phenyl, and R is a substituent.

Multivalency is a characteristic feature of many biological assembly processes.^1^ The product of multiple binding interactions is greater than the sum of individual binding contributions because of chelate cooperativity.^2^ The key parameter for quantifying chelate cooperativity in a multivalent system is effective molarity (EM). Effective molarities for cooperative interactions in multivalent supramolecular systems are generally of the order 100 mM, but a small number of remarkable systems with a value of EM greater than 100 M have been reported.^3^ One of the highest values was found for complex c-P6·T shown in Fig. 1a.^4^ Using a series of ligands with two – six pyridine binding sites, stepwise EMs were determined for each binding interaction.^5^ The value of EM for the first intramolecular binding interaction is 100 mM, but the four subsequent intramolecular binding interactions have values of EM of around 1000 M. Another supramolecular system that shows a relatively high degree of chelate cooperativity is the H-bonded rosette motif shown in Fig. 1b first reported by Whitesides,^6^ which has an EM of 2 M for macrocyclisation.^7^ Here we combine these two motifs to assemble a multivalent hexapyridine rosette inside the porphyrin hexamer and quantify the associated cooperativity.

**Fig. 2:**
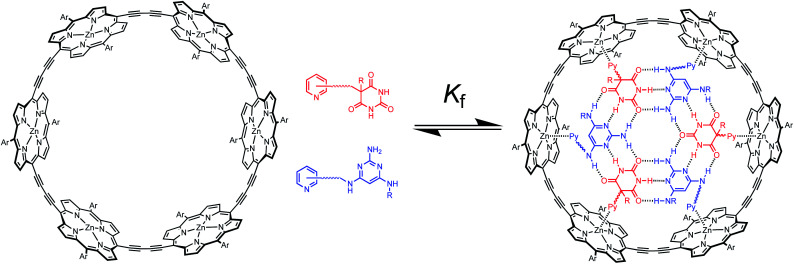
Formation of a rosette inside c-P6 using pyridine ligands equipped with either barbiturate (red) or pyrimidine (blue). Ar is 3,5-bis(*t*-butyl)phenyl, R is a substituent, and Py represents a pyridine unit.

Fig. 2 illustrates the approach. Functionalisation of pyridine ligands with the H-bonded rosette components should allow self-assembly of a hexapyridine rosette, which is complementary to c-P6.

**Fig. 3:**
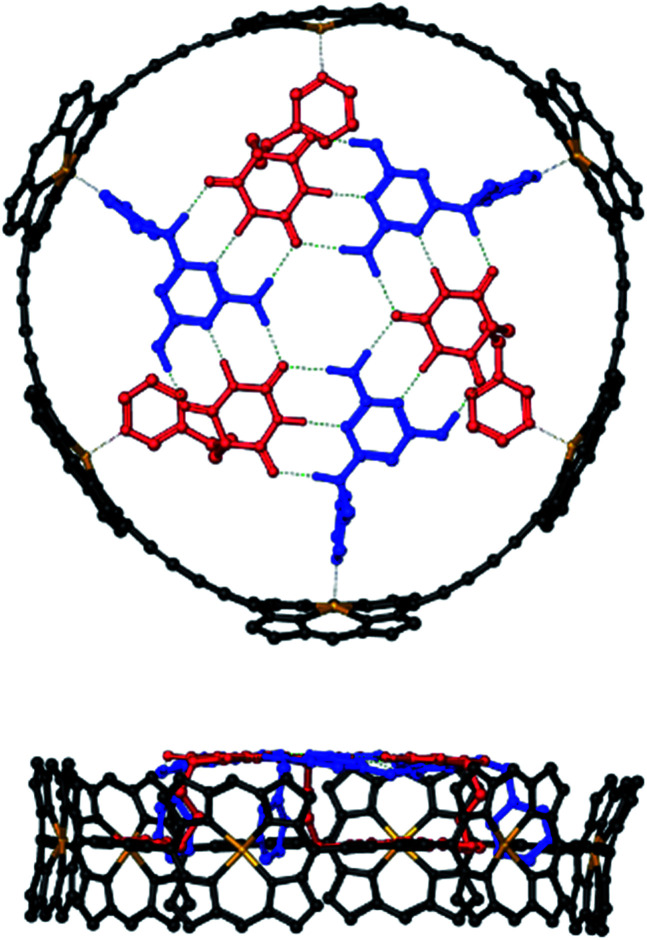
Top and side views of a PM6-optimised structure of a hexapyridine H-bonded rosettes bound inside c-P6 (see ESI Section 5† for details). Alkyl groups on the ligands and the 3,5-bis(*t*-butyl)phenyl groups on c-P6 were replaced by hydrogen atoms in the calculations. c-P6 is shown in black, zinc in yellow, barbiturates in red, pyrimidines in blue, and H-bonds in green. Hydrogen atoms that do not contribute to H-bonding are not shown for clarity. The ligands correspond to mB3 and mA1 in Schemes 1 and 2.

Molecular modelling was used to examine the size complementarity of the rosette and the porphyrin nanoring. Fig. 3 shows a model of a possible structure for the 1 : 3 : 3 complex in Fig. 2. The diameter of the rosette is only slightly smaller than that of the porphyrin nanoring, so there is not enough space to fit a pyridine ligand between the outer rim of the rosette and inner rim of c-P6. The pyridine substituents must therefore be attached to the rosette components in such a way that they project from one face of the rosette.

## Results and discussion

The model in Fig. 3 shows that a short alkyl linker should allow the pyridine ligands to sit in the plane of the zinc centres of the porphyrin nanoring with the H-bonded rosette perched above this plane. A number of different pyridine derivatives of barbiturate and pyrimidine, which have different length and geometry alkyl linkers, were therefore explored to maximise the probability of finding a complementary self-assembled system. The nomenclature used to describe different linkers below is *m* or *p* to indicate the location on the pyridine ring and a number to indicate the number of CH_2_ groups.

**Scheme 1:**
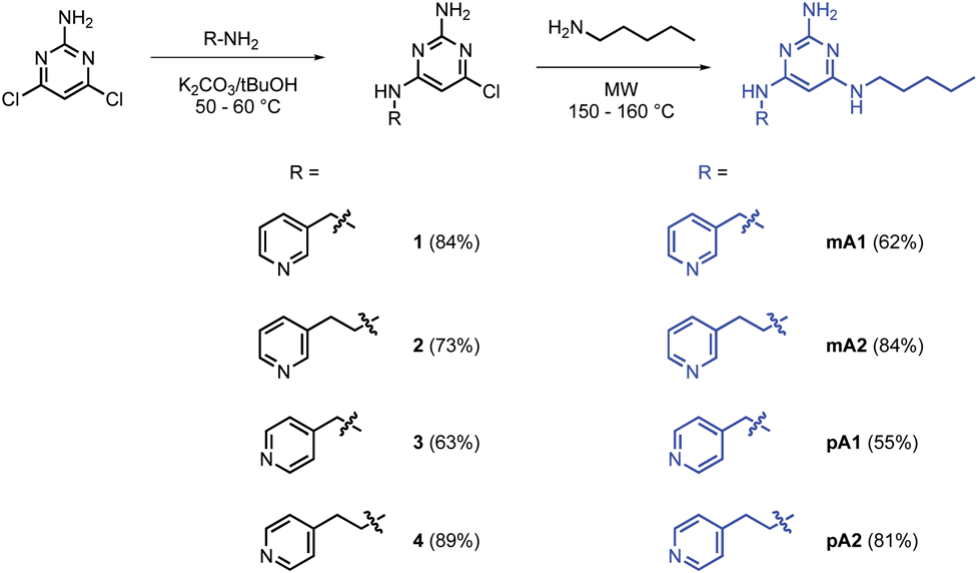
Synthesis of pyrimidine–pyridine ligands mA1, mA2, pA1 and pA2.

**Scheme 2:**
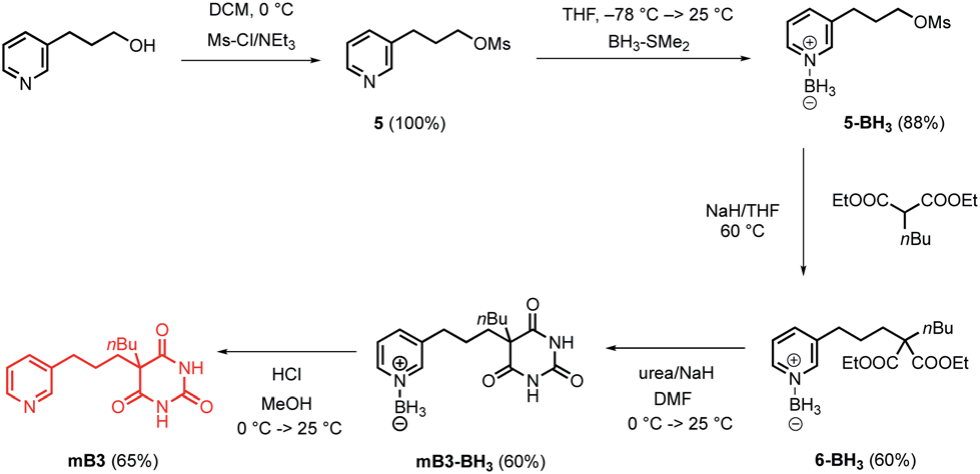
Synthesis of barbiturate–pyridine ligand mB3.

**Scheme 3:**
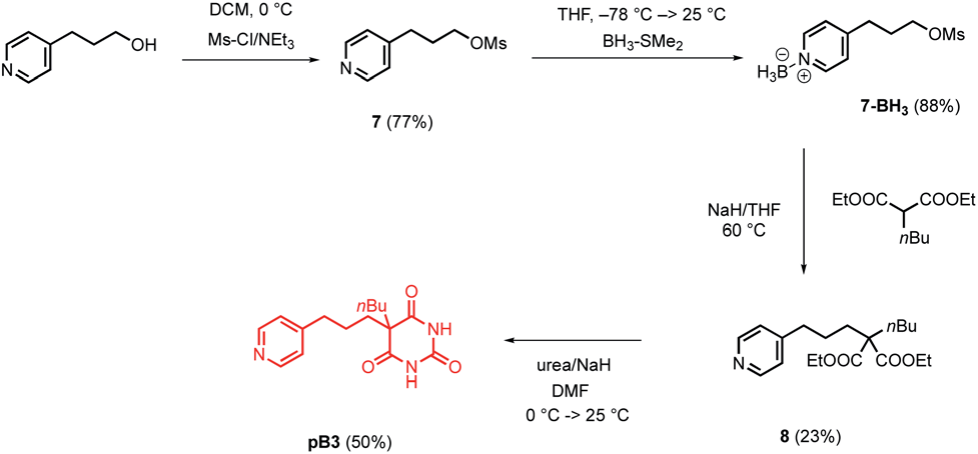
Synthesis of barbiturate–pyridine ligand pB3.

Pyrimidine–pyridine ligands mA1, mA2, pA1 and pA2 were synthesised from 4,6-dichloropyrimidin-2-amine in two steps as shown in Scheme 1. Barbiturate–pyridine ligands mB3 and pB3 were synthesised from 3-(pyridin-3-yl)propan-1-ol or 3-(pyridin-4-yl)propan-1-ol as shown in Schemes 2 and 3. In each case, the hydroxyl group was first converted to a mesylate, and then the pyridine was protected with BH_3_·SMe_2_ to avoid mixtures of alkylation products.^8^ The protected mesylates were treated with *n*-butyl diethylmalonate to produce malonates that were obtained in both the pyridine protected and deprotected forms. mB3 was obtained from the protected malonate 6-BH_3_ by reaction with urea followed by deprotection using HCl in methanol. pB3 was obtained from the deprotected malonate 8 by reaction with urea. The synthesis of c-P6 was described previously.^4,9^

**Fig. 4:**
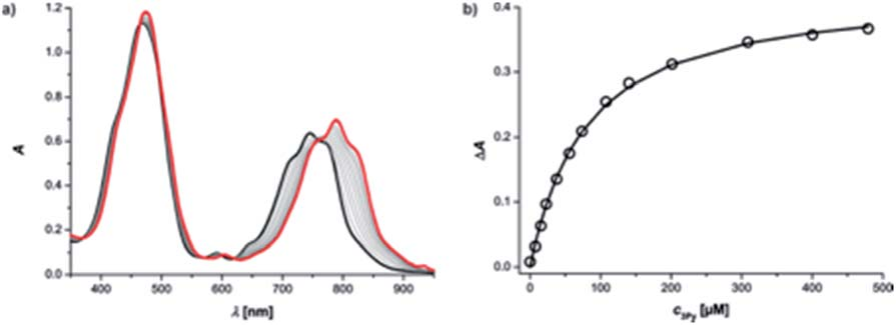
UV-vis-NIR titration (CHCl_3_, 298 K) of mPy into c-P6 (1.8 μM). (a) The initial UV-vis-NIR spectrum of c-P6 is shown as a thick black line, and the final spectrum of the complex is shown in red. (b) The change in absorption at 833 nm (circles) and the fit to a 1 : 1 binding isotherm (line) assuming six identical independent binding sites for c-P6.

Reference association constants for the interaction of a mono-dentate pyridine ligand with c-P6 were measured using 3-methylpyridine (mPy) and 4-methylpyridine (pPy). The UV-vis-NIR titration of mPy to c-P6 in chloroform at 298 K is shown in Fig. 4. The data fit well to a 1 : 1 binding isotherm, assuming that all six porphyrin units act independently and identically. The apparent 1 : 1 association constants are listed in Table 1.

**Table 1:** Apparent 1 : 1 association constants measured by UV-vis-NIR titrations in CHCl_3_ at 298 K for binding to c-P6^a^

Guest	log *K*/M^−1^	Guest mixture (1 : 1)	log *K*/M^−1^	Guest mixture (1 : 1)	log *K*/M^−1^
mPy	4.1 ± 0.1				
pPy	4.4 ± 0.1				
mB3	4.6 ± 0.1	mB3 + A	4.6 ± 0.3		
pB3	5.3 ± 0.1	pB3 + A	5.3 ± 0.1		
mA1	5.1 ± 0.2	mA1 + B	5.4 ± 0.1	mA1 + C	5.4 ± 0.1
mA2	5.5 ± 0.1	mA2 + B	5.5 ± 0.1	mA2 + C	5.7 ± 0.1
pA1	5.1 ± 0.1	pA1 + B	5.2 ± 0.4	pA1 + C	5.3 ± 0.2
pA2	5.2 ± 0.3	pA2 + B	5.3 ± 0.1	pA2 + C	5.5 ± 0.2

a Errors are quoted as two times the standard deviation based on one repetition.

These titrations serve as a benchmark for understanding the behaviour of the functionalised pyridine ligands. If the titration data for a ligand fit well to a 1 : 1 isotherm with an apparent 1 : 1 association constant that is comparable to the values found for mPy and pPy in Table 1, then we conclude that binding is non-cooperative. A significant increase in the value of the apparent 1 : 1 association constant obtained by fitting the titration data to a 1 : 1 isotherm implies that there are additional H-bonding interactions between bound ligands. If in addition the titration data do not fit well to a 1 : 1 isotherm, we conclude that the stepwise association constants for the binding of each ligand are not identical, and that there are complexes present, which have enhanced stability due to cooperative interactions between bound ligands. For example in the 1 : 6 complex, formation of a cyclic rosette-type structure would stabilise the binding of the sixth ligand relative to the first five. Thus all titration data for the functional ligands and ligand mixtures were first analysed using a 1 : 1 isotherm to assess whether cooperative H-bonding interactions between bound ligands were apparent.

The interaction of individual barbiturate–pyridine and pyrimidine–pyridine ligands with c-P6 was then investigated using UV-vis-NIR titrations in chloroform at 298 K. In all cases, the titration data fit well to a 1 : 1 binding isotherm, assuming that all six porphyrin units act independently and identically (see ESI Section S3.2†). The apparent 1 : 1 association constants listed in Table 1 show significant increases compared with the corresponding values for the reference ligands mPy and pPy. This result suggests that there are inter-ligand H-bonding interaction between the barbiturate or the pyrimidine moieties when the pyridine units coordinate to the zinc porphyrins in c-P6. Both barbiturates and pyrimidines have been reported to form multiply H-bonded rosette-type structures on their own, even though there are only 12 H-bonding interactions, and models suggest that cyclic assemblies with two H-bonds between each neighbouring ligand could fit inside c-P6 (see ESI Section S5†).^10^

**Fig. 5:**
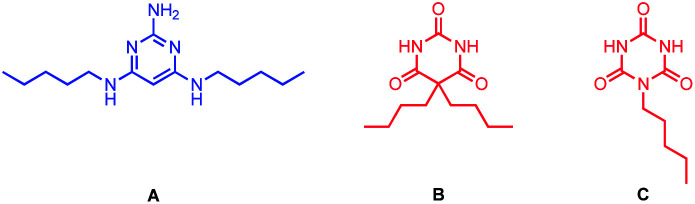
Pyrimidine A, barbiturate B, and cyanurate C.

Mixing of a barbiturate–pyridine ligand with pyrimidine A (see Fig. 5 for structure), should lead to assembly of a H-bonded rosette equipped with three pyridine ligands. Similarly, mixing of a pyrimidine–pyridine ligand with barbiturate B or with cyanurate C (see Fig. 5 for structures) should give rosettes equipped with three pyridine ligands, and these multivalent H-bonded assemblies should have a higher affinity for c-P6 than the corresponding monovalent ligands. Therefore ten different 1 : 1 mixtures of two rosette components that should each form a H-bonded trispyridine ligand were titrated into c-P6 in chloroform. In all cases, the UV-vis-NIR titration data fit well to a 1 : 1 binding isotherm, assuming that all six porphyrin units act independently and identically (see ESI Section S3.3†). The apparent 1 : 1 association constants based on the concentration of the pyridine-containing rosette component are listed in Table 1.

Compared with the corresponding monovalent reference pyridine ligands, no increase in stability was observed for binding of the trispyridine rosettes for any of the ten combinations. Different interpretations are possible. The structure of the trispyridine rosette may not be compatible with cooperative binding of all three ligands to c-P6, so the ligand components of the mixture may just bind in the same way as the individual ligands without incorporating the second component of the rosette. The rosette may be formed inside c-P6, but the increase in stability compared with the monovalent reference ligands mPy or pPy is similar to that achieved by the H-bonding interactions observed for the binding of the ligand components on their own. However, rosettes formed with cyanurates are known to be significantly more stable than rosettes formed with barbiturates.^7,11^Table 1 shows that there is no significant difference in stability for the complexes formed with mixtures containing B compared with the complexes formed with the corresponding mixtures containing C. This result suggests that binding of the pyridine-containing components on their own is the preferred mode of interaction in these systems, and no rosettes are actually formed inside c-P6.

**Fig. 6:**
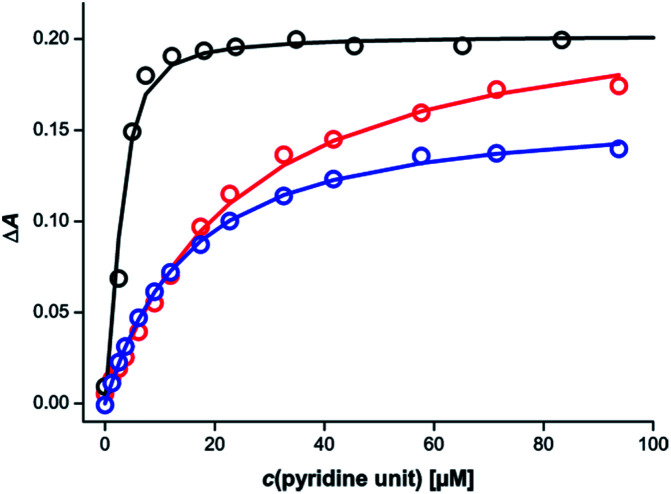
UV-vis-NIR titration data (CHCl_3_, 298 K) for addition of mB3 (red), mA1 (blue) or a 1 : 1 mixture of mB3 and mA1 (black) to c-P6 (0.7 μM). The absorption at 838 nm is shown as circles, and the lines are the calculated best fit to a 1 : 1 binding isotherm assuming that the six porphyrin units of c-P6 act identically and independently.

Different behaviour was observed for mixtures in which both components of the rosette were equipped with pyridine units. Fig. 6 shows titration data for addition of a 1 : 1 mixture of the barbiturate–pyridine ligand mB3 and the pyrimidine–pyridine ligand mA1 to c-P6 in chloroform. The corresponding titration data for addition of the individual components is also shown for comparison. It is clear that the rosette-forming mixture results in a much more stable complex than either of the two components alone.

Similar behaviour was observed for all eight combinations of the barbiturate–pyridine ligands and pyrimidine–pyridine ligands. Table 2 shows the apparent 1 : 1 association constants for the pyridine·zinc porphyrin interaction in these complexes.

**Table 2:** Apparent 1 : 1 association constants measured by UV-vis-NIR titrations in CHCl_3_ at 298 K for binding to c-P6^a^

Guest	log *K*/M^−1^	Guest	log *K*/M^−1^	Guest mixture (1 : 1)	log *K*/M^−1^
mB3	4.6 ± 0.1	mA1	5.1 ± 0.2	mB3 + mA1	6.1 ± 0.2
mB3	4.6 ± 0.1	mA2	5.5 ± 0.1	mB3 + mA2	5.9 ± 0.1
mB3	4.6 ± 0.1	pA1	5.1 ± 0.1	mB3 + pA1	6.2 ± 0.3
mB3	4.6 ± 0.1	pA2	5.2 ± 0.3	mB3 + pA2	5.9 ± 0.1
pB3	5.3 ± 0.1	mA1	5.1 ± 0.2	pB3 + mA1	6.0 ± 0.2
pB3	5.3 ± 0.1	mA2	5.5 ± 0.1	pB3 + mA2	6.6 ± 0.2
pB3	5.3 ± 0.1	pA1	5.1 ± 0.1	pB3 + pA1	6.1 ± 0.1
pB3	5.3 ± 0.1	pA2	5.2 ± 0.3	pB3 + pA2	6.5 ± 0.1

a Errors are quoted as two times the standard deviation based on one repetition.

In all cases, the value is significantly higher than the apparent 1 : 1 association constants for binding of either of the individual ligands, which suggests that rosette assembly does indeed take place inside c-P6 for all of these systems.

However in many cases, the fit of the titration data to the 1 : 1 binding isotherm was poor (ESI Section S3.3†). The titration data were therefore analysed in more detail based on a 1 : 3 : 3 stoichiometry for the c-P6 : barbiturate : pyrimidine complexes. Global analysis multiple regression was used to fit the entire spectrum between 600 and 950 nm, giving the equilibrium constants and the spectra of the species involved. Two different models were used to fit the data :  one model where all ligands bind in one step to give the 1 : 3 : 3 complex (all-or-nothing, ESI Section S3.4†), and another where the ligands bind in pairs to form 1 : 1 : 1, 1 : 2 : 2 and 1 : 3 : 3 complexes (stepwise, ESI Section S3.5†). In all cases, the fit to the stepwise model is significantly better than the fit to the all-or-nothing model (ESI Section S3.6†). There are many different stepwise models that could be used to fit the titration data, so it is not possible to interpret the results of the fitting in more detail. However, it is clear that there are intermediate species with lower porphyrin  :  ligand stoichiometry that are particularly stable, even though the 1 : 3 : 3 complex is eventually formed. Table 3 gives the overall equilibrium constants for formation of the 1 : 3 : 3 complexes from the three components based on the best fit to the stepwise model. The values are very similar for all eight complexes, which indicates that the variations in the structure of the linker connecting the rosette and the pyridine ligands have little effect in this system.

**Table 3:** Overall formation constants (*K*_f_) for assembly of 1 : 3 : 3 c-P6·A_3_·B_3_ complexes based on the best fit of the UV-vis-NIR titration data in CHCl_3_ at 298 K to a stepwise binding isotherm^a^

Guest mixture (1 : 1)	log *K*_f_/M^−6^	EM/M
mB3 + mA1	37.0 ± 0.5	0.21 ± 0.05
mB3 + mA2	36.6 ± 0.2	0.17 ± 0.03
mB3 + pA1	37.9 ± 0.9	0.21 ± 0.08
mB3 + pA2	37.2 ± 0.9	0.15 ± 0.06
pB3 + mA1	37.9 ± 0.3	0.21 ± 0.04
pB3 + mA2	37.7 ± 1.0	0.19 ± 0.08
pB3 + pA1	38.6 ± 0.4	0.19 ± 0.04
pB3 + pA2	38.6 ± 0.2	0.19 ± 0.04

a Errors are quoted as two times the standard deviation based on one repetition.

The overall equilibrium constants for formation of the 1 : 3 : 3 complexes in Table 3 were used to determine the values of EM for interaction of the hexapyridine rosettes with c-P6 (eqn (1)).

1 *K*_f_ = *K*_HB_ × *K*_L1_^3^ × *K*_L2_^3^ × EM^5^

where *K*_HB_ is the association constant for the formation of the rosette from six ligands, *K*_L1_ and *K*_L2_ are association constants for the corresponding intermolecular pyridine·zinc porphyrin interaction, and EM is the mean effective molarity for the five intramolecular pyridine·zinc porphyrin interactions in the 1 : 3 : 3 complex.

The value of *K*_HB_ was determined previously as 6.3 ± 0.1 × 10^15^ M^−5^ in chloroform solution,^7^ and the association constants for the reference ligands mPy and pPy were used to estimate *K*_L1_ and *K*_L2_. The resulting values of EM listed in Table 3 are all very similar (approximately 200 mM) and are in accord with the most commonly observed range of supramolecular EM values.^3^ The values of EM quoted are not statistically corrected for the symmetry of the complexes, but statistical corrections do not significantly affect the values of EM for this system. It appears that the special cooperative effects leading to the very high values of EM observed for T are lost in the more flexible H-bonded rosette ligands.

**Fig. 7:**
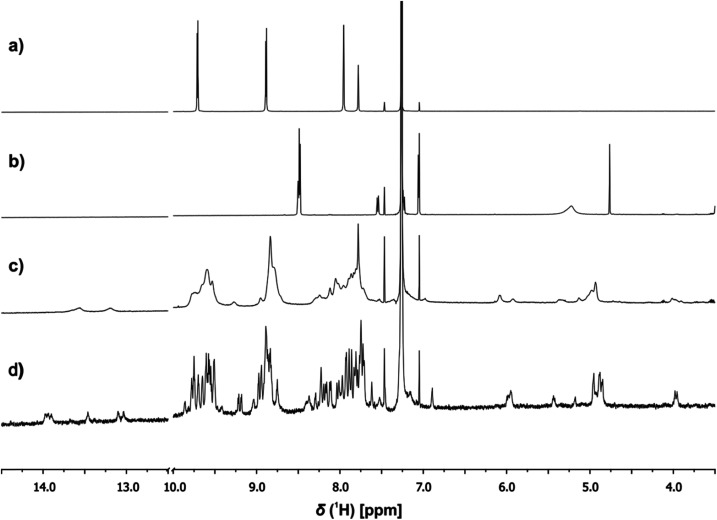
Partial ^1^H NMR (500 MHz, CDCl_3_) spectra of (a) c-P6 (0.16 mM, 298 K), (b) a 1 : 1 mixture of pB3 and mA2 (0.48 mM, 298 K), (c) a 1 : 3 : 3 mixture of c-P6 (0.16 mM), pB3 and mA2 (0.48 mM) at 298 K, and (d) a 1 : 3 : 3 mixture of c-P6 (0.16 mM), pB3 and mA2 (0.48 mM) at 233 K.

^1^H NMR spectroscopy was used to investigate the structures of the complexes. Fig. 7c shows an example of the ^1^H NMR spectrum of a 1 : 3 : 3 mixture of c-P6, pB3 and mA2 in chloroform solution at room temperature. The spectrum is very broad and shows a number of highly shifted signals compared with the corresponding spectra of c-P6 (Fig. 7a) and of a 1 : 1 mixture of pB3 and mA2 (Fig. 7b). At concentrations below 1 mM, the rosette does not assemble under these conditions, and the characteristic signals due to H-bonded barbiturate NH protons at 13–14 ppm are not observed for the mixture of the two rosette components pB3 and mA2 in the absence of porphyrin (Fig. 7b). However, in the presence of c-P6, two broad signals are observed between 13 and 14 ppm, which indicates that addition of the porphyrin induces assembly of the H-bonded rosette. Fig. 7c also shows a number of new signals between 4 and 6 ppm in the mixture, which suggests that the rosette is formed inside the macrocycle, and the signals due to the ligands are shifted upfield by the ring current of the porphyrins.

When the 1 : 3 : 3 mixture was cooled to 233 K a sharper, more complicated spectrum was obtained (Fig. 7d). There are at least six non-equivalent H-bonded barbiturate NH signals between 13 and 14 ppm, and the complexity of the spectrum indicates that the symmetry of both the porphyrin nanoring and the rosette are lost due to slow exchange processes in the complex. As shown in the side views of the complex in Fig. 3, the H-bonded rosette must sit out of the plane of the c-P6 ring in order to accommodate the pyridine ligands, and this lowers the symmetry of the porphyrins. The substitution pattern on the pyrimidine is not symmetric, which means that different isomers of the rosette are possible with respect to the relative positions of the pentyl chains and pyridine substituents. In addition, the barbiturate is prochiral, which may result in increased complexity due to diastereotopicity. A more detailed assignment of the spectrum was not possible, and although further structural information could not be obtained from NMR or mass spectrometry, ^1^H NMR DOSY spectra did confirm that the porphyrin and ligand components have the same diffusion coefficient (see ESI Section S4†).

## Conclusions

These experiments demonstrate cooperative assembly of a 7-component supramolecular complex at micromolar concentrations using a combination of H-bonding and metal–ligand coordination interactions. 1 : 1 mixtures of barbiturate and pyrimidine derivatives, each equipped with a pyridine ligand, assemble into hexapyridine rosette ligands *via* cooperative H-bonding interactions. These multidentate ligands bind cooperatively to a hexaporphyrin nanoring *via* zinc-pyridine coordination. UV-vis-NIR spectra show that 1 : 1 mixtures of the rosette-forming ligands bind to the porphyrin nanoring at concentrations at which the individual pyridine ligands do not coordinate zinc porphyrins. ^1^H NMR spectra show that 1 : 1 mixtures of the ligands form H-bonded rosettes in the presence of the porphyrin nanoring at concentrations at which H-bonds are not formed in the absence of porphyrin. Overall formation constants for assembly of the rosette-nanoring complex from the three components were used to determine average values of EM of about 200 mM for the interaction of the hexapyridine rosettes with the nanoring. The low value of EM compared with the rigid covalent ligand T is probably due to the flexible linker connecting the rosette components to the pyridine ligands. However, neither the length nor the geometry of the linkers that connect the pyridine units to the H-bonding moieties of the rosette have a significant effect on the overall stability of the assemblies.

## Conflicts of interest

There are no conflicts to declare.

## Supplementary Material

SC-012-D0SC06097F-s001

## References

[cit1] Whitty A. (2008). Nat. Chem. Biol..

[cit2] Hunter C. A., Anderson H. L. (2009). Angew. Chem., Int. Ed..

[cit3] Motloch P., Hunter C. A. (2016). Adv. Phys. Org. Chem..

[cit4] Hoffmann M., Kärnbratt J., Chang M.-H., Herz L. M., Albinsson B., Anderson H. L. (2008). Angew. Chem., Int. Ed..

[cit5] Hogben H. J., Sprafke J. K., Hoffmann M., Pawlicki M., Anderson H. L. (2011). J. Am. Chem. Soc..

[cit6] Mathias J. P., Simanek E. E., Zerkowski J. A., Seto C. T., Whitesides G. M. (1994). J. Am. Chem. Soc..

[cit7] Motloch P., Hunter C. A. (2020). Org. Biomol. Chem..

[cit8] Zajac M. A. (2008). J. Org. Chem..

[cit9] Sprafke J. K., Kondratuk D. V., Wykes M., Thompson A. L., Hoffmann M., Drevinskas R., Chen W.-H., Yong C. K., Kärnbratt J., Bullock J. E., Malfois M., Wasielewski M. R., Albinsson B., Herz L. M., Zigmantas D., Beljonne D., Anderson H. L. (2011). J. Am. Chem. Soc..

[cit10] Lin X., Suzuki M., Gushiken M., Yamauchi M., Karatsu T., Kizaki T., Tani Y., Nakayama K.-i., Suzuki M., Yamada H., Kajitani T., Fukushima T., Kikkawa Y., Yagai S. (2017). Sci. Rep..

[cit11] ten Cate M. G. J., Huskens J., Crego-Calama M., Reinhoudt D. N. (2004). Chem.–Eur. J..

