# Supplementary material for: Cooperative assembly of H-bonded rosettes inside a porphyrin nanoring
Source: Chem Sci. 2020 Dec 8;12(4):1427–32. doi: 10.1039/d0sc06097f (PMC8179033; doi:10.1039/d0sc06097f)
Supplement: SC-012-D0SC06097F-s001 [file SC-012-D0SC06097F-s001.pdf]

Electronic Supplementary Material (ESI) for Chemical Science.
This journal is © The Royal Society of Chemistry 2020

SUPPORTING INFORMATION

Cooperative Assembly of H-bonded Rosettes Inside a Porphyrin Nanoring

Petr Motloch,[a] Pernille S. Bols,[b] Harry L. Anderson,[b,*] and Christopher A. Hunter [a,*]

[a] Department of Chemistry, University of Cambridge, Lensfield Road, Cambridge CB2 1EW (UK).
[b] Department of Chemistry, Chemistry Research Laboratory, Oxford University, Oxford OX1 3TA (UK)

Email: herchelsmith.orgchem@ch.cam.ac.uk , harry.anderson@chem.ox.ac.uk

Table of Contents

S1: General experimental 2
S2: Synthesis 3
S3: UV-vis-NIR titrations 44
S4: DOSY Spectrum 72
S5: Molecular Modelling 73
S6: References 76

S1: General experimental

The syntheses of **B**, **C**, **A** and ***c*-P6** were described previously.[1,2] The chemicals were purchased from commercial suppliers and used without further purifications unless stated otherwise. Solvents were either distilled before use or used as obtained. For chromatography, automatic chromatography systems CombiFlash R_f^+ and CombiFlash R_f^+ Lumen (with UV light detection at 254 nm and 280 nm and evaporative light scattering detector for Lumen) with pre-packed puriFlash columns from Interchim (silica, 25 μm) with a loading of mixtures on Celite were used. The microwave used was Biotage Initiator⁺. The reactions were monitored either by glass TLC plates coated with silica gel 60 F_{254} (Merck) and the plates were inspected by UV light (254 nm) or by LCMS Waters Acquity H-class UPLC coupled with a single quadrupole Waters SQD2 with the conditions as follows: UPLC Column (see below), solvent A: Water + 0.1% formic acid; solvent B: acetonitrile or THF (see below) + 0.1% formic acid; gradient and flow rate (see below); column temperature of 40 °C, the signal was monitored at 254 nm and 280 nm.

Columns

Col1: ACQUITY UPLC CSH C18 Column, 130Å, 1.7 μm, 2.1 mm X 50 mm
Col3: ACQUITY UPLC HSS T3 Column, 100Å, 1.8 μm, 2.1 mm X 50 mm

Methods

MeCN-FAST: Gradient: 0 – 2 minutes 5% – 100%B + 1 minute 100%B
Flow rate: 0.6 ml/min

MeCN-SLOW: Gradient: 0 – 4 minutes 5% – 100%B + 1 minute 100%B
Flow rate: 0.6 ml/min

THF-FAST: Gradient: 0 – 2 minutes 5% – 80%B + 1 minute 80%B
Flow rate: 0.4 ml/min

THF-SLOW: Gradient: 0 – 4 minutes 5% – 80%B + 1 minute 80%B
Flow rate: 0.4 ml/min

THF_FAST_5%-35%: Gradient of 0-2 minutes 5% – 35%B + 1 minute 100%B
Flow rate: 0.4 ml/min

^{1}H and ^{13}C NMR spectra were recorded on Bruker 400 MHz Avance III HD SmartProbe Spectrometers at 400 MHz for ^{1}H, 128 MHz for ^{11}B, 101 MHz for ^{13}C or on a Bruker 500 MHz AVIII HD SmartProbe Spectrometer at 500 MHz for ^{1}H and 126 MHz for ^{13}C. All chemicals shift are quoted in ppm and were referenced to the residual peaks of used solvents: $CDCl_3$ (^{1}H: 7.26 ppm; ^{13}C: 77.00 ppm), CD_3OD (^{1}H: 3.31 ppm; ^{13}C: 49.00 ppm), d^6-DMSO (^{1}H: 2.50 ppm; ^{13}C: 39.52 ppm). Coupling constants *J* are stated in Hz. FT-IR spectra were measured on a Bruker Alpha spectrometer. HR-MS spectra were obtained on a Waters Xevo G2-S, Waters Vion IMS Qtof or Waters LCT Premier by electrospray-ionisation of samples. Melting points were recorder on a Mettler-Toledo MP90 system. Elemental analysis was performed by the Microanalysis facility at the Department of Chemistry at the University of Cambridge.

S2: Synthesis

Synthesis of 1

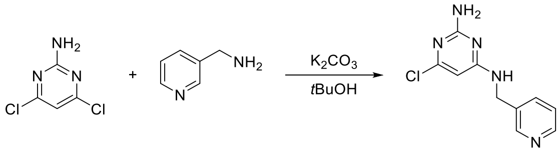

A mixture of 4,6-dichloropyrimidin-2-amine (689 mg, 4.20 mmol), 3-picolylamine (1 mL, 1 g, 9.8 mmol), K_2CO_3 (437 mg, 3.17 mmol) and *t*BuOH (15 mL) were heated at 50 °C for 4 days. The mixture was evaporated and water (20 mL) was added. The mixture was then sonicated for 2 minutes. The obtained solid was collected by suction and dried overnight in vacuum oven at 40 °C to provide the title compound (831 mg, 84% yield) as a white solid.

^{1}H NMR ($CD_3OD/CDCl_3$, 400 MHz, 298 K): δ 8.47 (d, J = 1.5 Hz, 1H), 8.39 (d, J = 3.5 Hz, 1H), 7.74 (d, J = 8.0 Hz, 1H), 7.33 (dd, J = 8.0, 5.0 Hz, 1H), 5.85 (s, 1H), 4.53 (s, 2H).
^{13}C NMR (d^6-DMSO, 126 MHz, 298 K): δ 164.0, 163.0, 157.6, 149.0, 148.2, 135.4, 135.2, 123.6, 92.8, 41.08.

HR-MS (ESI): Calculated for $C_{10}H_{11}N_5Cl$ $[M+H]^+$ 236.0703, found: 236.0693 (Δ = 4.2 ppm)

FT-IR (thin film): 1640, 1575, 1551, 1469, 1427, 1369, 1331, 1150 cm^{-1}.

MP: 198 – 200 °C

LCMS Method: Col3_ MeCN_FAST_5%-35%

^{1}H NMR

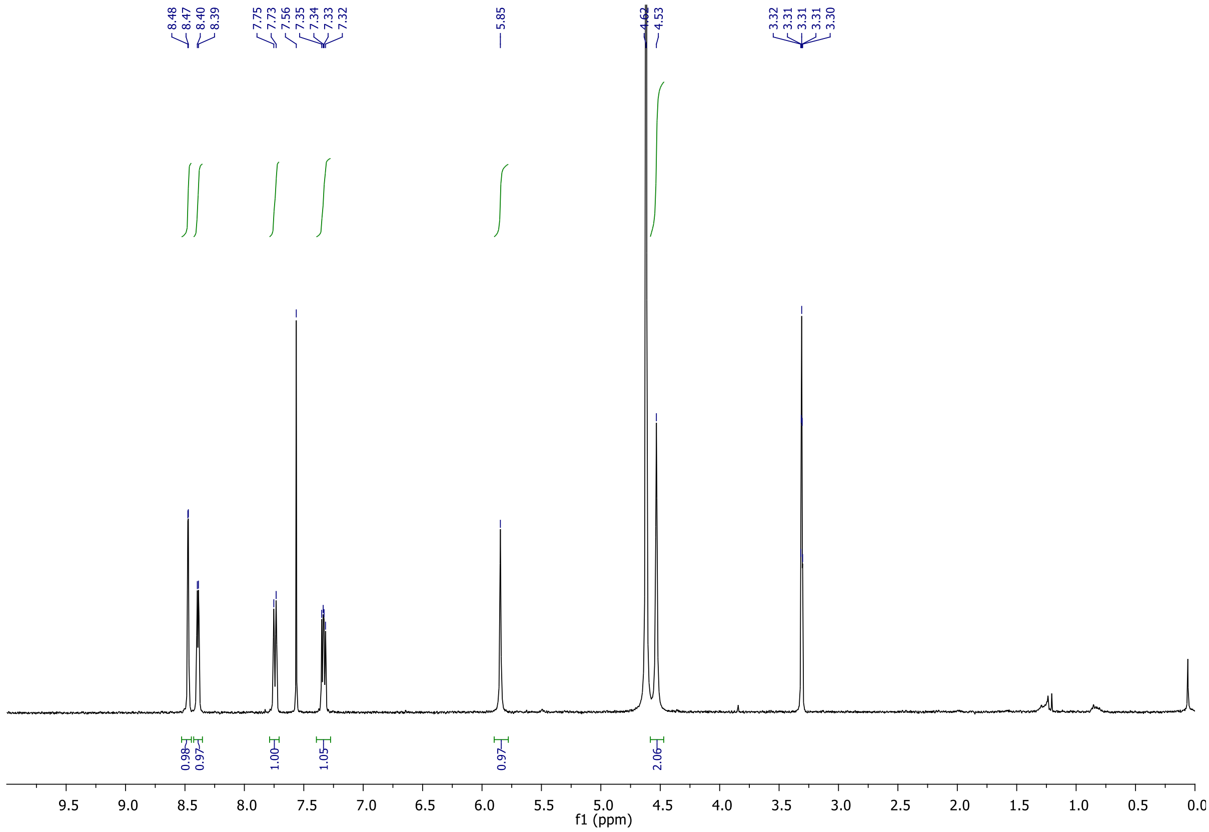

^{13}C NMR

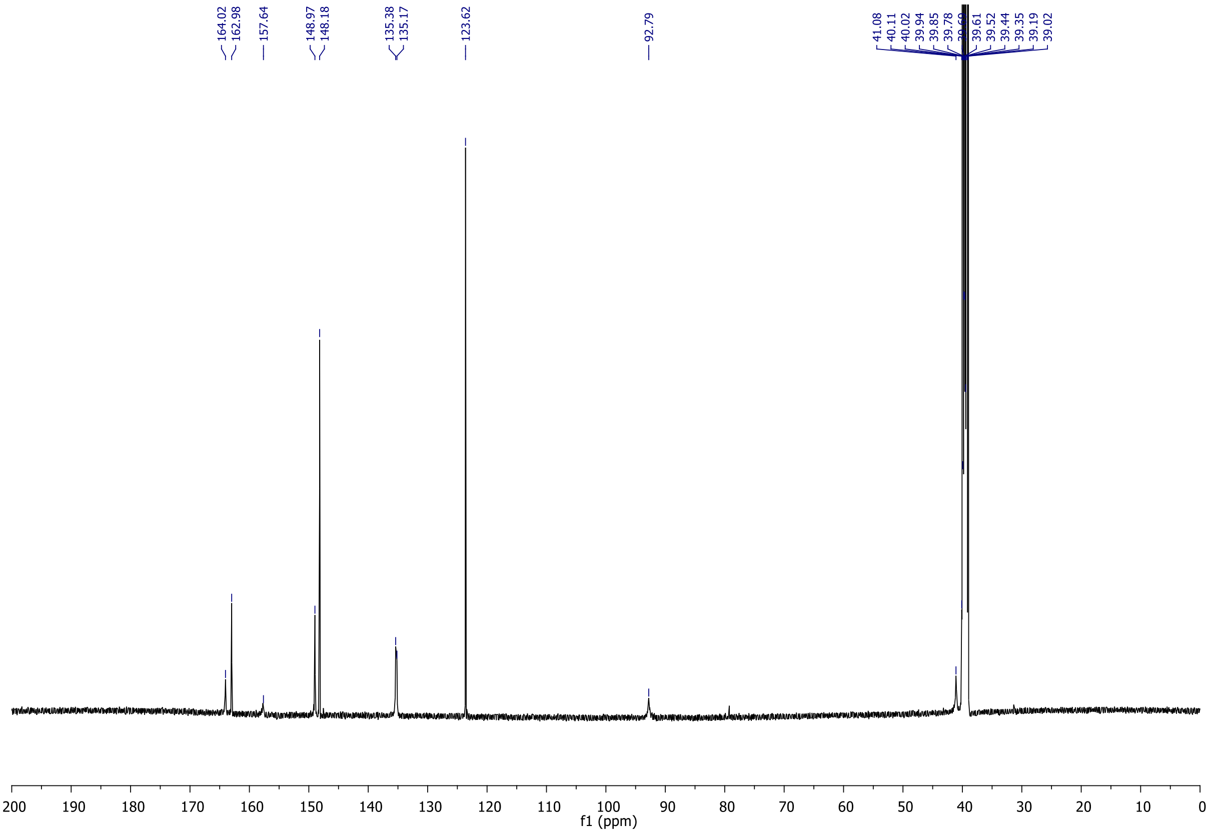

Synthesis of 2

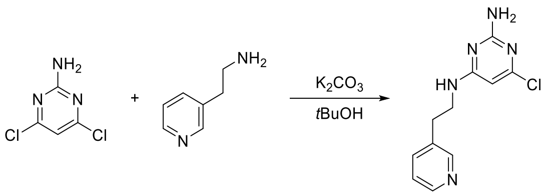

A mixture of 4,6-dichloropyrimidin-2-amine (776 g, 4.64 mmol), 3-(2-aminoethyl)pyridine (1 mL, 1 g, 7.5 mmol), K_2CO_3 (490 mg, 3.55 mmol) and *t*BuOH (18 mL) were heated at 50 °C for 2 days. The mixture was evaporated and water (20 mL) was added. The mixture was then sonicated for 30 minutes. The obtained solid was collected by suction and dried overnight in vacuum oven at 40 °C to provide the title compound (840 mg, 73% yield) as a pale yellow solid.

^{1}H NMR (CD_3OD, 400 MHz, 298 K): δ 8.41 (d, J = 2.0 Hz, 1H), 8.37 (dd, J = 5.0, 1.5 Hz, 1H), 7.74 (dt, J = 8.0, 1.5 Hz, 1H), 7.36 (dd, J = 8.0, 5.0 Hz, 1H), 5.78 (s, 1H), 3.59 (s, 2H), 2.92 (t, J = 7.0 Hz, 2H).
^{13}C NMR (d^6-DMSO, 126 MHz, 298 K): δ 164.1, 163.1, 157.3, 150.0, 147.5, 136.5, 135.1, 123.6, 92.8, 41.3, 32.0.

HR-MS (ESI): Calculated for $C_{11}H_{13}N_5Cl$ $[M+H]^+$ 250.0859, found: 250.0850 (Δ = 3.6 ppm)

FT-IR (thin film): 3276, 2371, 1575, 1478, 1425, 1362 cm^{-1}.

MP: 152 – 157 °C

LCMS Method: Col3_MeCN_FAST_5%-35%

^{1}H NMR

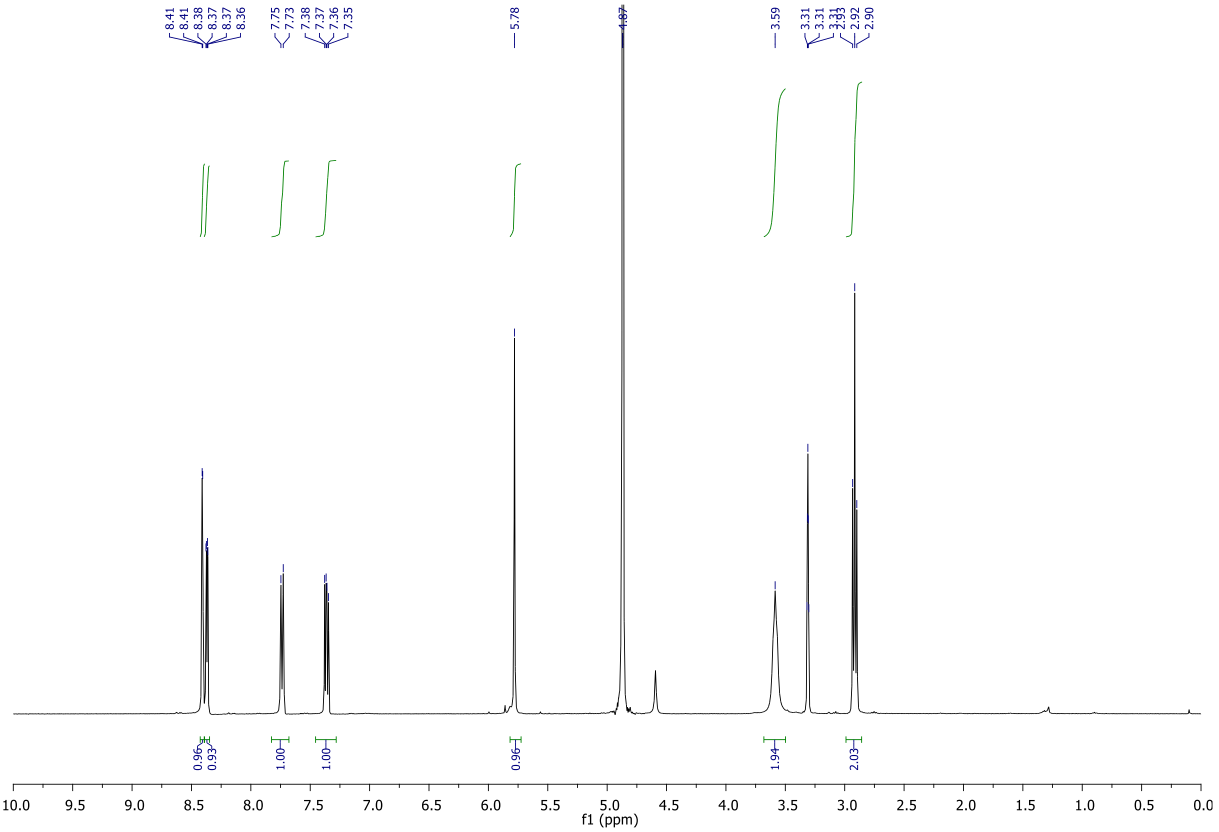

^{13}C NMR

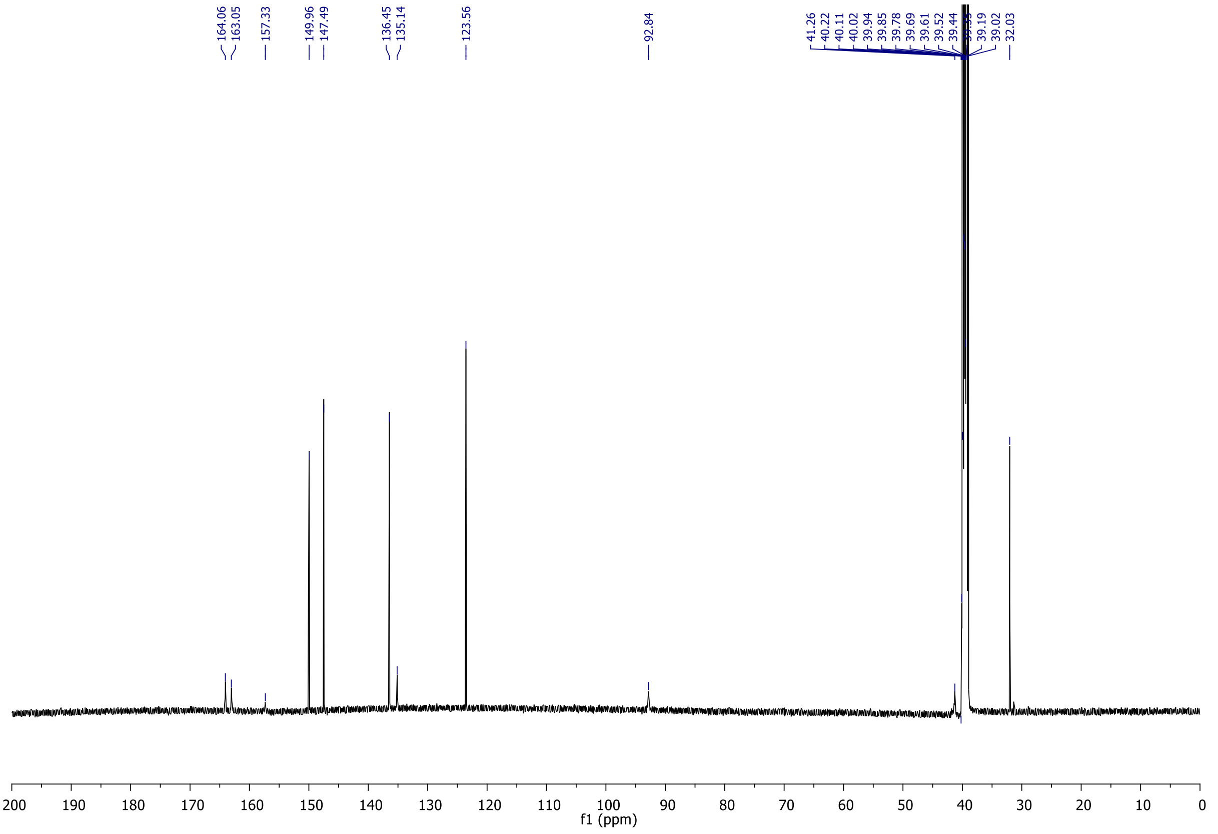

Synthesis of 3

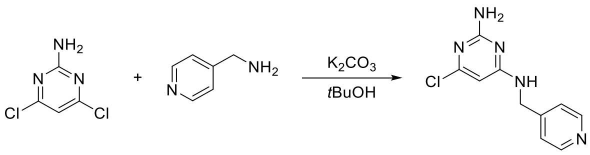

A mixture of 4,6-dichloropyrimidin-2-amine (800 mg, 4.88 mmol), 4-picolylamine (3 mL, 3.18 g, 29 mmol), K_2CO_3 (676 mg, 4.89 mmol) and *t*BuOH (15 mL) were heated at 60 °C overnight. The mixture was evaporated and water (30 mL) was added. The mixture was then sonicated for 30 minutes and the mixture dissolved. After standing, the form solid was collected and dried in the vacuum oven overnight to provid the title compound (727 mg, 63% yield) as a pale yellow solid.

^{1}H NMR (CD_3OD, 400 MHz, 298 K): δ 8.44 (d, J = 6.0 Hz, 2H), 7.35 (d, J = 6.0 Hz, 2H), 5.90 (s, 1H), 4.60 (s, 2H).
^{13}C NMR (d^6-DMSO, 126 MHz, 298 K): δ 164.2, 163.0, 157.8, 149.6, 148.9, 122.3, 92.8, 42.3.

HR-MS (ESI): Calculated for $C_{10}H_{11}N_5Cl$ $[M+H]^+$ 236.0697, found: 236.0694 (Δ = 1.5 ppm)

FT-IR (thin film): 3312, 3200, 2387, 1573, 1556, 1496, 1475, 1417, 1360, 1314, 1249 cm^{-1}.

MP: > 136 °C (carbonisation)

LCMS Method: Col3_ MeCN_FAST_5%-35%

^{1}H NMR

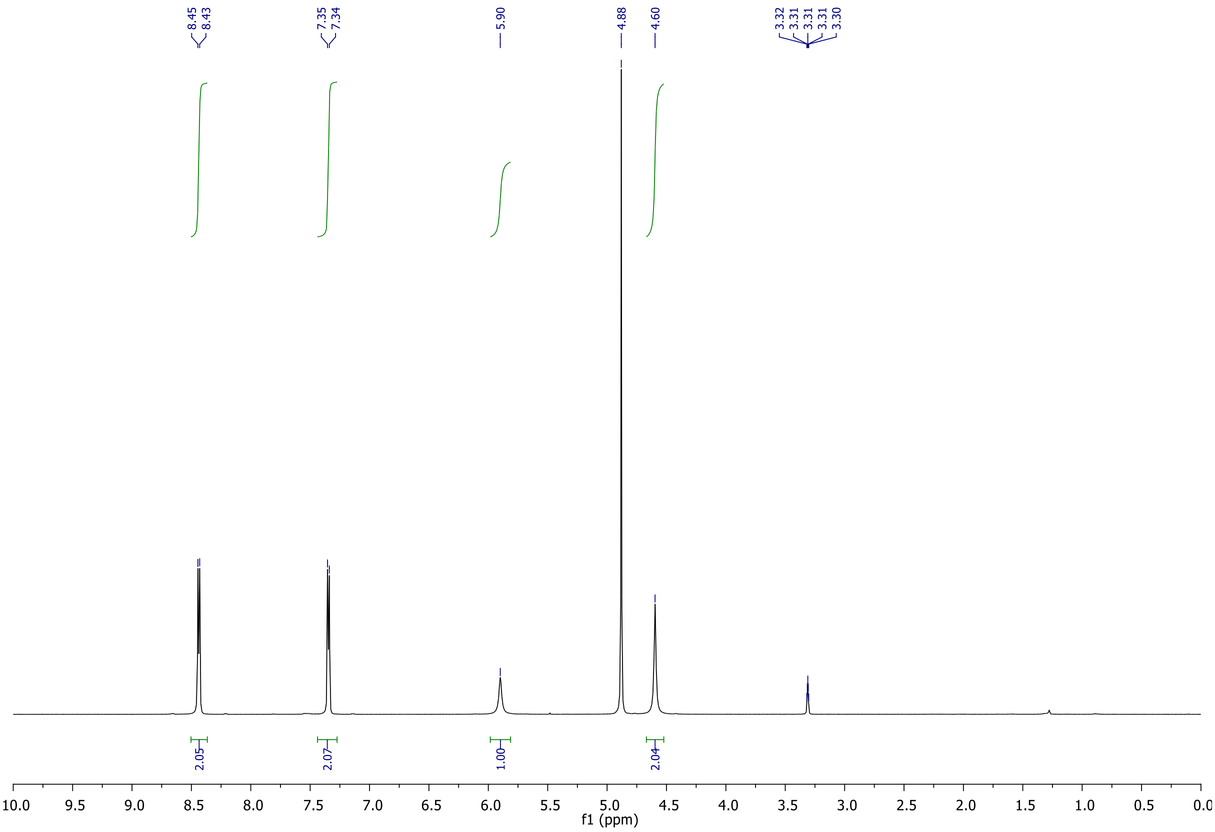

^{13}C NMR

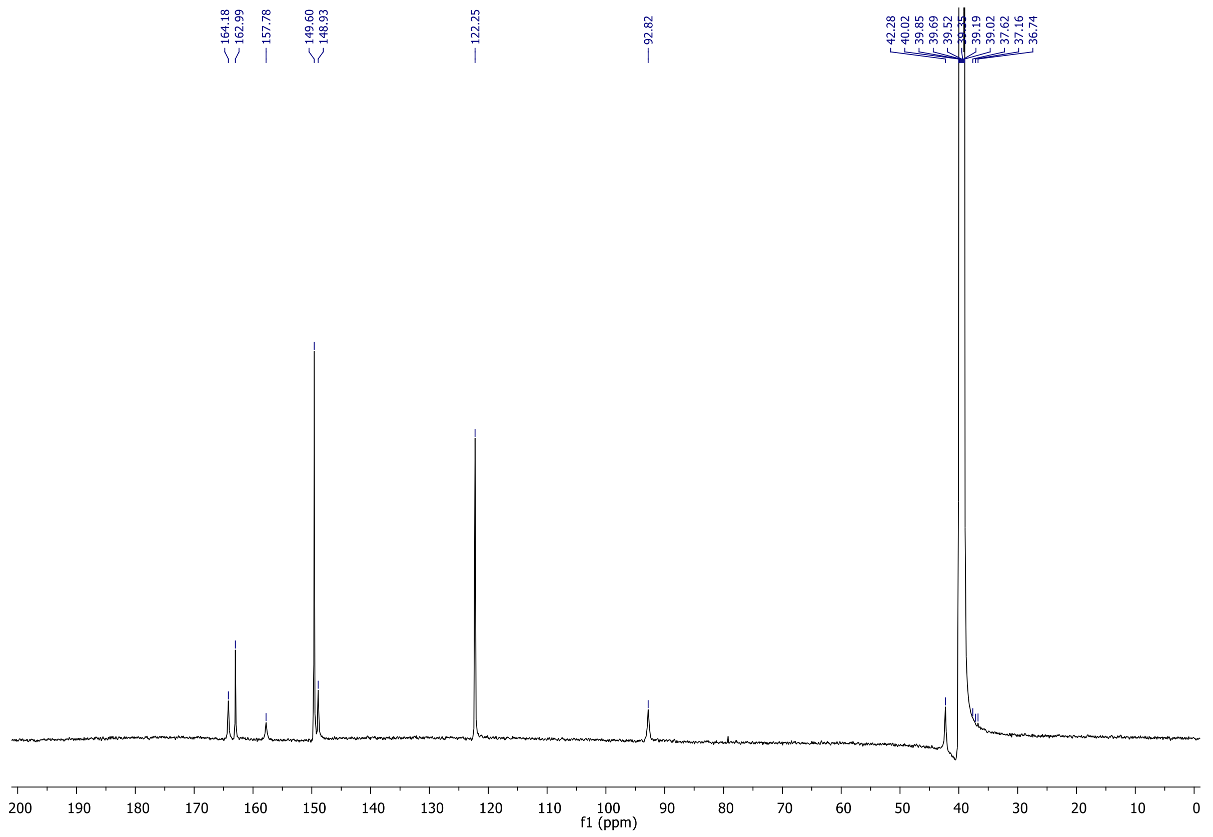

Synthesis of 4

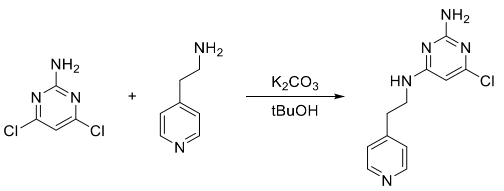

A mixture of 4,6-dichloropyrimidin-2-amine (1.06 g, 6.46 mmol), 4-(2-aminoethyl)pyridine (1.2 mL, 1.2 g, 10 mmol), K_2CO_3 (0.76 g, 5.5 mmol) and *t*BuOH (15 mL) were heated at 50 °C for 3 days. The mixture was evaporated and water (30 mL) was added. The mixture was then sonicated for 20 minutes. The obtained solid was collected by suction and dried overnight in vacuum oven at 40 °C to provide the title compound (1.43 g, 89% yield) as a yellow solid.

^{1}H NMR (CD_3OD, 400 MHz, 298 K): δ 8.43 – 8.42 (m, 2H), 7.33 (d, J = 6.0 Hz, 2H), 5.79 (s, 1H), 3.61 (s, 2H), 2.93 (t, J = 7.0 Hz, 2H).
^{13}C NMR (CD_3OD, 101 MHz, 298 K): δ 165.7, 164.4, 162.8, 151.7, 149.9, 126.1, 94.8, 41.9, 35.8.

HR-MS (ESI): Calculated for $C_{11}H_{13}N_5Cl$ $[M+H]^+$ 250.0854, found: 250.0850 (Δ = 1.4 ppm)

FT-IR (thin film): 3277, 2397, 1574, 1481, 1420, 1362 cm^{-1}.

MP: 157 °C (carbonisation)

LCMS Method: Col3_MeCN_FAST_5%-35%

^{1}H NMR

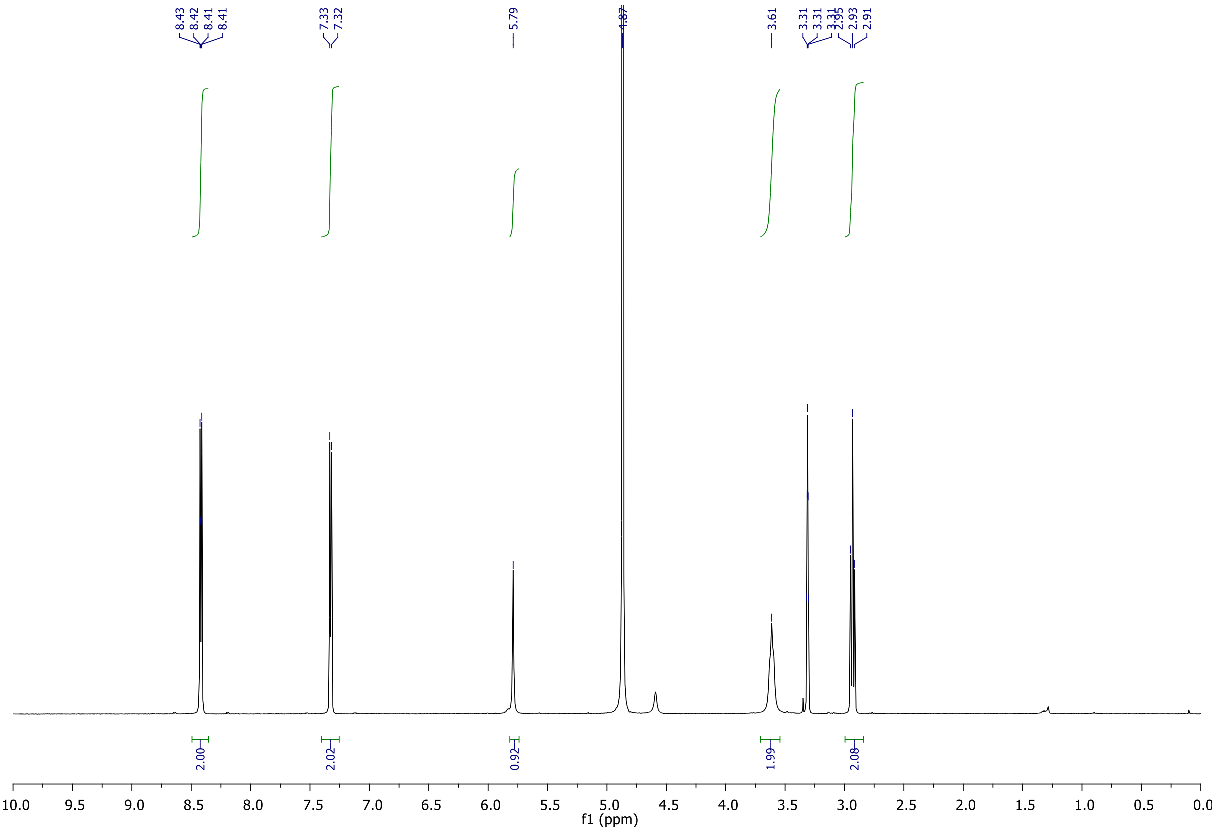

^{13}C NMR

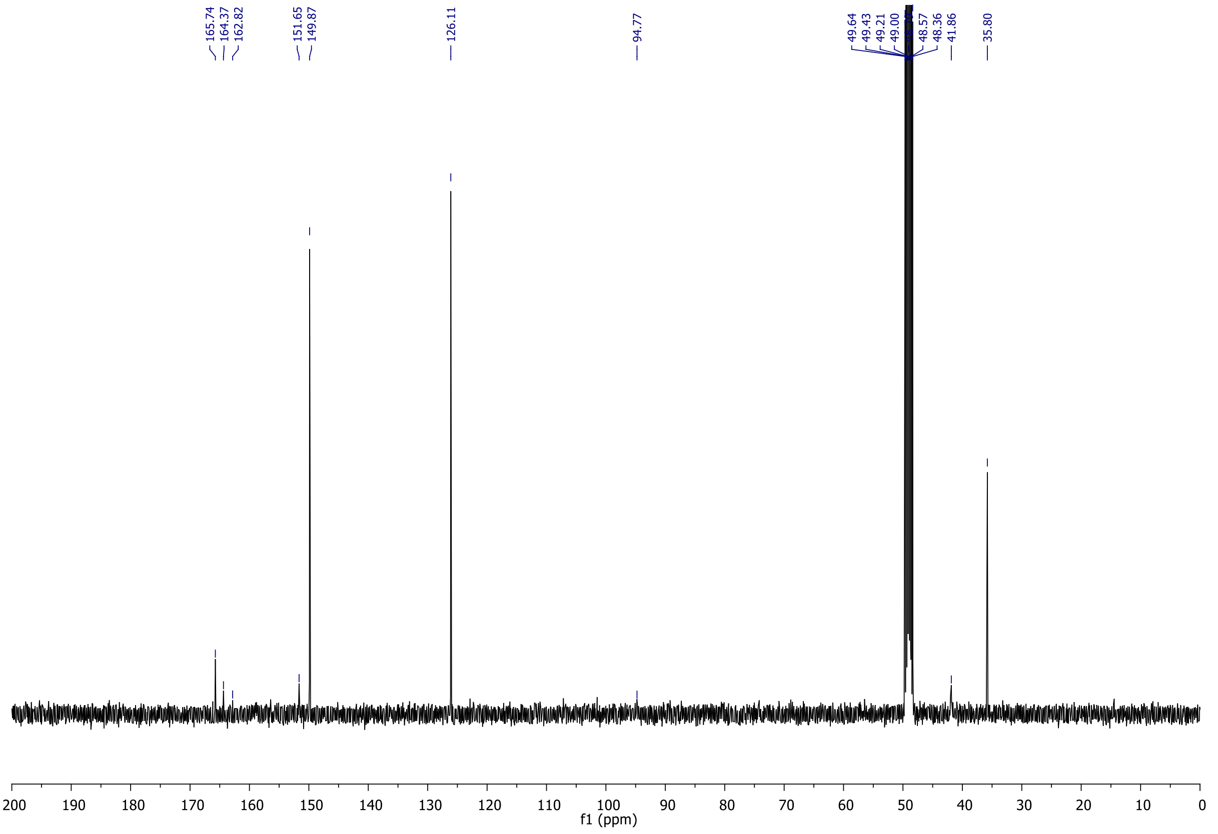

Synthesis of mA1

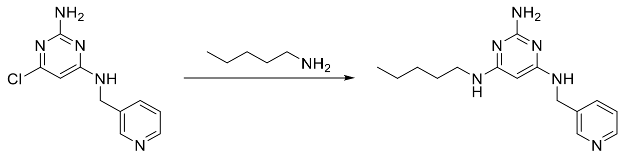

1 (337 mg, 1.43 mmol) was flushed with nitrogen in a MW vial. *n*-Pentylamine (1 mL, 0.9 g, 10 mmol) was added and the mixture was heated in microwave at 160 °C for 2 hours. A saturated solution of $NaHCO_3$ (15 mL) was added and the mixture was extracted with CH_2Cl_2/MeOH (20:1, 2 × 50mL) and CH_2Cl_2 (2 × 50 mL). The organic phase was dried over $MgSO_4$, evaporated and loaded to Celite. A combiflash of the residue on silica (CH_2Cl_2/MeOH: MeOH 0%→20%) provided the title compound (253 mg, 62% yield) as yellowish oil.

^{1}H NMR (CD_3OD, 400 MHz, 298 K): δ 8.50 (s, 1H), 8.38 (d, J = 4.0 Hz, 1H), 7.77 (d, J = 7.5 Hz, 1H), 7.34 (dd, J = 7.0, 5.0 Hz, 1H), 4.89 (s, 1H), 4.46 (s, 2H), 3.08 (t, J = 7.0 Hz, 2H), 1.52 – 1.43 (m, 2H), 1.29 (d, J = 2.5 Hz, 4H), 0.88 (t, J = 6.0 Hz, 3H).
^{13}C NMR (CD_3OD, 101 MHz, 298 K): δ 165.2, 165.1, 164.0, 149.1, 148.4, 137.7, 137.1, 125.1, 74.0, 43.2, 42.3, 30.3, 30.1, 23.5, 14.4.

HR-MS (ESI): Calculated for $C_{15}H_{23}N_6$ $[M+H]^+$ 287.1979, found: 287.1976 (Δ = 0.98 ppm)

FT-IR (thin film): 3309, 2955, 2928, 2858, 1575, 1505, 1455, 1424, 1355 cm^{-1}.

LCMS Method: Col3_MeCN_FAST_5%-35%

^{1}H NMR

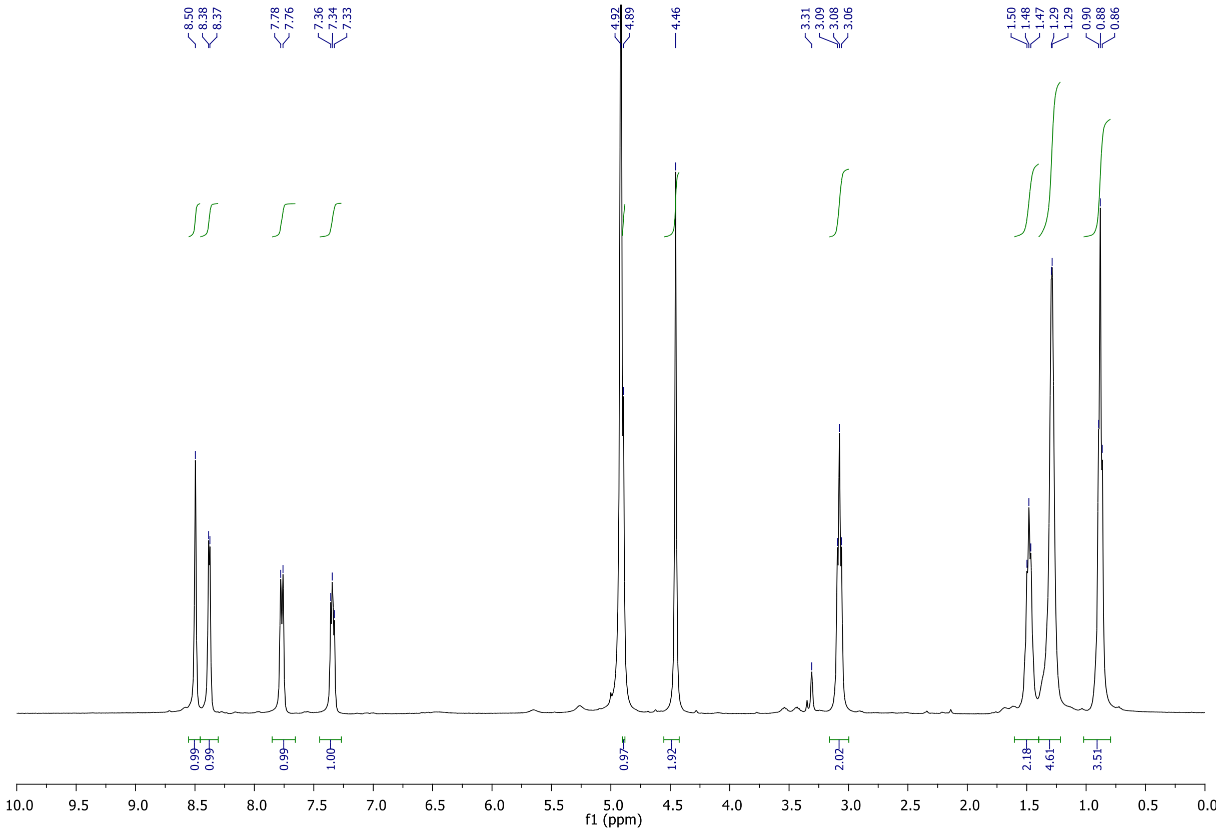

^{13}C NMR

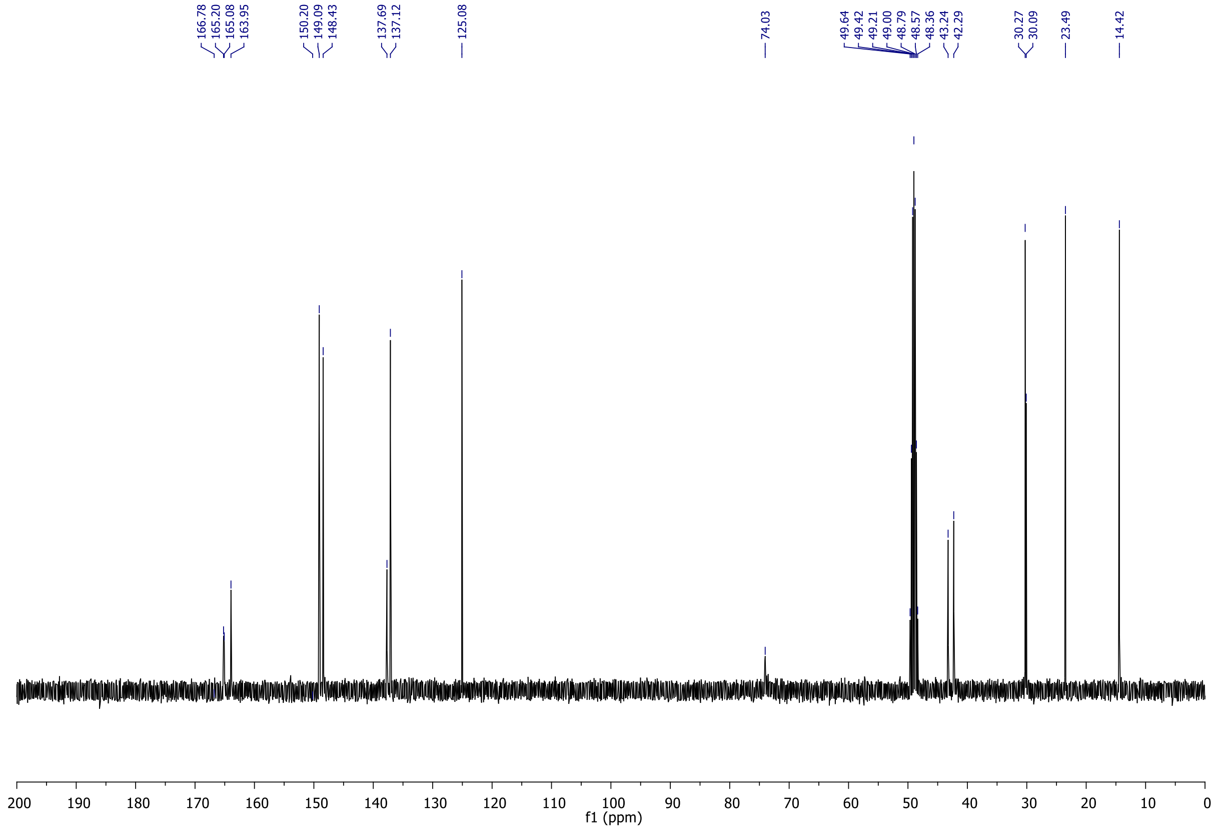

Synthesis of mA2

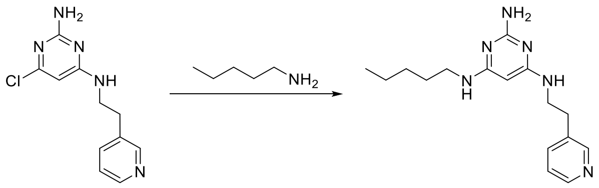

2 (123 mg, 0.493 mmol) was flushed with nitrogen in a MW vial. *n*-Pentylamine (1 mL, 0.9 g, 10 mmol) was added and the mixture was heated in microwave at 150 °C for 7 hours. A saturated solution of $NaHCO_3$ (10 mL) was added and the mixture was extracted with dichloromethane (4 × 30 mL). The organic phase was dried over $MgSO_4$, evaporated and loaded to Celite. A combiflash of the residue on silica (CH_2Cl_2/MeOH: MeOH 0%→10%) provided the title compound (124 mg, 84% yield) as orange dense oil.

^{1}H NMR (CD_3OD, 400 MHz, 298 K): δ 8.41 (s, 1H), 8.36 (d, J = 4.0 Hz, 1H), 7.71 (d, J = 7.5 Hz, 1H), 7.33 (dd, J = 7.5, 5.0 Hz, 1H), 3.45 (t, J = 7.0 Hz, 2H), 3.12 (t, J = 7.0 Hz, 2H), 2.88 (t, J = 7.0 Hz, 2H), 1.56 – 1.53 (m, 2H), 1.35 – 1.30 (m, 4H), 0.91 (t, J = 6.0 Hz, 3H).
^{13}C NMR (CD_3OD, 101 MHz, 298 K): δ 165.1, 165.0, 163.9, 150.4, 147.9, 138.7, 137.4, 125.1, 73.6, 43.2, 42.3, 33.8, 30.3, 30.2, 23.5, 14.4.

HR-MS (ESI): Calculated for $C_{16}H_{25}N_6$ $[M+H]^+$ 301.2141, found: 301.2142 (Δ = 0.3 ppm)

FT-IR (thin film): 3310, 3189, 2953, 2928, 2858, 1573, 1526, 1458, 1429, 1358 cm^{-1}.

LCMS Method: Col3_MeCN_FAST_5%-35%

^{1}H NMR

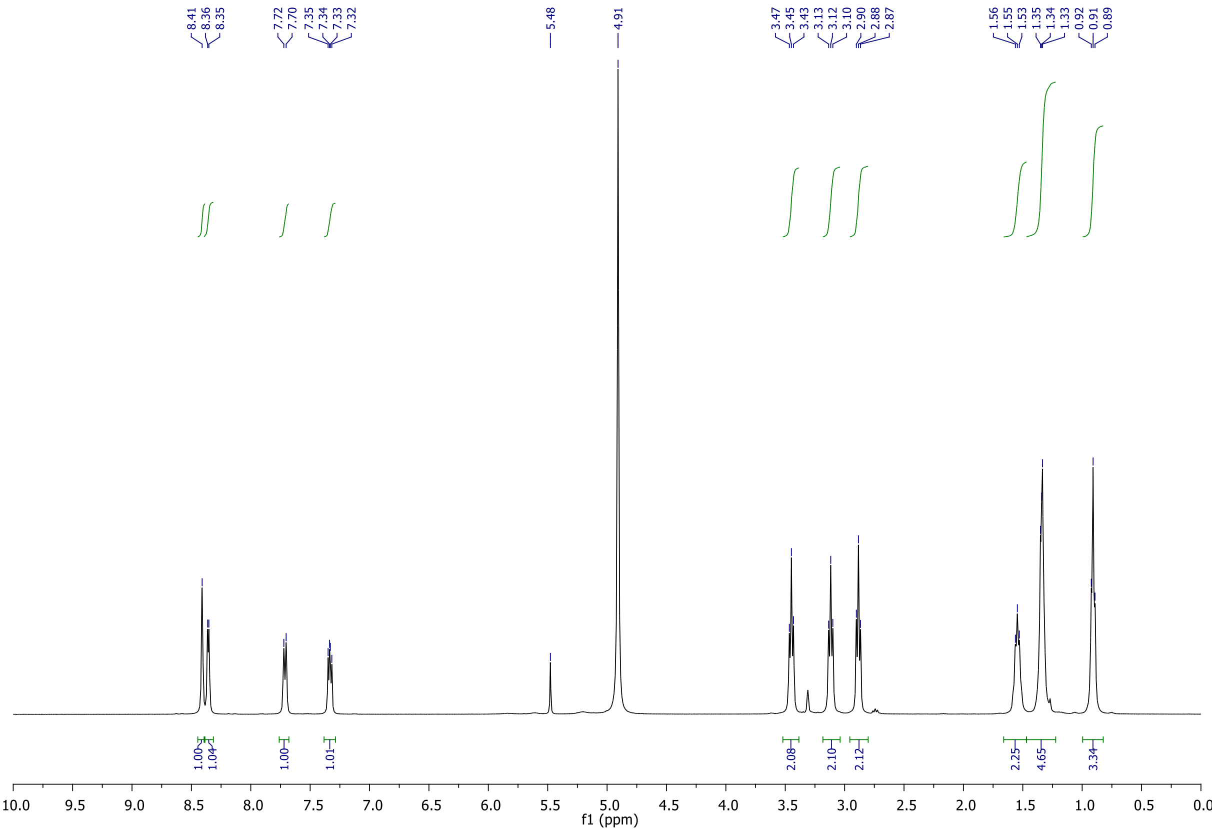

^{13}C NMR

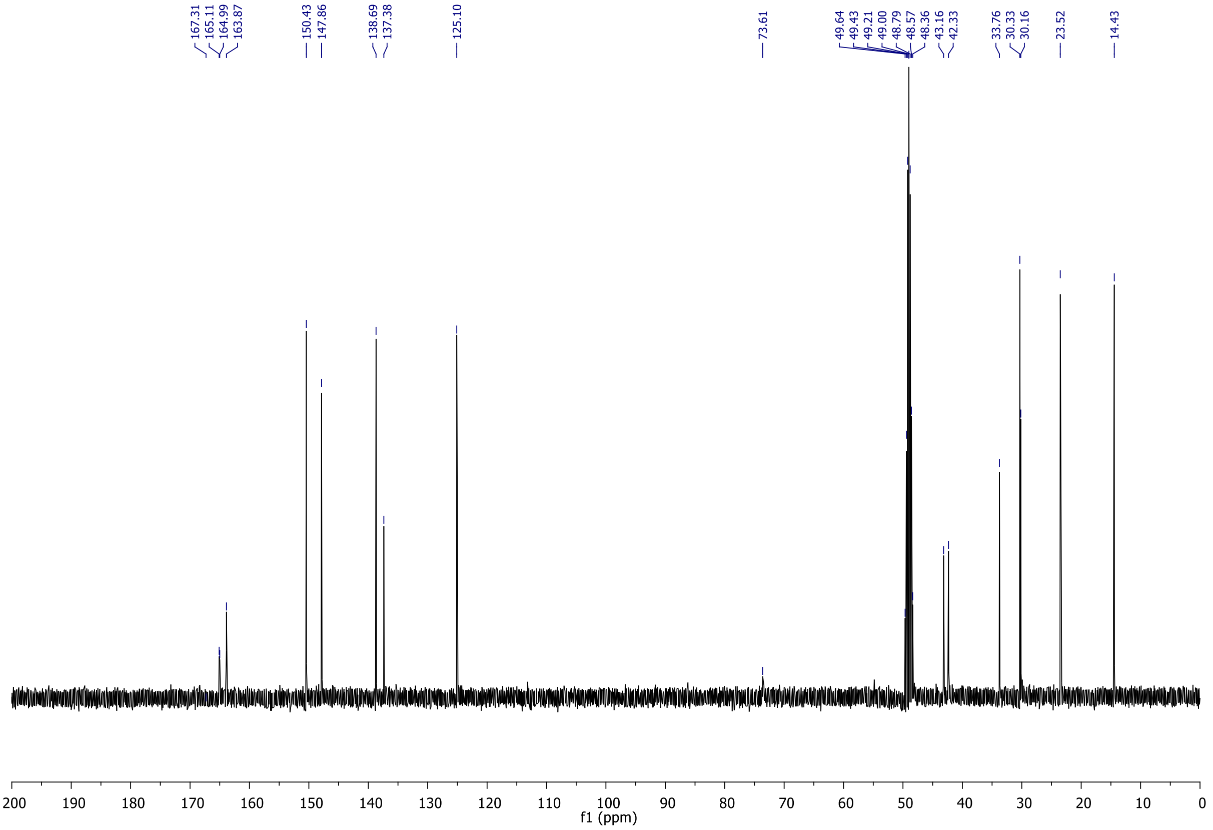

Synthesis of pA1

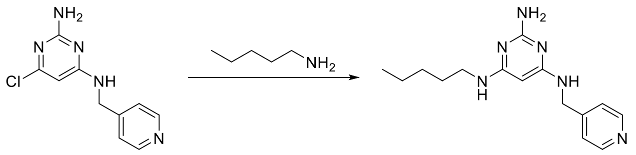

3 (105 mg, 0.45 mmol) was flushed with nitrogen in a MW vial. *n*-Pentylamine (1 mL, 0.9 g, 10 mmol) was added and the mixture was heated in microwave at 160 °C for 3 hours. A saturated solution of $NaHCO_3$ (15 mL) was added and the mixture was extracted with CH_2Cl_2/MeOH (20:1, 2 × 50 mL) and CH_2Cl_2 (2 × 50 mL). The organic phase was dried over $MgSO_4$, evaporated and loaded to Celite. A combiflash of the residue on silica (CH_2Cl_2/MeOH: MeOH 0%→20%) provided the title compound (71 mg, 55% yield) as orange dense oil.

^{1}H NMR (CD_3OD, 400 MHz, 298 K): δ 8.42 (d, *J* = 5.5 Hz, 2H), 7.35 (d, *J* = 5.0 Hz, 2H), 4.86 (s, 1H), 4.47 (s, 2H), 3.08 (t, *J* = 7.0 Hz, 2H), 1.52 – 1.43 (m, 2H), 1.29 (d, *J* = 3.0 Hz, 4H), 0.88 (t, *J* = 6.5 Hz, 3H).
^{13}C NMR (CD_3OD, 101 MHz, 298 K): δ 165.2, 165.2, 163.9, 152.3, 149.9, 123.6, 74.0, 44.7, 42.3, 30.3, 30.1, 23.5, 14.4.

HR-MS (ESI): Calculated for $C_{15}H_{23}N_6$ $[M+H]^+$ 287.1979, found: 287.1975 (Δ = 1.26 ppm)

FT-IR (thin film): 3302, 3175, 2953, 2926, 2587, 1572, 1497, 1446, 1355, 1312, 1219, 1204 cm^{-1}.

LCMS Method: Col3_MeCN_FAST_5%-35%

^{1}H NMR

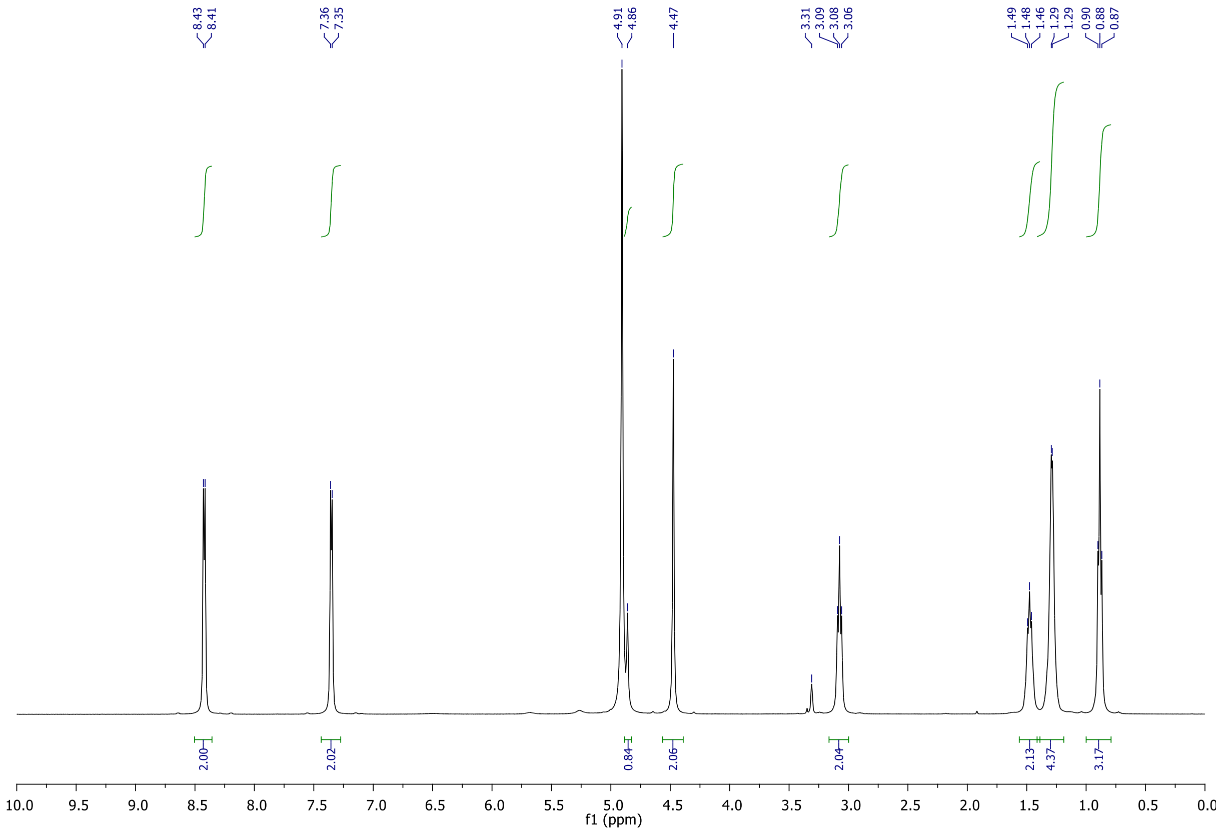

^{13}C NMR

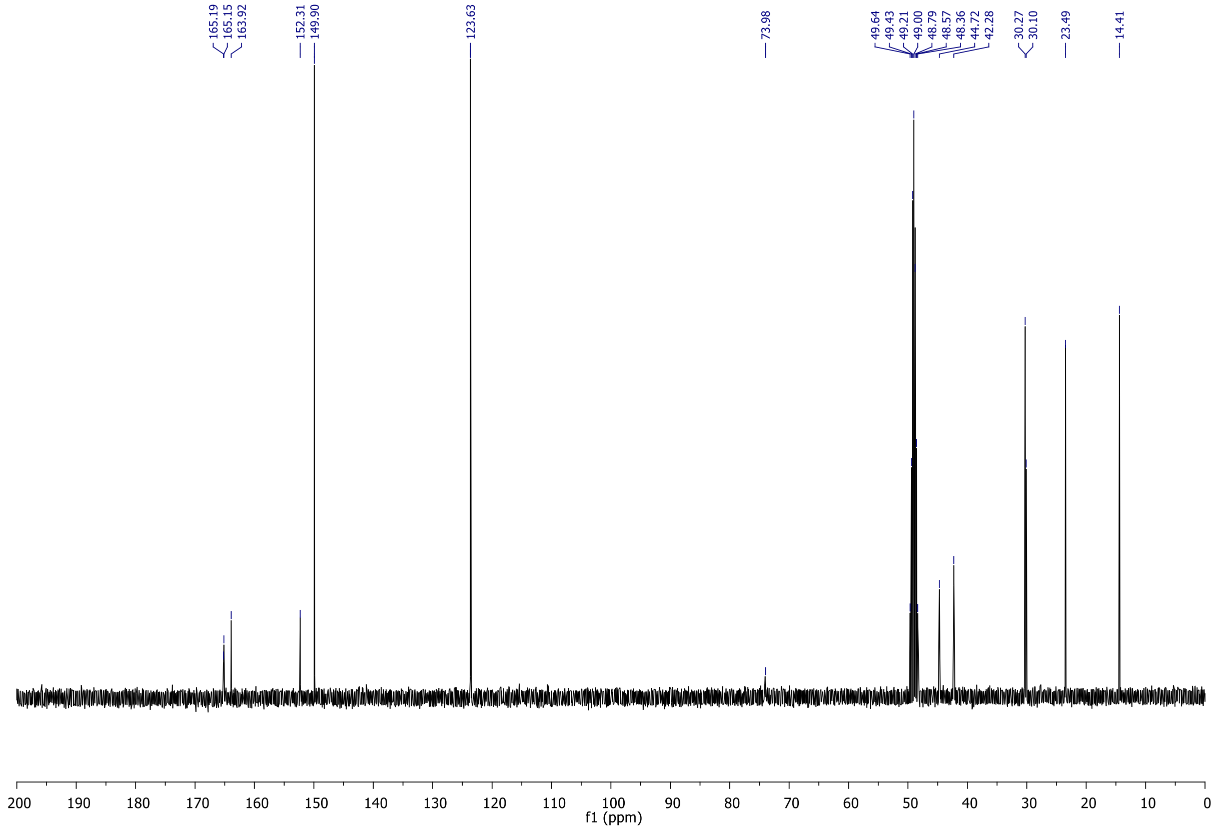

Synthesis of pA2

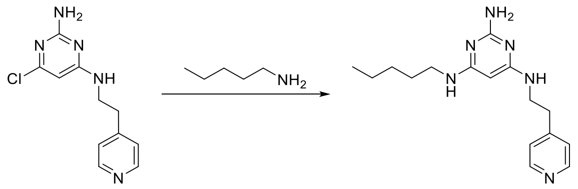

4 (100 mg, 0.40 mmol) was flushed with nitrogen in a MW vial. *n*-Pentylamine (1 mL, 0.9 g, 10 mmol) was added and the mixture was heated in microwave at 160 °C for 5 hours. A saturated solution of $NaHCO_3$ (10 mL) and water (10 mL) were added and the mixture was extracted with CH_2Cl_2/MeOH (20:1, 50 mL) and CH_2Cl_2 (3 × 50 mL). The organic phase was dried over $MgSO_4$, evaporated and loaded to Celite. A combiflash of the residue on silica (CH_2Cl_2/MeOH: MeOH 0%→10%) provided the title compound (97 mg, 81% yield) as yellow oil.

^{1}H NMR ($CDCl_3$, 400 MHz, 298 K): δ 8.53 (d, J = 6.0 Hz, 2H), 7.15 (d, J = 6.0 Hz, 2H), 4.80 (s, 1H), 4.60 – 4.41 (m, 3H), 3.51 (dd, J = 13.0, 7.0 Hz, 2H), 3.13 (dd, J = 13.0, 7.0 Hz, 2H), 2.89 (t, J = 7.0 Hz, 2H), 1.61 – 1.54 (m, 2H), 1.39 – 1.30 (m, 4H), 0.90 (t, J = 7.0 Hz, 3H).
^{13}C NMR ($CDCl_3$, 101 MHz, 298 K): δ 164.1, 163.8, 162.2, 150.0, 148.0, 124.1, 72.5, 41.7, 41.7, 35.1, 29.1, 29.0, 22.4, 14.0.

HR-MS (ESI): Calculated for $C_{16}H_{25}N_6$ $[M+H]^+$ 301.2135, found: 301.2131 (Δ = 1.30 ppm)

FT-IR (thin film): 3310, 2954, 2927, 2857, 2421, 1555, 1494, 1455, 1430, 1417, 1357, 1219 cm^{-1}.

LCMS Method: Col3_MeCN_FAST_5%-35%

^{1}H NMR

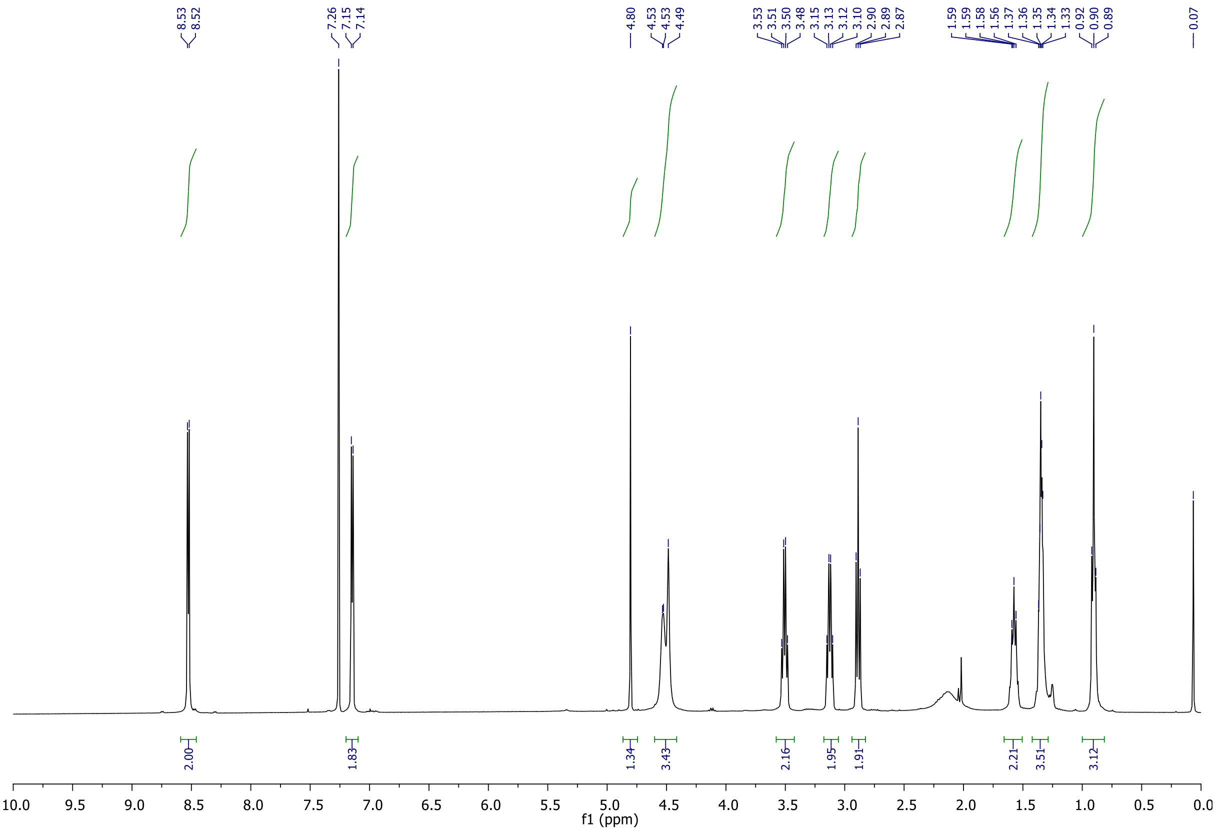

^{13}C NMR

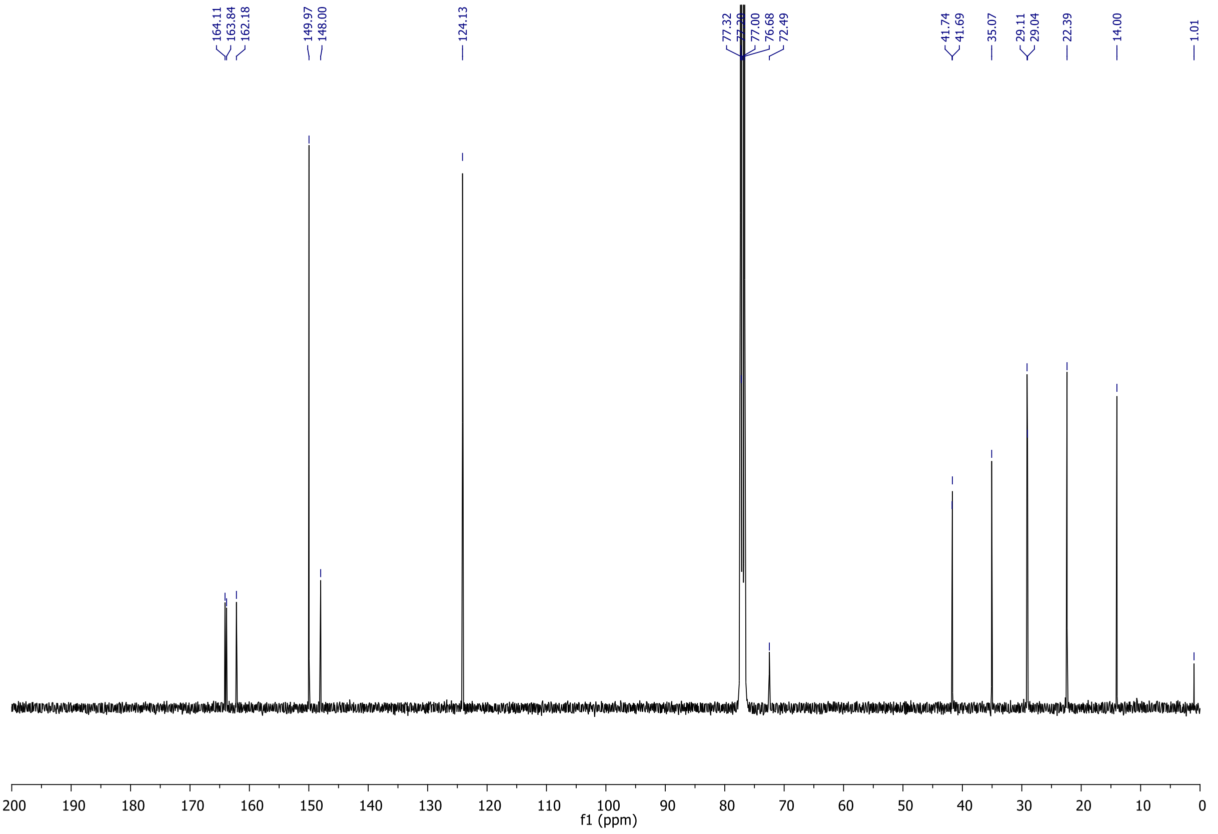

Synthesis of 5

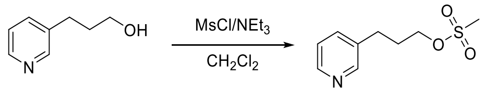

To a solution of 3-(pyridin-3-yl)propan-1-ol (1 mL, 1.06 g, 7.7 mmol) and NEt_3 (3 mL) in CH_2Cl_2 (50 mL) was added methanesulfonyl chloride (0.8 mL, 10.3 mmol, 1.4 eq) at 0 °C in small parts over the period of 90 minutes. The reaction mixture was stirred at 0 °C for additional 30 minutes and then diluted with CH_2Cl_2 (50 mL). The still-cool solution was washed with H_2O (2×30 mL) and brine (30 mL), dried with $MgSO_4$ and solvents were removed under reducer pressure (at no more than 30 °C). A combiflash of the residue on silica gel (PE/EtOAc, EtOAc: 0%→100%) provided the title compound as yellowish liquid (1.65 g; quant.)
(*This compound is quite unstable even when stored in fridge.)*

^{1}H NMR ($CDCl_3$, 400 MHz, 298 K): δ 8.53 – 8.42 (m, 2H), 7.52 (dt, *J* = 8.0 Hz, 2.0 Hz, 1H), 7.23 (dd, J = 8.0, 5.0 Hz, 1H), 4.24 (t, *J* = 6.0 Hz, 2H), 3.01 (s, 3H), 2.80 – 2.74 (t, *J* = 7.0 Hz, 2H), 2.13 – 2.05 (m, 2H).
^{13}C NMR ($CDCl_3$, 101 MHz, 298 K): 149.9, 147.9, 135.9, 135.6, 123.4, 68.6, 37.4, 30.4, 28.8.

^{1}H NMR

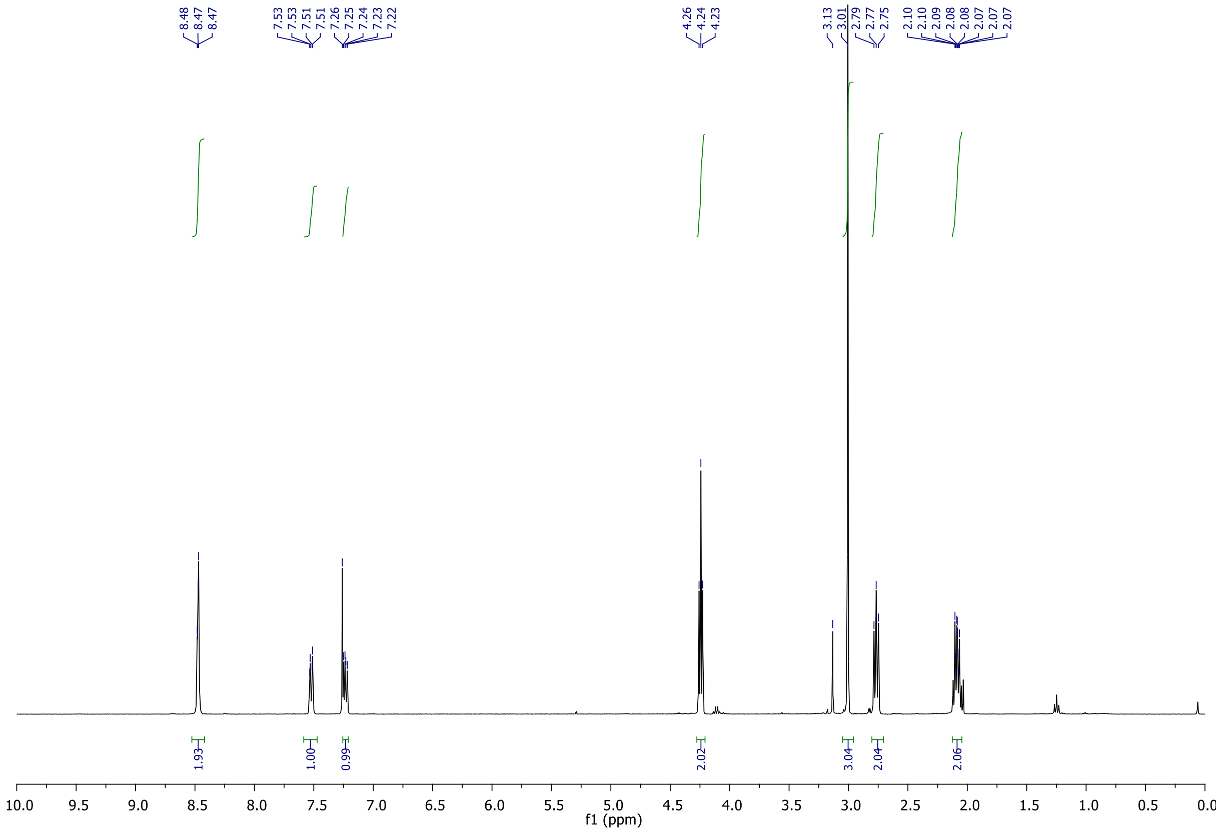

^{13}C NMR

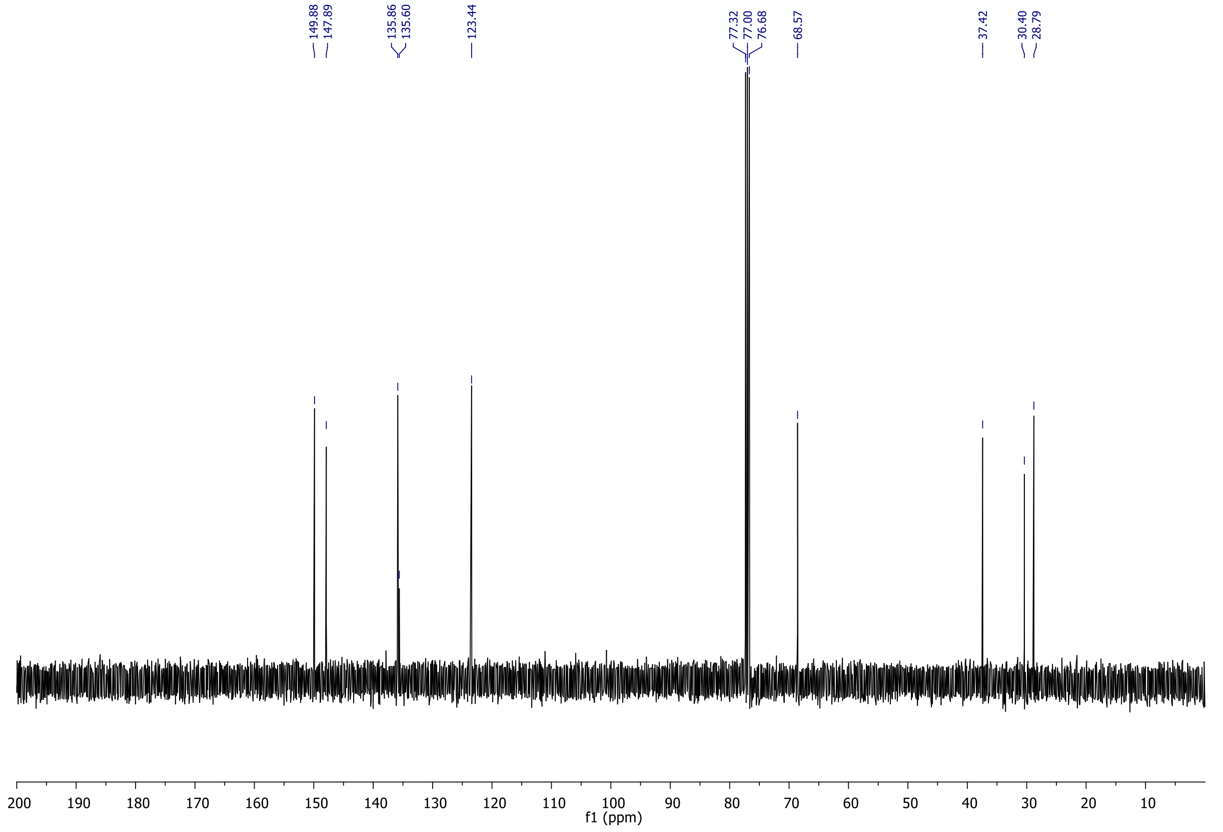

Synthesis of 5-BH$_3$

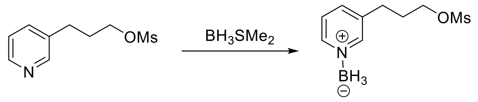

5 (2.41 g, 11.1 mmol) was flushed with nitrogen. Then THF (20 mL) was added, which produced slurry solution. The reaction mixture was cooled to –78 °C. To this solution, BH_3•SMe_2 (6.0 mL, 2.0M solution in THF, 12 mmol) was added dropwise at –78 °C. After 30 minutes of stirring, the cooling bath was removed. After stirring at RT for 1 hour, the conversion was checked by TLC (10:1 EtOAc/MeOH) and additional BH_3•SMe_2 (1.0 mL, 2.0M solution in THF, 2 mmol; altogether 14 mmol) was added dropwise at –78 °C. The solution was stirred for 15 minutes and the cooling bath was removed. After additional 30 minutes of stirring, the mixture was cooled again to –78 °C and methanol (9 mL) was added dropwise. After 15 minutes, the cooling bath was removed and the mixture was allowed to heat to room temperature and then solvents were removed under reduced pressure. A combiflash of the residue on silica (Celite loading, PE/EtOAc; EtOAc: 0%→100%) provided the title compound (2.25 g, 88% yield) as transparent oil, which crystalise upon standing in a freezer over a couple of days/weeks to form a white solid.

^{1}H NMR (CDCl$_3$, 400 MHz, 298 K): δ 8.45 – 8.43 (m, 2H), 7.78 (d, J = 8.0 Hz, 1H), 7.45 (dd, J = 8.0, 6.0 Hz, 1H), 4.25 (td, J = 6.0, 1.0 Hz, 2H), 3.02 (d, J = 1.0 Hz, 3H), 2.84 (t, J = 8.0 Hz, 2H), 2.75 – 2.15 (br, 3H), 2.16 – 2.02 (m, 2H).
^{1}H NMR (CDCl$_3$, 400 MHz, 298 K, ^{11}B decoupled): δ 8.45 – 8.43 (m, 2H), 7.78 (d, J = 8.0 Hz, 1H), 7.45 (dd, J = 8.0, 6.0 Hz, 1H), 4.24 (t, J = 6.0 Hz, 2H), 3.02 (d, J = 1 Hz, 3H), 2.83 (t, J = 8.0 Hz, 2H), 2.55 (s, 3H), 2.16 – 2.02 (m, 2H).
^{13}C NMR (CDCl$_3$, 101 MHz, 298 K): δ 147.2, 145.4, 139.2, 138.5, 125.2, 68.11, 37.4, 29.9, 28.6.
^{11}B NMR (CDCl$_3$, 128 MHz, 298 K, normal glass): δ –12.4 (d, J = 88 Hz)

HR-MS (ESI): Calculated for $C_9H_{15}BNO_3S$ [M–H]$^+$ 228.0866, found: 228.0865 (Δ = 0.4 ppm)

FT-IR (thin film): 2936, 2360, 2310, 2271, 1620, 1585, 1486, 1443, 1346, 1331, 1164.

MP: 51 – 53 °C

LCMS Method: Col1-MeCN-FAST

^{1}H NMR

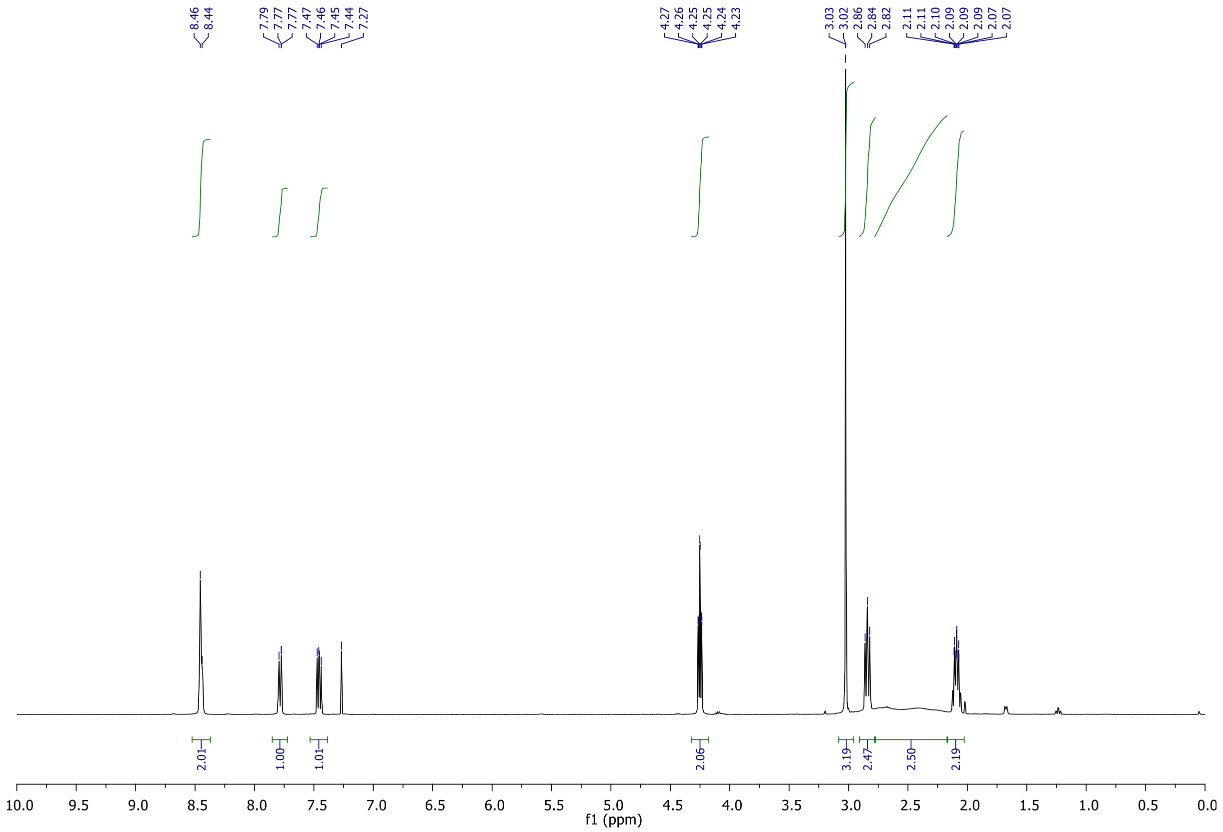

^{1}H NMR (^{11}B decoupled)

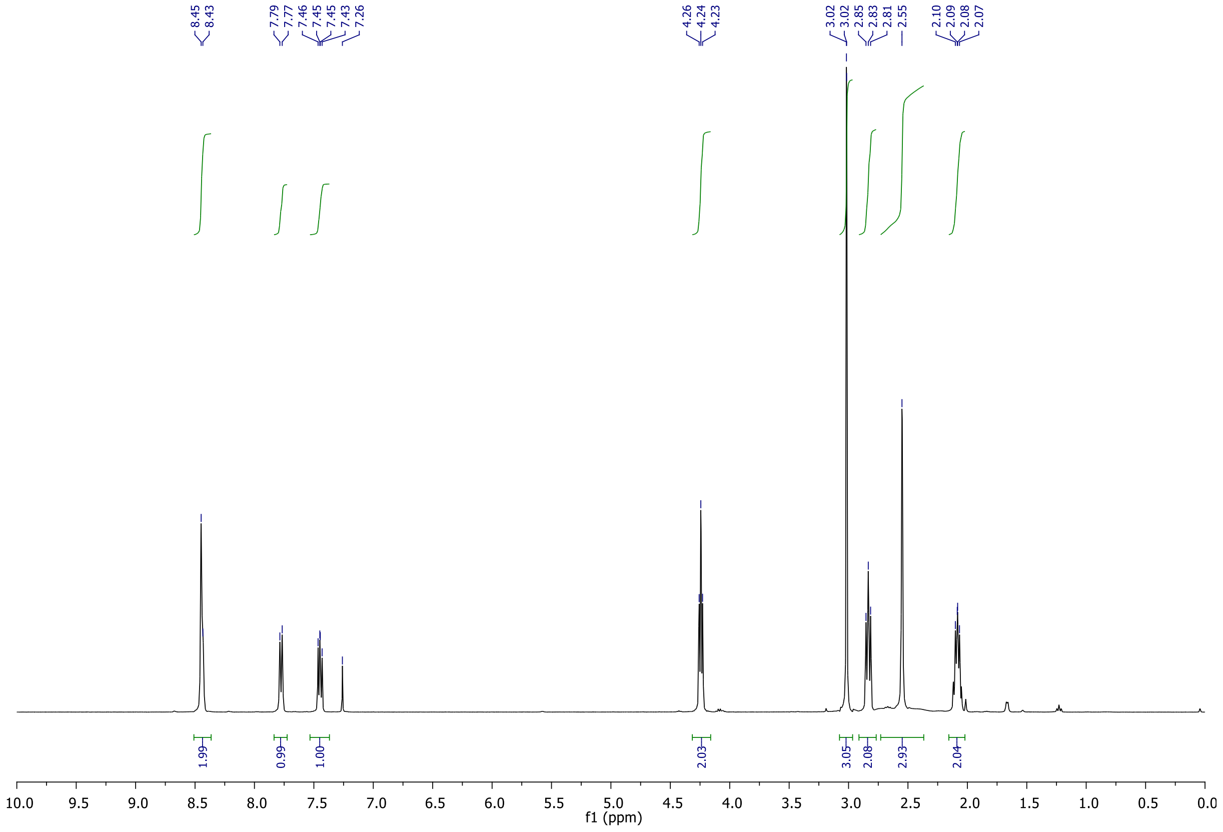

^{13}C NMR

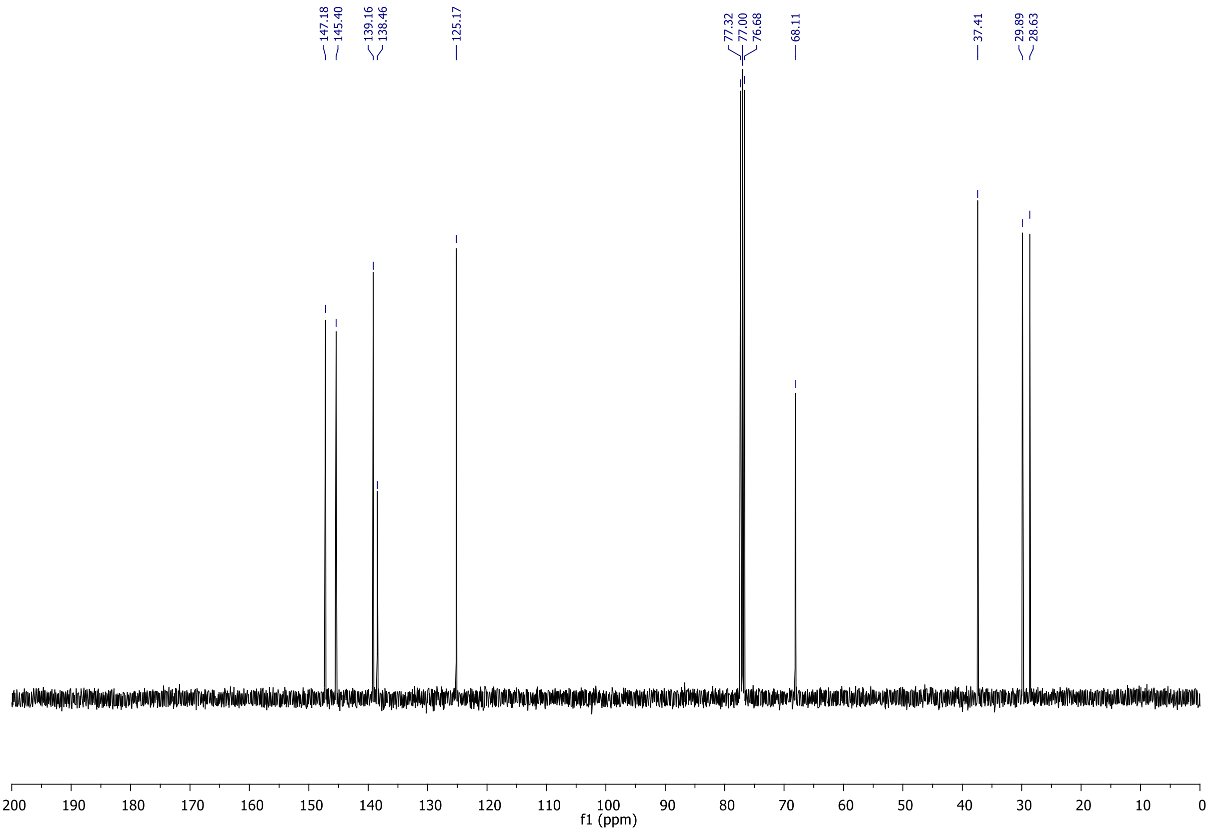

^{11}B NMR (normal glass)

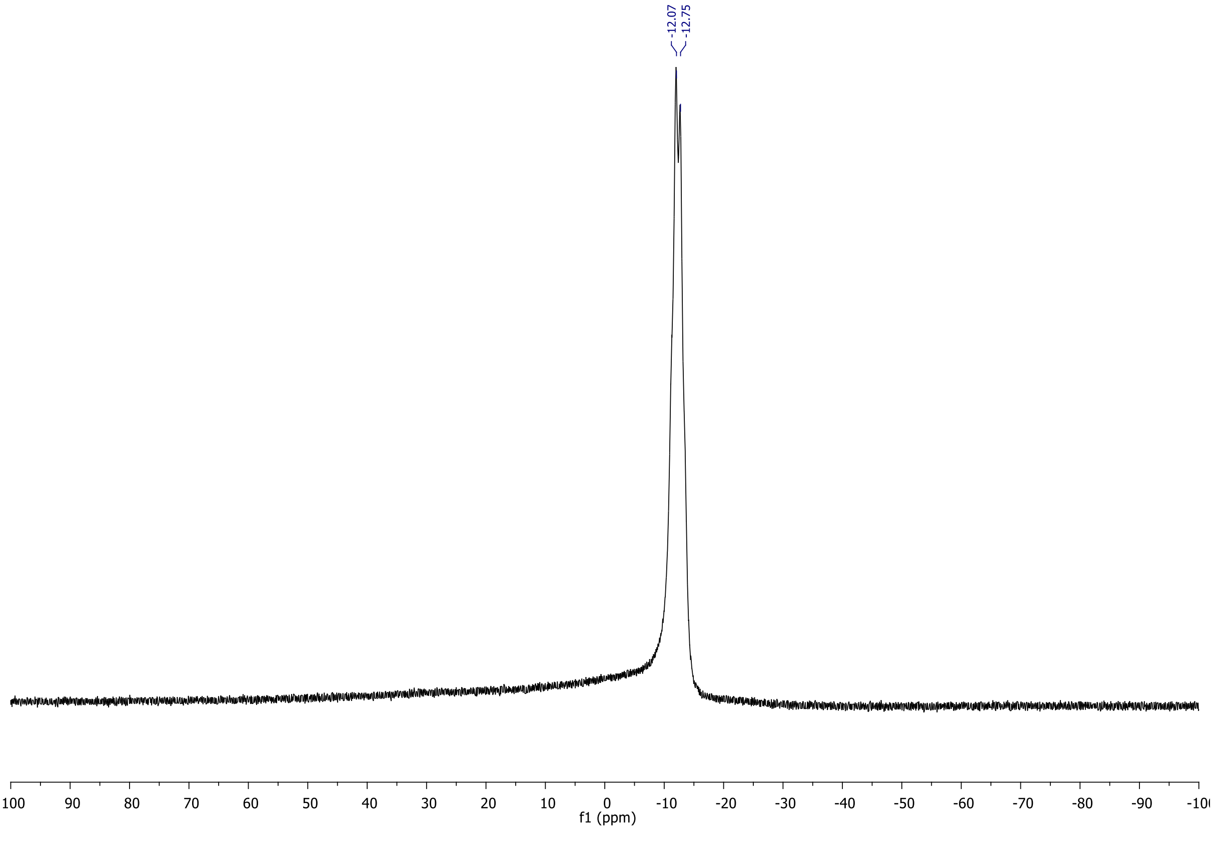

Synthesis of 6-BH$_3$ and 6

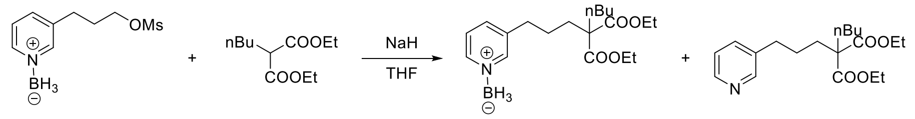

NaH (60% in mineral oil, 255 mg, 6.38 mmol) was flushed with nitrogen. THF (10 mL) was added and the mixture was cooled to 0 °C. Then diethyl-2-butylmalonate (1.45 mL, 6.52 mmol) was added dropwise (the solution became clear after the addition). **5-BH$_3$** (718 mg, 3.13 mmol) was added as a solid. After 1 hour of stirring, the cooling bath was removed. The mixture was heated at 60 °C for 4 hours and after cooling to RT, water (20 mL) was added and the mixture was extracted with dichloromethane (4 × 30 mL). The organic phase was dried over $MgSO_4$ and the solvents were removed under reduced pressure. A combiflash of the residue on silica (Celite loading, PE/EtOAc, EtOAc: 0%→50%) provided after drying in vacuum oven overnight products **6-BH$_3$** (655 mg, 60% yield) and **6** (219 mg, 21% yield) as transparent dense liquids (**6-BH$_3$** crystalise upon storage in freezer over a couple of weeks).

LCMS Method: Col1-MeCN-SLOW

Compound **6-BH$_3$**:
^{1}H NMR ($CDCl_3$, 400 MHz, 298 K): δ 8.42 – 8.40 (m, 2H), 7.70 (d, J = 8.0 Hz, 1H), 7.40 (dd, J = 8.0, 6.0 Hz, 1H), 4.21 – 4.06 (m, 4H), 3.00 – 2.11 (br, 3H), 2.66 (t, J = 8.0 Hz, 2H), 1.91 – 1.78 (m, 4H), 1.59 – 1.46 (m, 2H), 1.32 – 1.23 (m, 2H), 1.20 (t, J = 7.0 Hz, 6H), 1.11 – 1.03 (m, 3H), 0.85 (t, J = 7.0 Hz, 3H).
^{1}H NMR ($CDCl_3$, 400 MHz, 298 K, ^{11}B decoupled): δ 8.42 – 8.40 (m, 2H), 7.70 (d, J = 8.0 Hz, 1H), 7.40 (dd, J = 8.0, 6.0 Hz, 1H), 4.21 – 4.08 (m, 4H), 2.66 (t, J = 8.0 Hz, 2H), 2.55 (s, 3H), 1.92 – 1.77 (m, 4H), 1.60 – 1.45 (m, 2H), 1.34 – 1.23 (m, 2H), 1.20 (t, J = 7.0 Hz, 6H), 1.15 – 1.01 (m, 2H), 0.86 (t, J = 7.0 Hz, 3H).
^{13}C NMR ($CDCl_3$, 101 MHz, 298 K): δ 171.4, 147.1, 145.1, 139.6, 138.8, 124.9, 61.1, 57.2, 32.8, 32.2, 31.7, 26.1, 25.1, 22.8, 14.00, 13.8.
^{11}B NMR ($CDCl_3$, 128 MHz, 298 K, normal glass): δ **–**12.4 (d, J = 79 Hz)

HR-MS: Calculated for $C_{21}H_{34}BN_2O_4$ [M+CH_3CN–H]$^+$ 389.2612, found: 389.2627 (Δ = 3.9 ppm)

FT-IR (thin film): 2959, 2935, 2872, 2371, 1725, 1619, 1484, 1463, 1444 cm^{-1}.

MP: 37 – 40 °C

Compound **6**:
^{1}H NMR ($CDCl_3$, 400 MHz, 298 K): δ 8.38 – 8.36 (m, 2H), 7.41 (d, J = 8.0 Hz, 1H), 7.13 (dd, J = 8.0, 5.0 Hz, 1H), 4.09 (q, J = 7.0 Hz, 4H), 2.56 (t, J = 7.5 Hz, 2H), 1.91 – 1.71 (m, 4H), 1.55 – 1.37 (m, 2H), 1.28 – 1.17 (m, 2H), 1.14 (t, J = 7.0 Hz, 6H), 1.06 – 0.98 (m, 2H), 0.81 (t, J = 7.5 Hz, 3H).
^{13}C NMR ($CDCl_3$, 101 MHz, 298 K): δ 171.5, 149.8, 147.3, 136.8, 135.6, 123.1, 60.9, 57.2, 32.9, 31.9, 31.5, 25.9, 25.4, 22.7, 13.9, 13.7.

HR-MS: Calculated for $C_{19}H_{30}NO_4$ [M+H]$^+$ 336.2175, found: 336.2173 (Δ = 0.6 ppm)

FT-IR (thin film): 2958, 2935, 2872, 1725, 1575, 1478, 1463, 1423 cm^{-1}.

6-BH$_3$: ^{1}H NMR

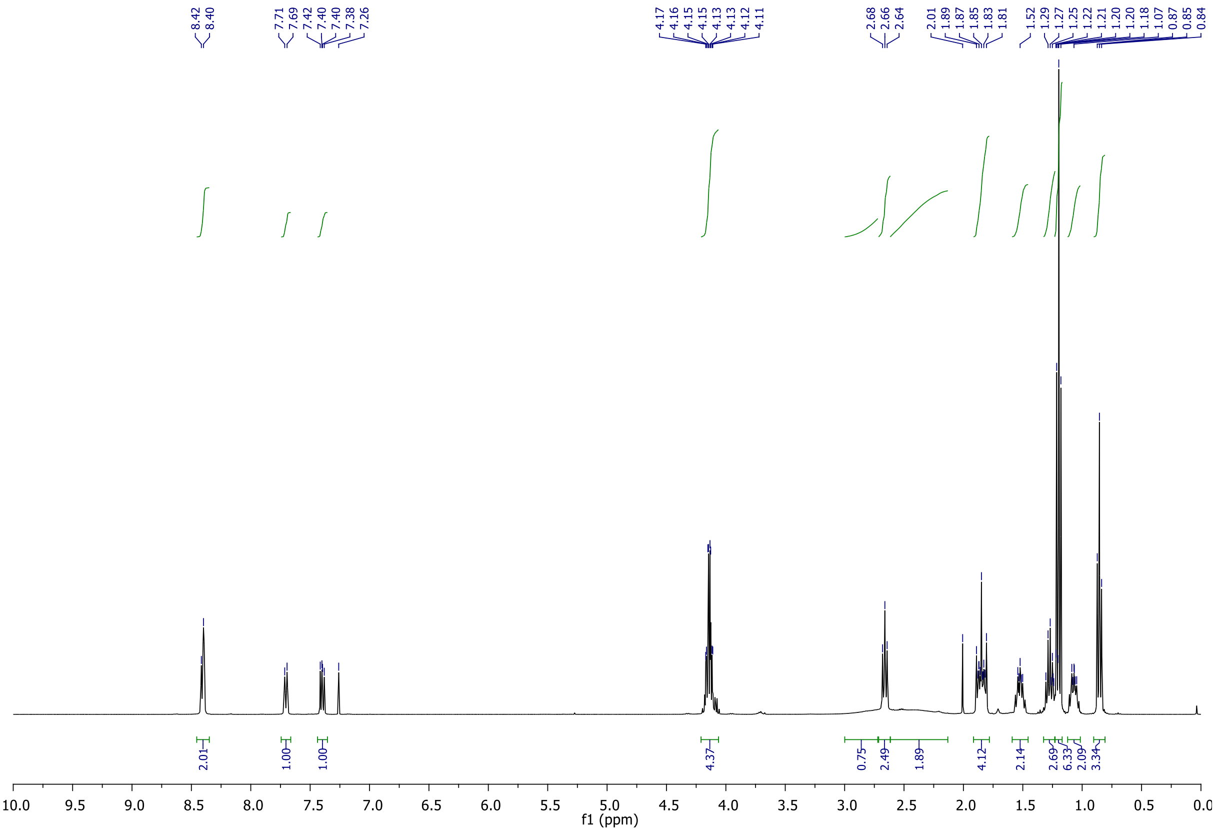

6-BH$_3$: ^{1}H NMR (^{11}B decoupled)

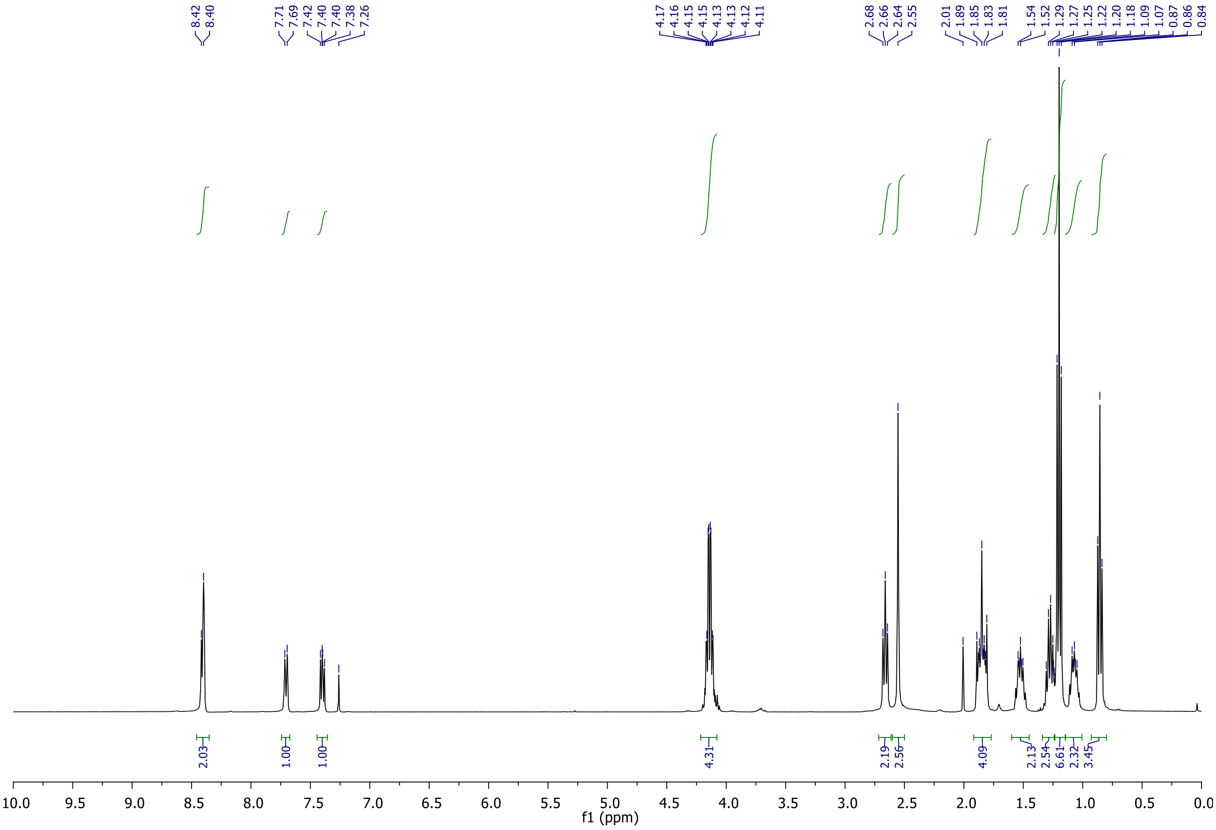

6-BH$_3$: ^{13}C NMR

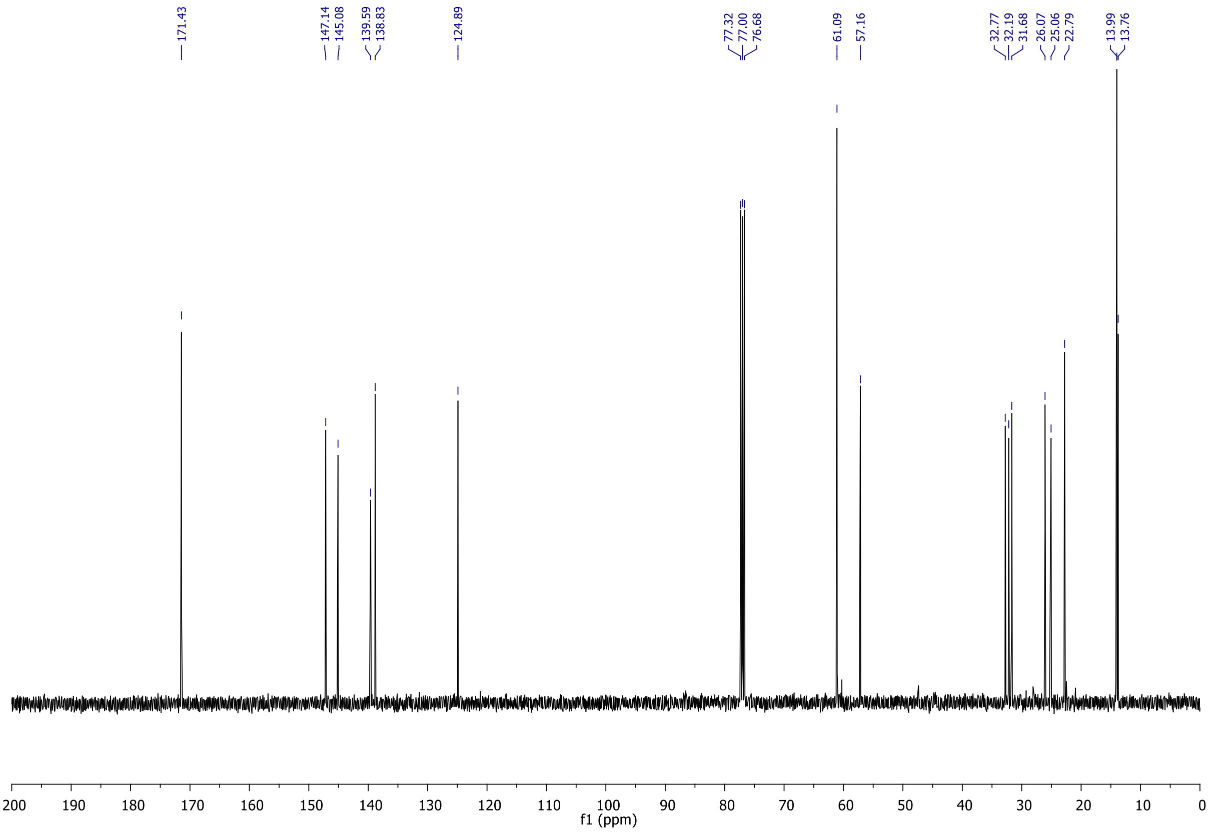

6-BH$_3$: ^{11}B (normal glass)

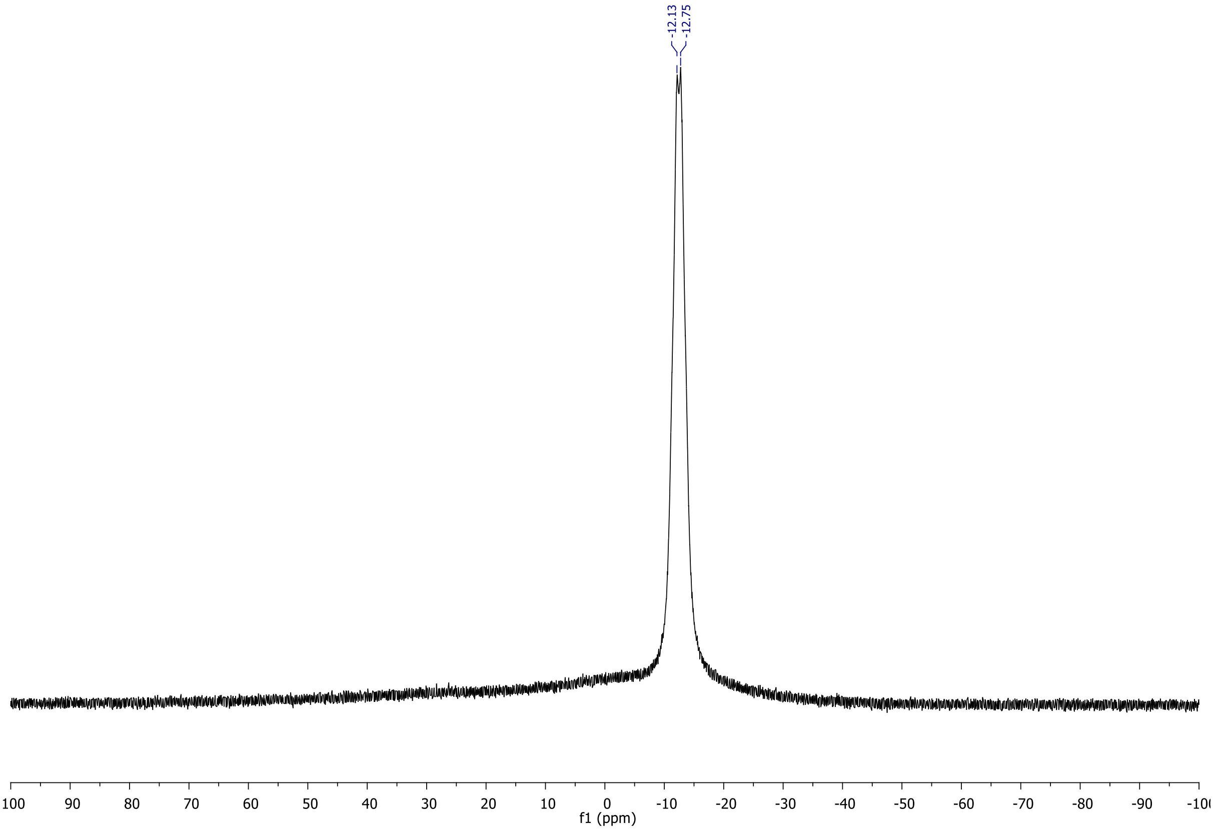

6: ^1H NMR

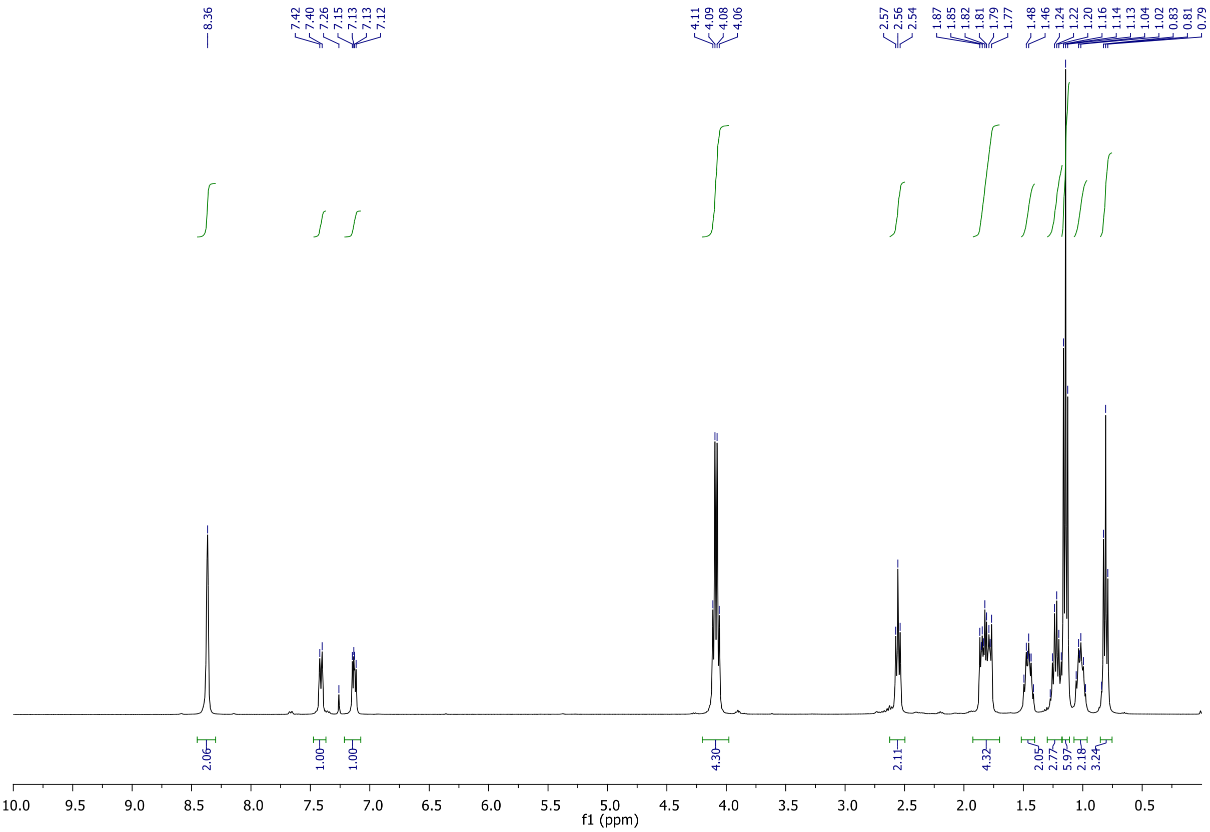

6: ^{13}C NMR

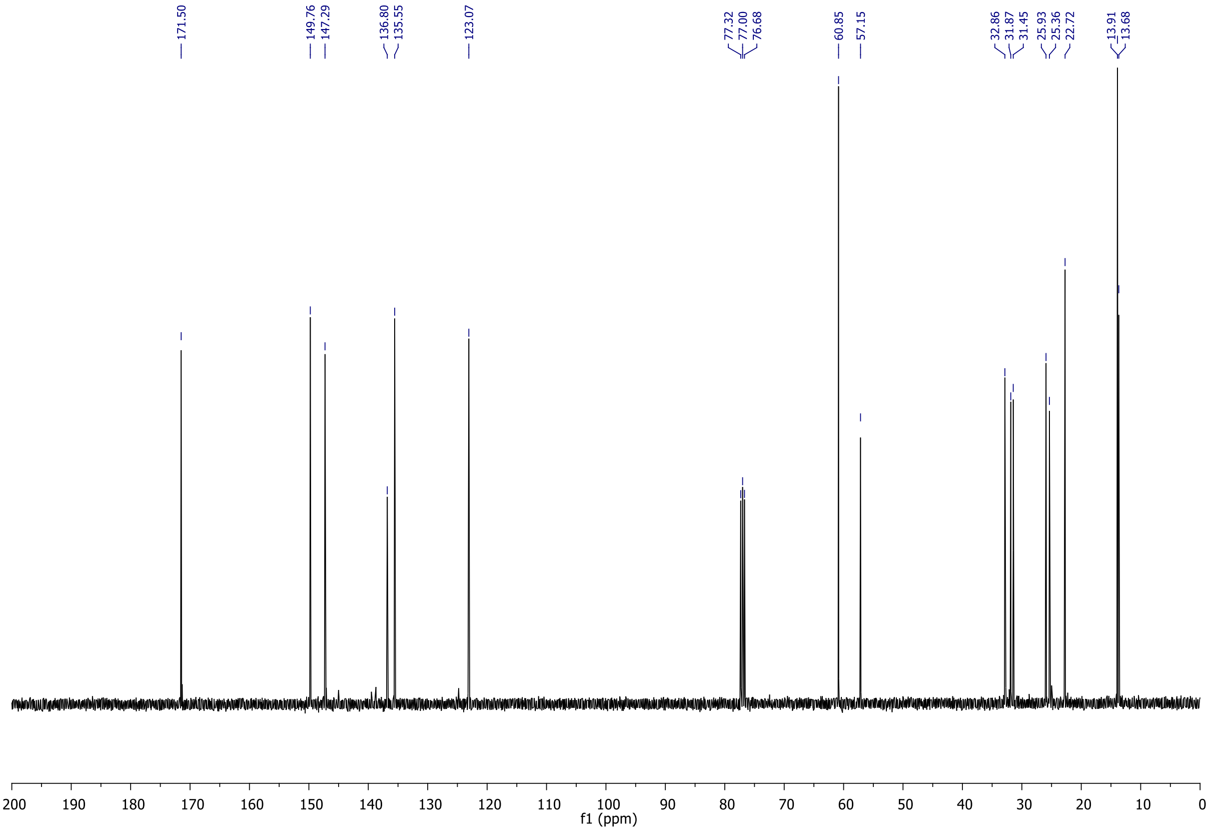

Synthesis of mB3-BH$_3$ and mB3

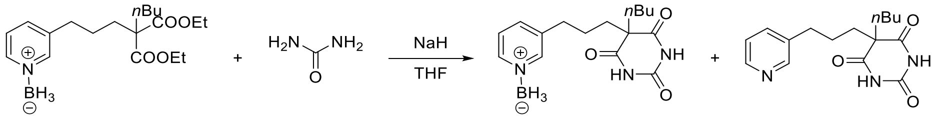

NaH (60% in mineral oil, 300 mg, 7.5 mmol) was flushed with nitrogen and DMF (6 mL) was added. The mixture was cooled to 0 °C and urea (1.18 g, 19.6 mmol) was added. After 2 hours of stirring, **6-BH$_3$** (646 mg, 1.85 mmol) in DMF (7 mL) was added at 0 °C. The mixture was stirred overnight at RT and a saturated solution of NH_4Cl (5 mL) was added with cooling to 0 °C. Water (10 mL) was added and the mixture was extracted with dichloromethane (3 × 40 mL). The collected organic phase was dried over $MgSO_4$ and the solvents were removed under reduced pressure (60 °C, <14 mBar). A combliflash of residue on silica (Celite loading, PE/EtOAc, EtOAc: 0%→100%) provided after drying in vacuum oven overnight products **mB3-BH$_3$** (350 mg, 60% yield) and **mB3** (117 mg, 21% yield) as white crystalline solids.

LCMS Method: Col3-MeCN-FAST

Compound **mB3-BH$_3$**:
^{1}H NMR (CD$_3$OD, 400 MHz, 298 K): δ 8.42 (s, 2H), 7.88 (d, J = 8.0 Hz, 1H), 7.53 (dd, J = 8.0, 6.0 Hz, 1H), 2.95 – 2.09 (br, 3H), 2.69 (t, J = 7.5 Hz, 2H), 1.97 – 1.86 (m, 4H), 1.58 – 1.50 (m, 2H), 1.32 – 1.22 (m, 2H), 1.22 – 1.13 (m, 2H), 0.87 (t, J = 7.0 Hz, 3H).
^{1}H NMR (CD$_3$OD, 400 MHz, 298 K, ^{11}B decoupled): δ 8.42 (s, 2H), 7.88 (d, J = 8.0 Hz, 1H), 7.53 (dd, J = 8.0, 6.0 Hz, 1H), 2.69 (t, J = 7.5 Hz, 2H), 2.54 (s, 3H), 1.97 – 1.86 (m, 4H), 1.58 – 1.51 (m, 2H), 1.32 – 1.22 (m, 2H), 1.22 – 1.13 (m, 2H), 0.87 (t, J = 7.0 Hz, 3H).
^{13}C NMR (CD$_3$OD, 101 MHz, 298 K): δ 174.9, 151.2, 148.4, 146.4, 141.0, 140.6, 126.5, 56.7, 40.1, 38.6, 33.1, 28.1, 27.1, 23.6, 14.0.
^{11}B NMR (CD$_3$OD, 128 MHz, 298 K, normal glass): δ –12.6

HR-MS: Calculated for $C_{16}H_{23}BN_3O_3$ [M–H]$^+$ 316.1832, found: 316.1844 (Δ = 3.8 ppm)

FT-IR (thin film): 3326, 3104, 2959, 2931, 2860, 2368, 1753, 1699, 1619, 1585, 1486, 1441, 1421, 1384, 1335, 1336, 1321 cm^{-1}.

MP: > 161°C (decompose and produce gas)

Compound **mB3**:
This product is characterised below.

mB3-BH$_3$: ^{1}H NMR

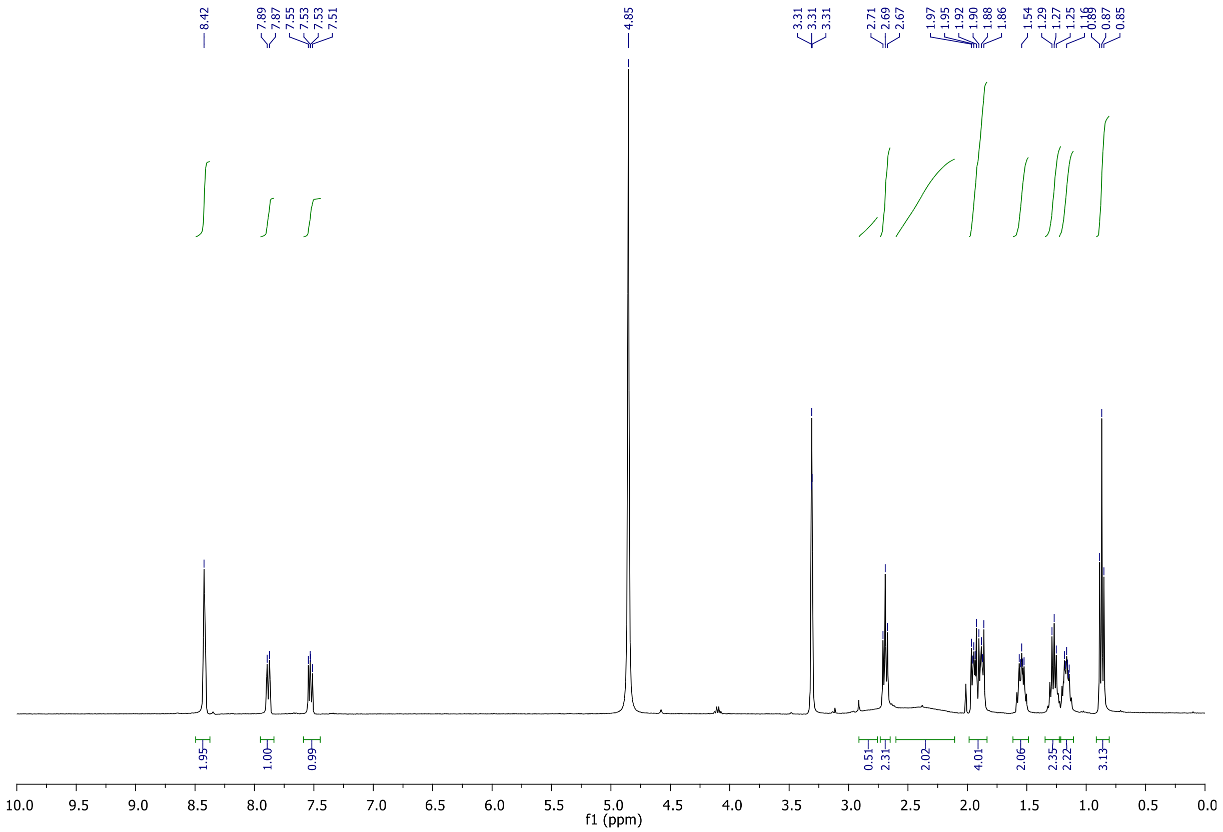

mB3-BH$_3$: ^{1}H NMR (^{11}B decoupled)

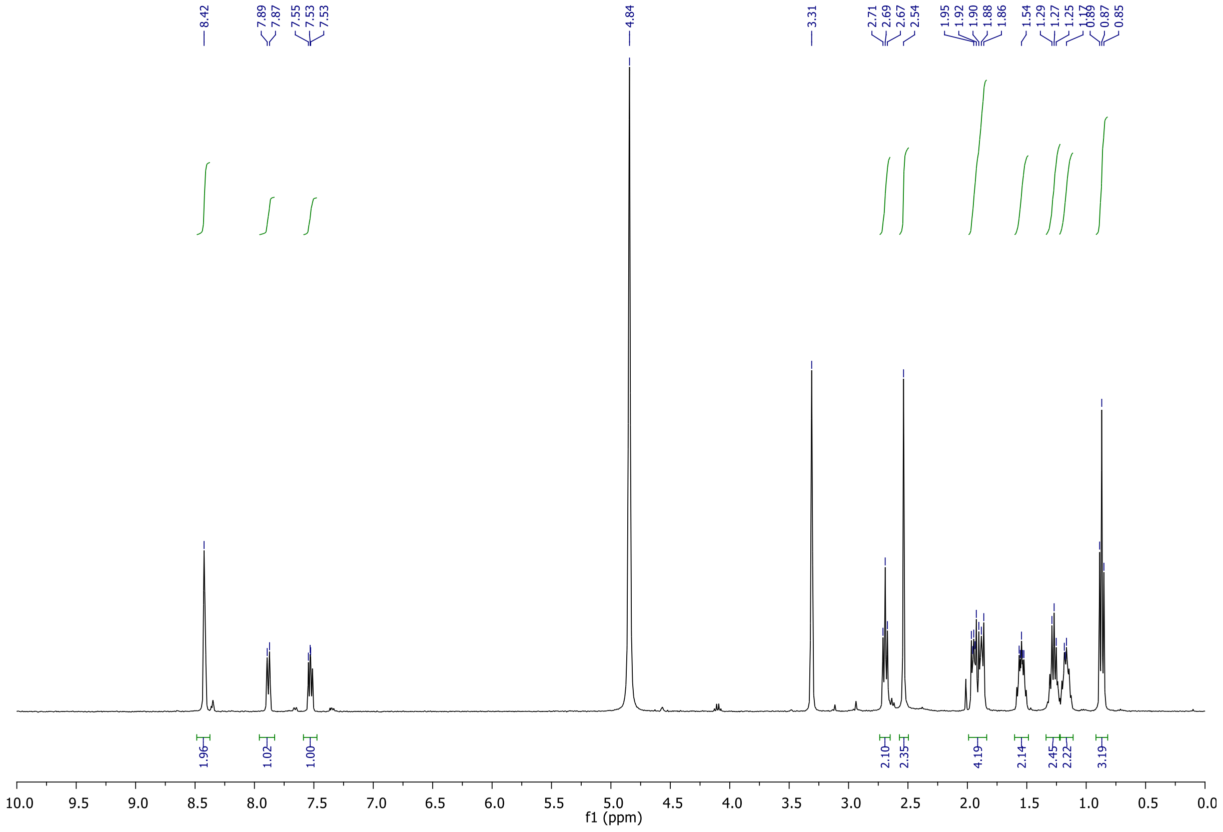

mB3-BH$_3$: ^{13}C NMR

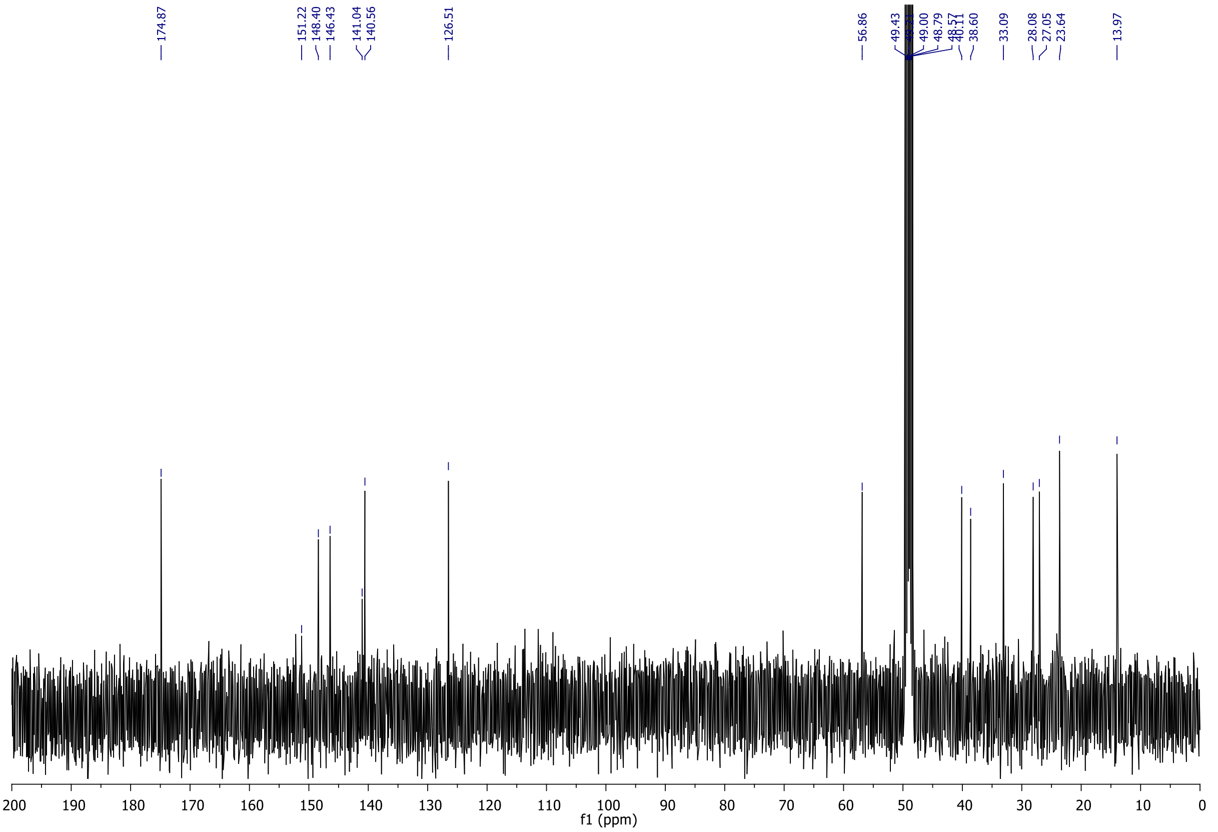

mB3-BH$_3$: ^{11}B (normal glass)

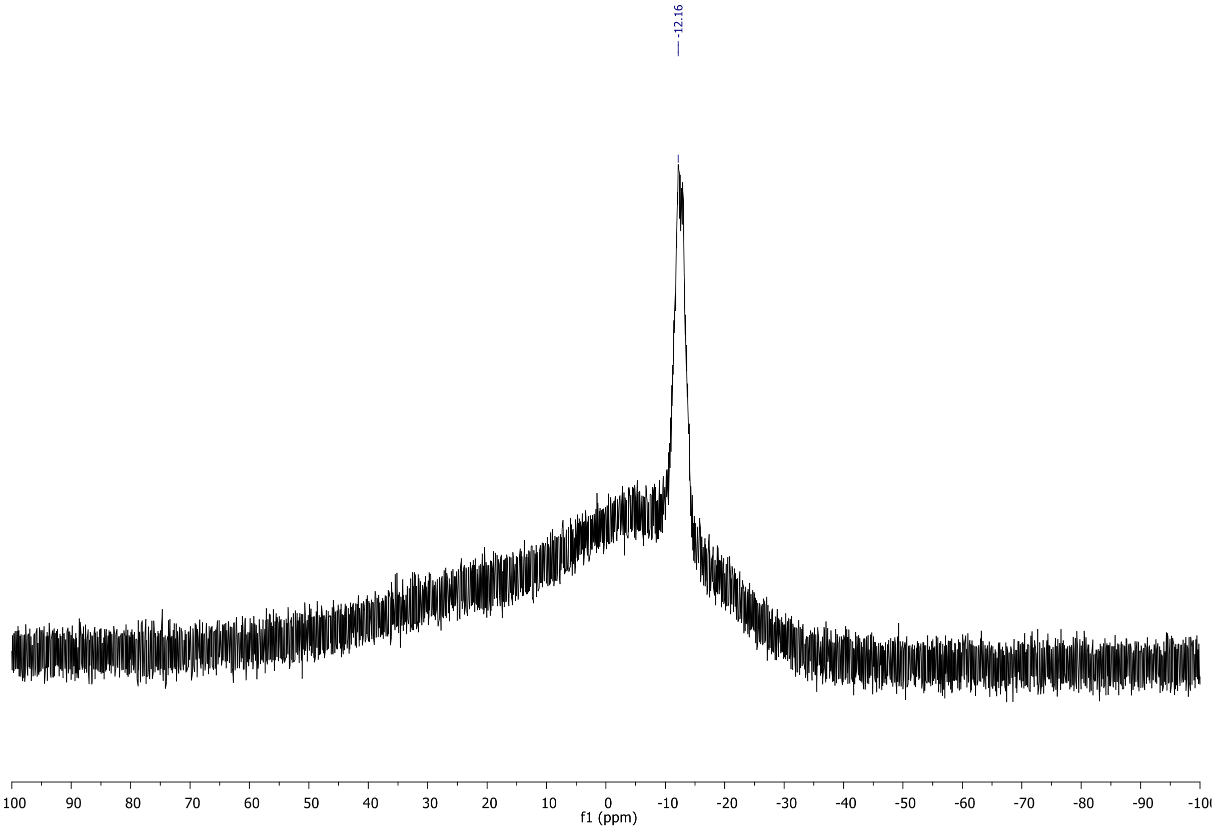

Synthesis of mB3

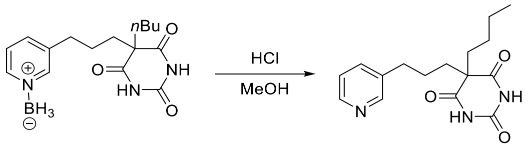

To a mixture of **mB3-BH$_3$** (274 mg, 0.86 mmol) and methanol (15 mL) [note: *not dissolved even at* RT] at 0 °C, HCl (*conc*., 100 µL, 1.2 mmol) was added. After 15 minutes, the cooling bath was removed and the mixture was stirred at RT until full conversion was reached (2 hours), which was checked by LCMS. A saturated solution of $NaHCO_3$ (4 mL) was added (pH > 8) and then a saturated solution of NH_4Cl (30 mL) was added (pH < 7). The mixture was extracted with dichloromethane (4 × 50 mL), dried over $MgSO_4$, the solvents were removed under reducer pressure. A combiflash of the residue on silica (Celite loading, PE/EtOAc, EtOAc: 0%→100%) provided after drying in vacuum oven overnight the title compound (170 mg, 65% yield) as a white crystalline solid.

^{1}H NMR (CD$_3$OD, 400 MHz, 298 K): δ 8.37 – 8.35 (m, 2H), 7.67 – 7.64 (m, 1H), 7.35 (ddd, *J* = 8.0, 5.0, 1.0 Hz, 1H), 2.64 (t, *J* = 7.5 Hz, 2H), 2.00 – 1.85 (m, 4H), 1.58 – 1.50 (m, 2H), 1.33 – 1.22 (m, 2H), 1.21 – 1.10 (m, 2H), 0.86 (t, *J* = 7.0 Hz, 3H).
^{13}C NMR (CD$_3$OD, 101 MHz, 298 K): δ 175.0, 151.4, 150.0, 147.8, 139.0, 138.2, 125.2, 56.9, 40.0, 39.0, 33.4, 28.1, 27.4, 23.7, 14.0.

HR-MS (ESI): Calculated for $C_{16}H_{22}N_3O_3$ $[M+H]^+$ 304.1661, found: 304.1668 (Δ = 2.3 ppm)

FT-IR (thin film): 3222, 3103, 2959, 2930, 2859, 1698, 1596, 1580, 1413, 1385, 1357, 1325 cm^{-1}.

MP: 184 – 186 °C

EA: Required for $C_{16}H_{21}N_3O_3$: C 63.35, H 6.98, N 13.85; found: C 62.92, H 7.09, N 13.66.

LCMS Method: Col3-MeCN-FAST

^{1}H NMR

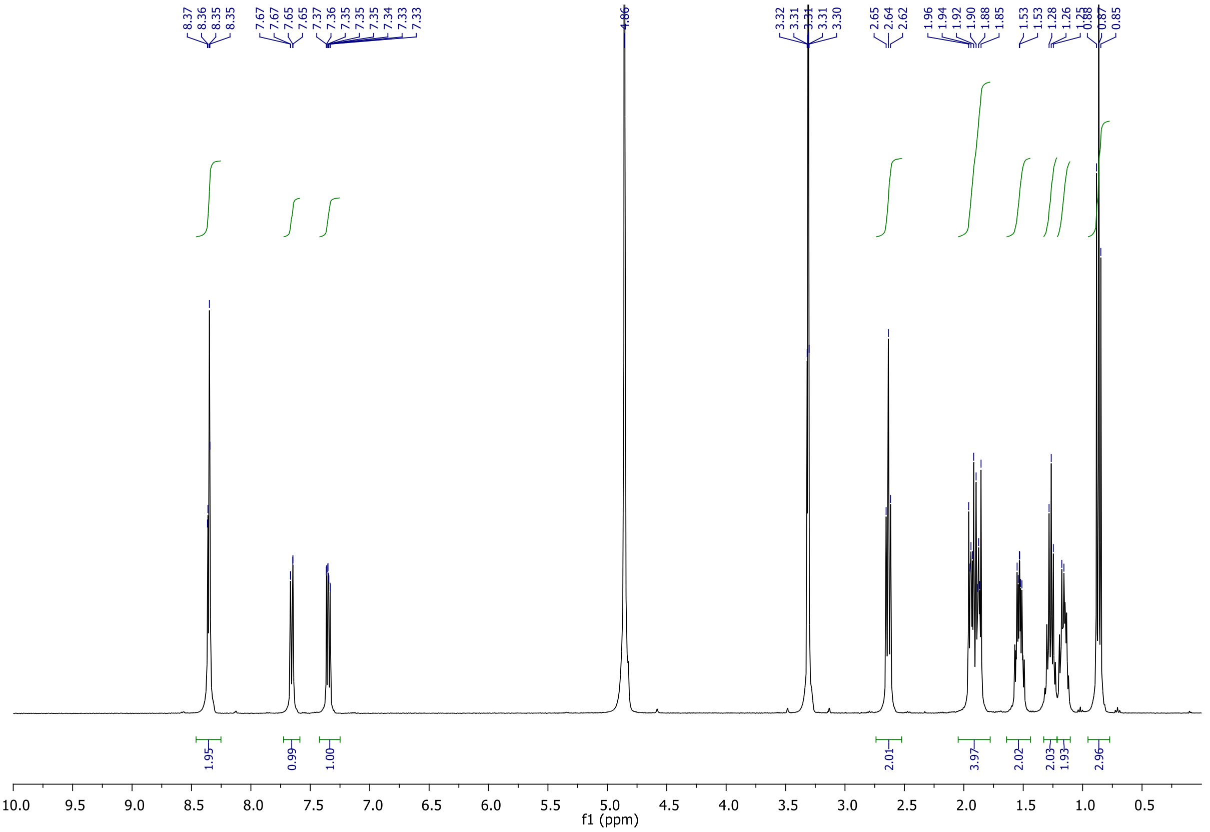

^{13}C NMR

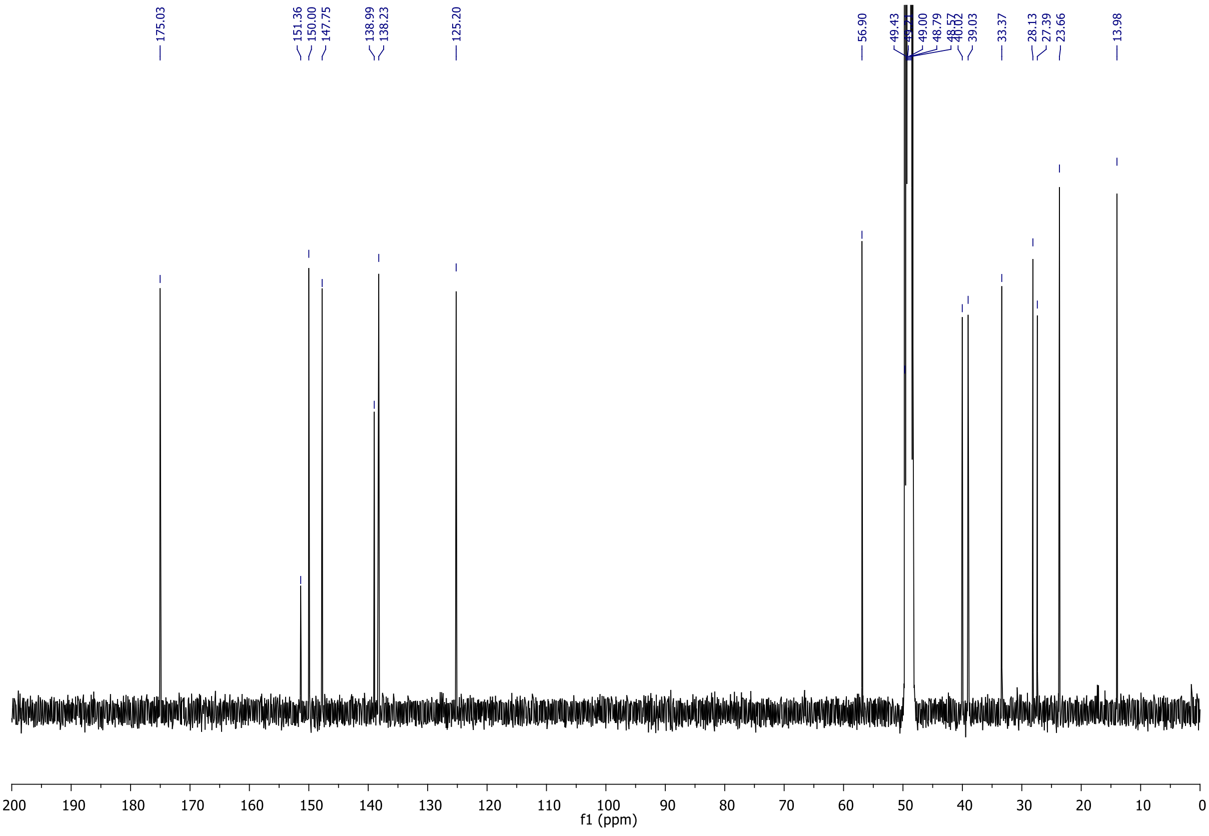

Synthesis of 7

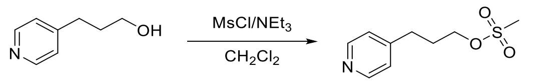

To a solution of 3-(pyridin-4-yl)propan-1-ol (1.29 g, 9.4 mmol) and NEt_3 (4 mL) in CH_2Cl_2 (50 mL) was added methanesulfonyl chloride (1.2 mL, 15.5 mmol, 1.6 eq) at 0 °C in small parts over the period of 90 minutes. The still-cool solution was washed with H_2O (50 mL) and brine (25 mL), dried with $MgSO_4$ and solvents were removed under reducer pressure (at no more than 30 °C). A combiflash of the residue on silica gel (PE/EtOAc, EtOAc: 0%→100%) provided the title compound as transparent oil (1.53 g, 77% yield).

^{1}H NMR ($CDCl_3$, 400 MHz, 298 K): δ 8.50 (dd, *J* = 4.5, 1.5 Hz, 2H), 7.11 (d, *J* = 6.0 Hz, 2H), 4.22 (t, *J* = 6.0 Hz, 2H), 2.99 (s, 3H), 2.76 – 2.72 (m, 2H), 2.11 – 2.04 (m, 2H).
^{13}C NMR ($CDCl_3$, 101 MHz, 298 K): δ 149.9, 149.2, 123.7, 68.5, 37.4, 30.9, 29.6.

¹H NMR

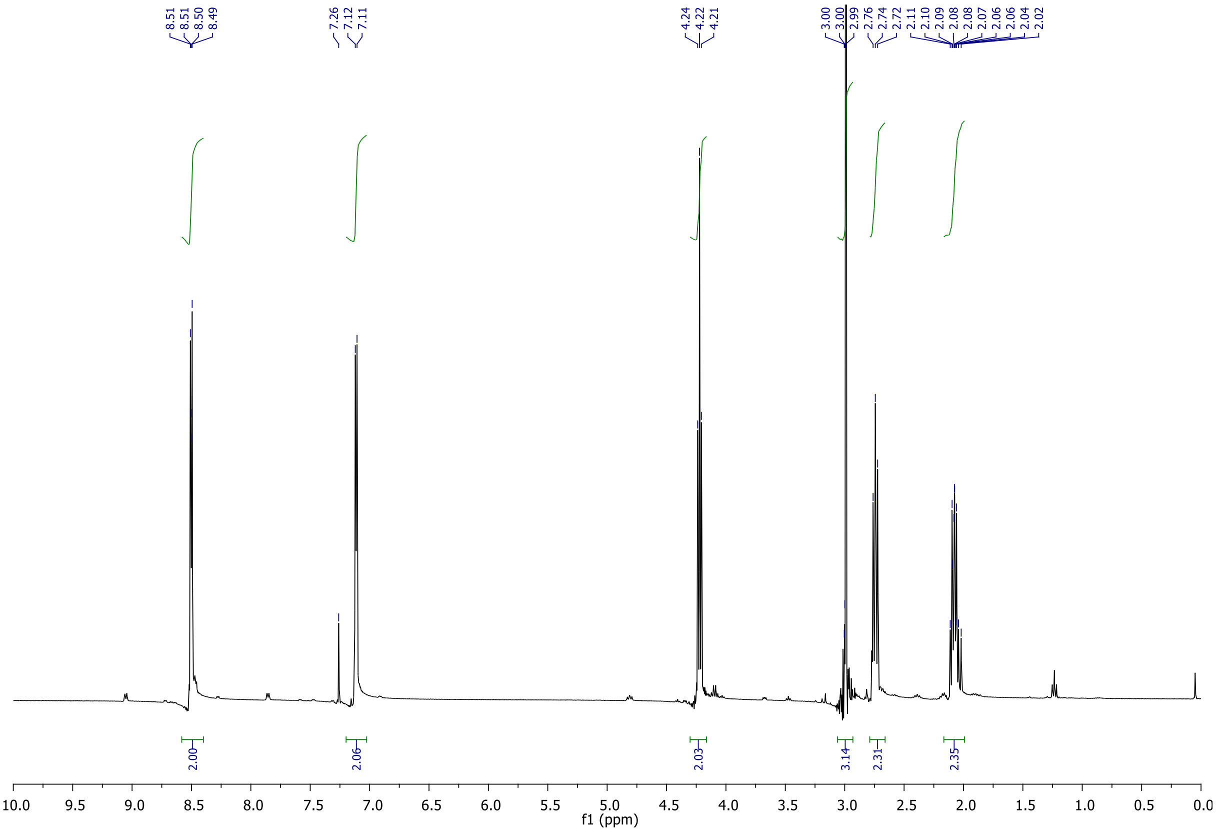

¹³C NMR

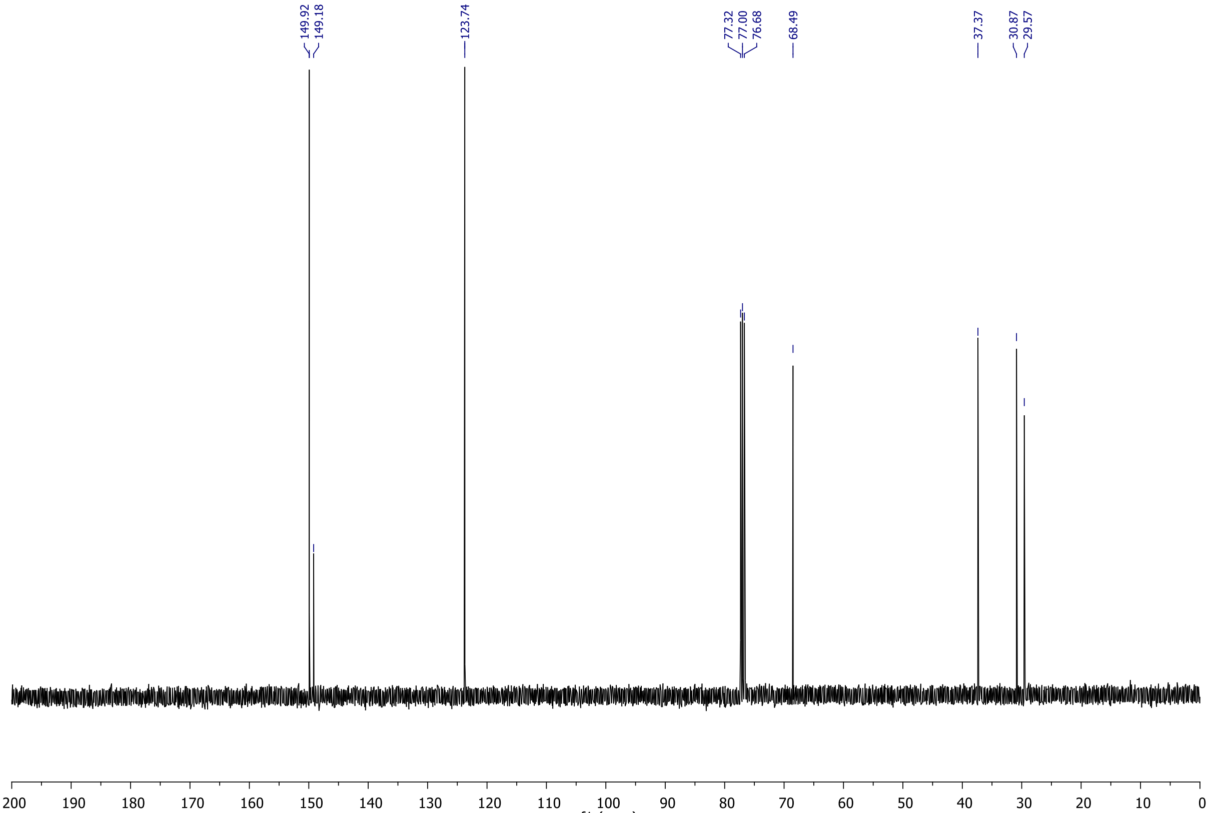

Synthesis of 7-BH_3

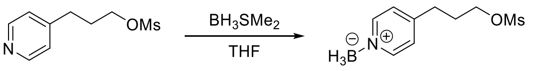

7 (1.53 g, 7.11 mmol) was flushed with nitrogen. Then THF (10 mL) was added and the reaction mixture was cooled to –78 °C. To this solution, BH_3•SMe_2 (4.0 mL, 2.0M solution in THF, 8 mmol) was added dropwise at –78 °C. After 30 minutes of stirring, the cooling bath was removed. After stirring at RT for 2.5 hours, the mixture was cooled again to –78 °C and methanol (3 mL) was added dropwise. After 10 minutes, the cooling bath was removed and the mixture was allowed to heat to room temperature and then solvents were removed under reduced pressure. A combiflash of the residue on silica (Celite loading, PE/EtOAc; EtOAc: 0%→100%) provided the title compound (1.44 g, 88% yield) as transparent oil, which crystalise upon standing in the freezer over a couple of days/weeks to form a white solid.

^{1}H NMR ($CDCl_3$, 400 MHz, 298 K): δ 8.47 (d, J = 6.5 Hz, 2H), 7.34 (d, J = 6.5 Hz, 2H), 4.25 (t, J = 6.0 Hz, 2H), 3.02 (s, 3H), 2.90 – 2.86 (m, 2H), 2.63 (br, 3H), 2.14 – 2.07 (m, 2H).
^{1}H NMR ($CDCl_3$, 400 MHz, 298 K, ^{11}B decoupled): δ 8.47 (d, J = 6.0 Hz, 2H), 7.34 (d, J = 6.0 Hz, 2H), 4.25 (t, J = 6.0 Hz, 2H), 3.03 (s, 3H), 2.90 – 2.86 (m, 2H), 2.54 (s, 3H), 2.14 – 2.07 (m, 2H).
^{13}C NMR ($CDCl_3$, 101 MHz, 298 K): δ 153.7, 147.3, 125.3, 68.0, 37.5, 31.0, 29.2.
^{11}B NMR ($CDCl_3$, 128 MHz, 298 K, normal glass): δ –12.7 (d, J = 86 Hz).

HR-MS (ESI): Calculated for $C_9H_{15}BNO_3S$ $[M–H]^+$ 228.0866, found: 228.0855 (Δ = 4.7 ppm)

FT-IR (thin film): 3021, 2938, 2361, 2312, 2282, 1631, 1349, 1170 cm^{-1}.

MP: 58 °C (melts and decomposes)

LCMS Method: Col3_ MeCN_FAST_5%-35%

^{1}H NMR

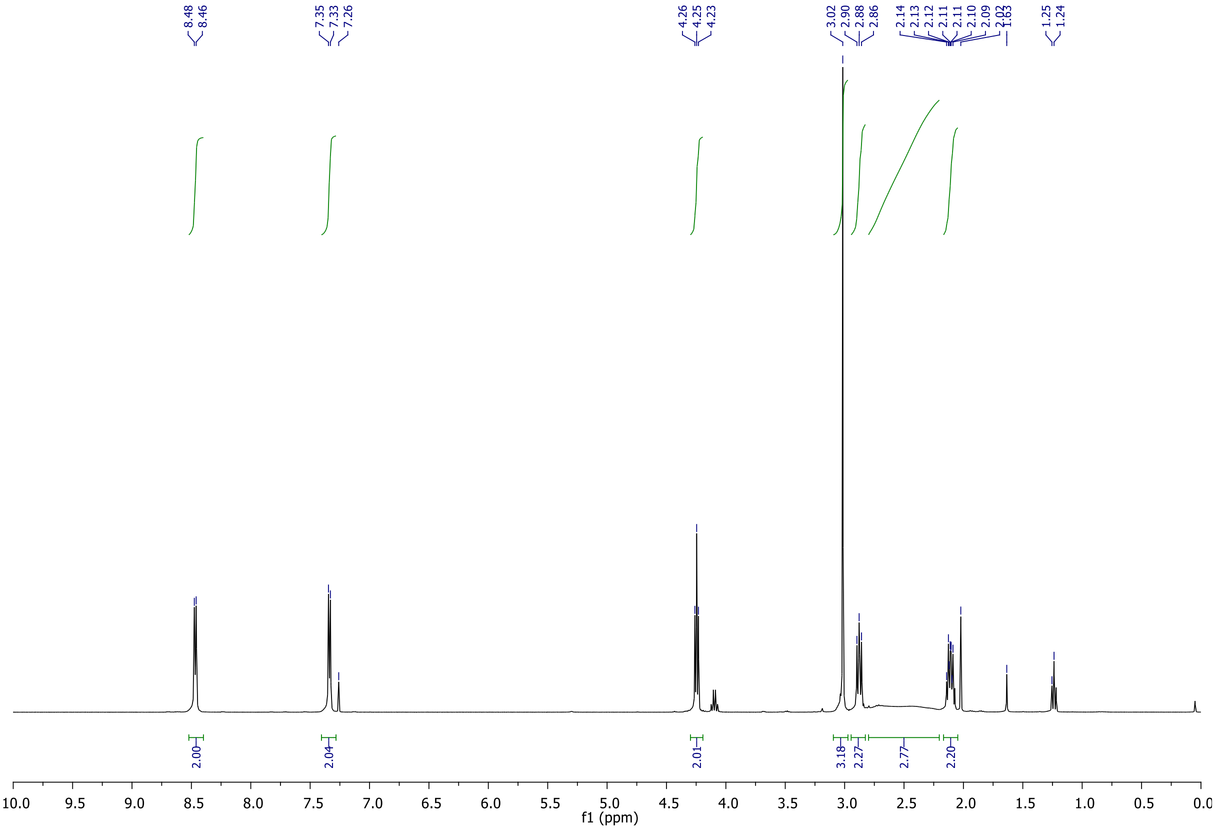

^{1}H NMR (^{11}B decoupled)

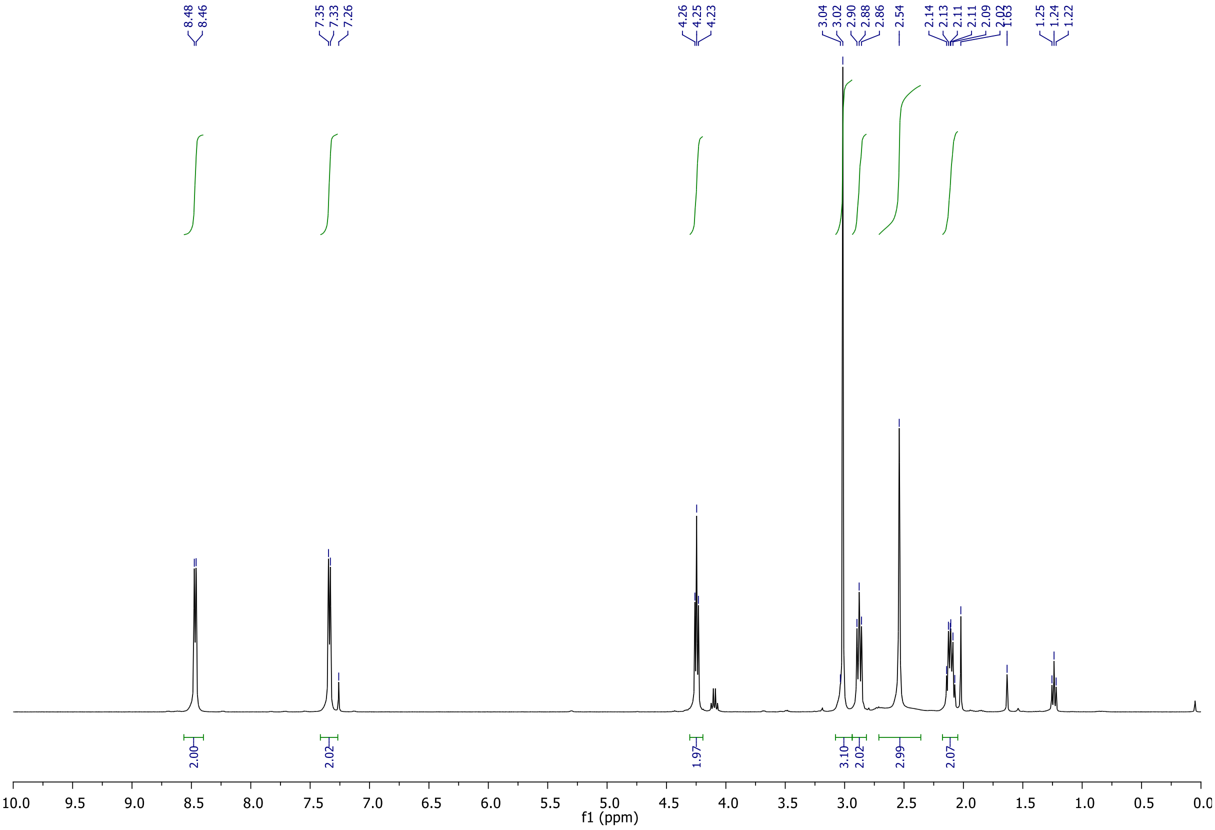

^{13}C NMR

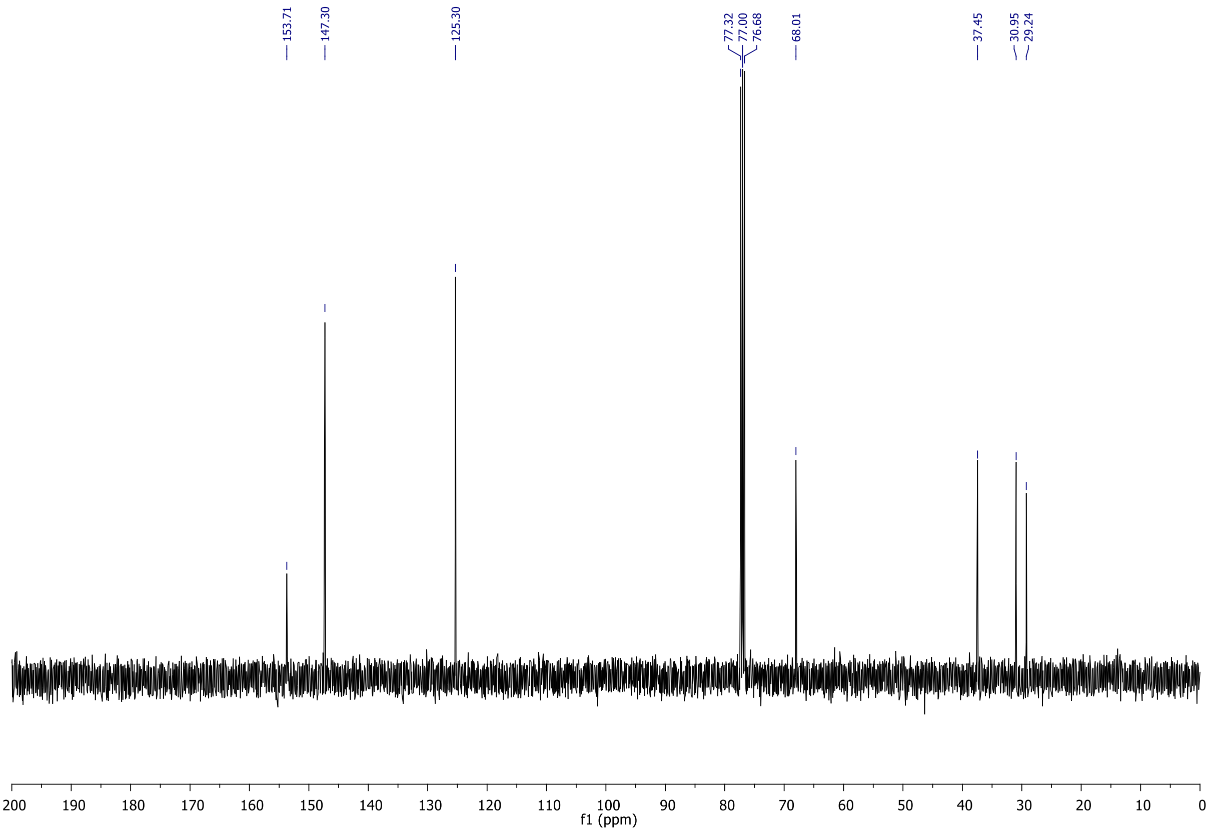

^{11}B NMR (normal glass)

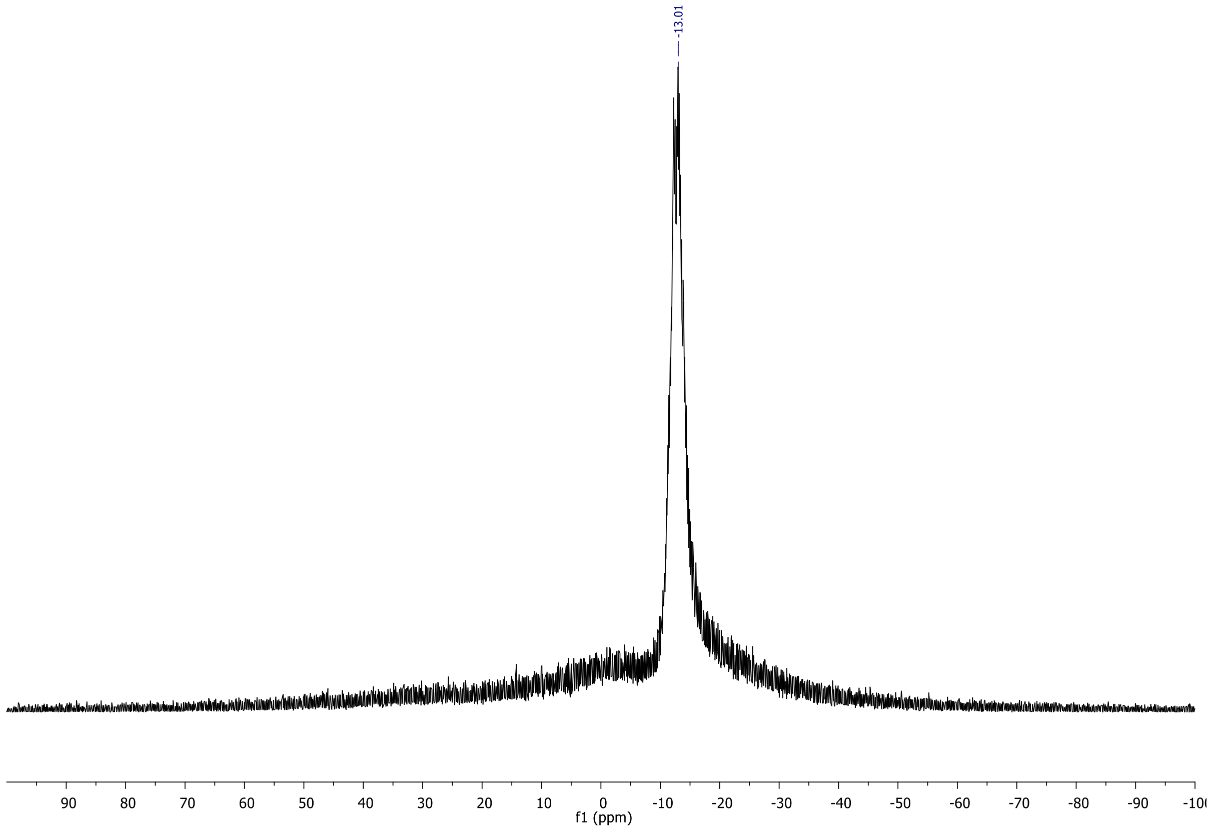

Synthesis of 8-BH_3 and 8

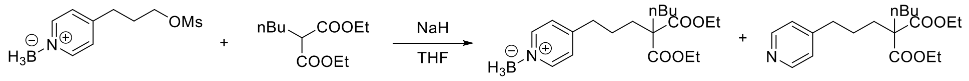

NaH (60% in mineral oil, 334 mg, 8.35 mmol) was flushed with nitrogen. THF (10 mL) was added and the mixture was cooled to 0 °C. Then diethyl-2-butylmalonate (1.9 mL, 8.54 mmol) was added dropwise. After 30 minutes of stirring **7-BH_3** (631 mg, 2.76 mmol) was added as a solid. The mixture was heated at 60 °C for 5 hours and then after cooling to RT, water (20 mL) was added and the mixture was extracted with dichloromethane (3 × 50 mL). The organic phase was dried over $MgSO_4$ and the solvents were removed under reduced pressure. A combiflash of the residue on silica (Celite loading, PE/EtOAc, EtOAc: 0%→50%) provided after drying in vacuum oven overnight products **8-BH_3** (472 mg, 49% crude yield) as transparent oil and **8** (110 mg, 23% crude yield) as yellowish oil. (*Both products contained an additional impurity and were used in the next reactions without further purification.*)

LCMS Method: Col1-MeCN-SLOW

Compound **8-BH_3**:
^{1}H NMR ($CDCl_3$, 400 MHz, 298 K): δ 8.46 (d, J = 6.0 Hz, 2H), 7.29 (d, J = 6.5 Hz, 2H), 4.22 – 4.10 (m, 4H), 2.73 (t, J = 7.5 Hz, 2H), 1.90 – 1.83 (m, 4H), 1.62 – 1.54 (m, 2H), 1.33 – 1.25 (m, 2H), 1.22 (t, J = 7.0 Hz, 6H), 1.13 – 1.05 (m, 2H), 0.88 (t, J = 7.5 Hz, 3H).
^{1}H NMR ($CDCl_3$, 400 MHz, 298 K, ^{11}B decoupled): δ 8.46 (d, J = 6.0 Hz, 2H), 7.29 (d, J = 6.0 Hz, 2H), 4.21 – 4.10 (m, 4H), 2.73 (t, J = 7.5 Hz, 2H), 2.56 (s, 3H), 1.90 – 1.83 (m, 4H), 1.62 – 1.53 (m, 2H), 1.33 – 1.27 (m, 2H), 1.22 (t, J = 7.0 Hz, 6H), 1.12 – 1.05 (m, 2H), 0.88 (t, J = 7.3 Hz, 3H).
^{13}C NMR ($CDCl_3$, 101 MHz, 298 K): δ 171.5, 155.0, 147.2, 146.8, 125.2, 121.7, 61.2, 57.2, 35.2, 32.3, 31.8, 26.1, 24.3, 22.8, 14.1, 13.8.
^{11}B NMR ($CDCl_3$, 128 MHz, 298 K, normal glass): δ –13.0 (d, J = 93 Hz).

MS: 389.26 [M+CHC_3CN–H]$^+$

FT-IR (thin film): 2959, 2935, 2872, 2362, 2308, 2280, 1725, 1630, 1562, 1504, 1463, 1439, 1367, 1258, 1200, 1169, 1086, 1024 cm^{-1}.

Compound **8**:
^{1}H NMR ($CDCl_3$, 400 MHz, 298 K): δ 8.48 (d, J = 5.5 Hz, 2H), 7.09 (d, J = 6.0 Hz, 2H), 4.19 – 4.12 (m, 4H), 2.61 (t, J = 7.5 Hz, 2H), 1.91 – 1.83 (m, 4H), 1.57 – 1.49 (m, 2H), 1.34 – 1.24 (m, 2H), 1.21 (t, J = 7.0 Hz, 6H), 1.11 – 1.03 (m, 2H), 0.88 (t, J = 7.5 Hz, 3H).
^{13}C NMR ($CDCl_3$, 101 MHz, 298 K): δ 171.7, 150.7, 149.7, 123.8, 61.1, 57.3, 35.2, 32.0, 31.6, 26.1, 24.6, 22.9, 14.1, 13.9.

MS: 336.22 [M+H]$^+$

FT-IR (thin film): 2958, 2936, 2872, 1728, 1601, 1464, 1415, 1367, 1257, 1200, 1160 cm^{-1}.

8-BH$_3$: ^{1}H NMR

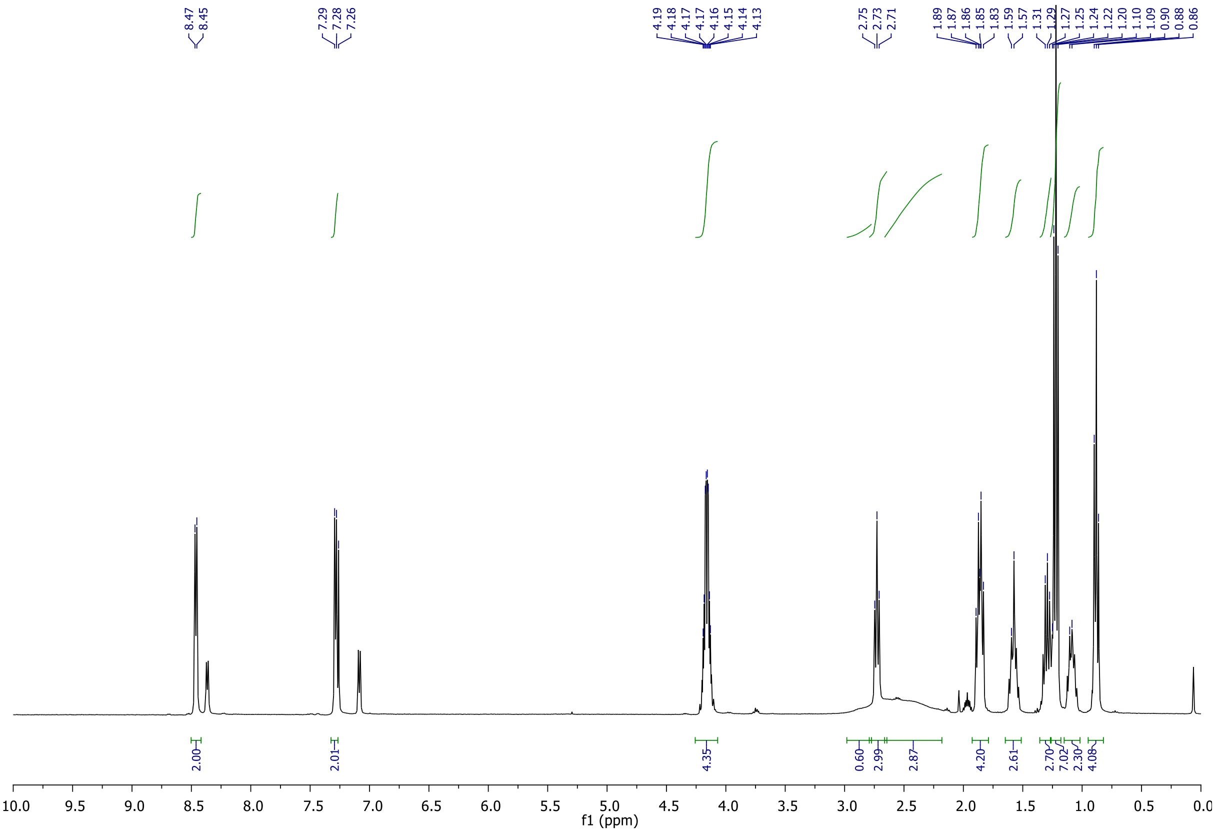

8-BH$_3$: ^{1}H NMR (^{11}B decoupled)

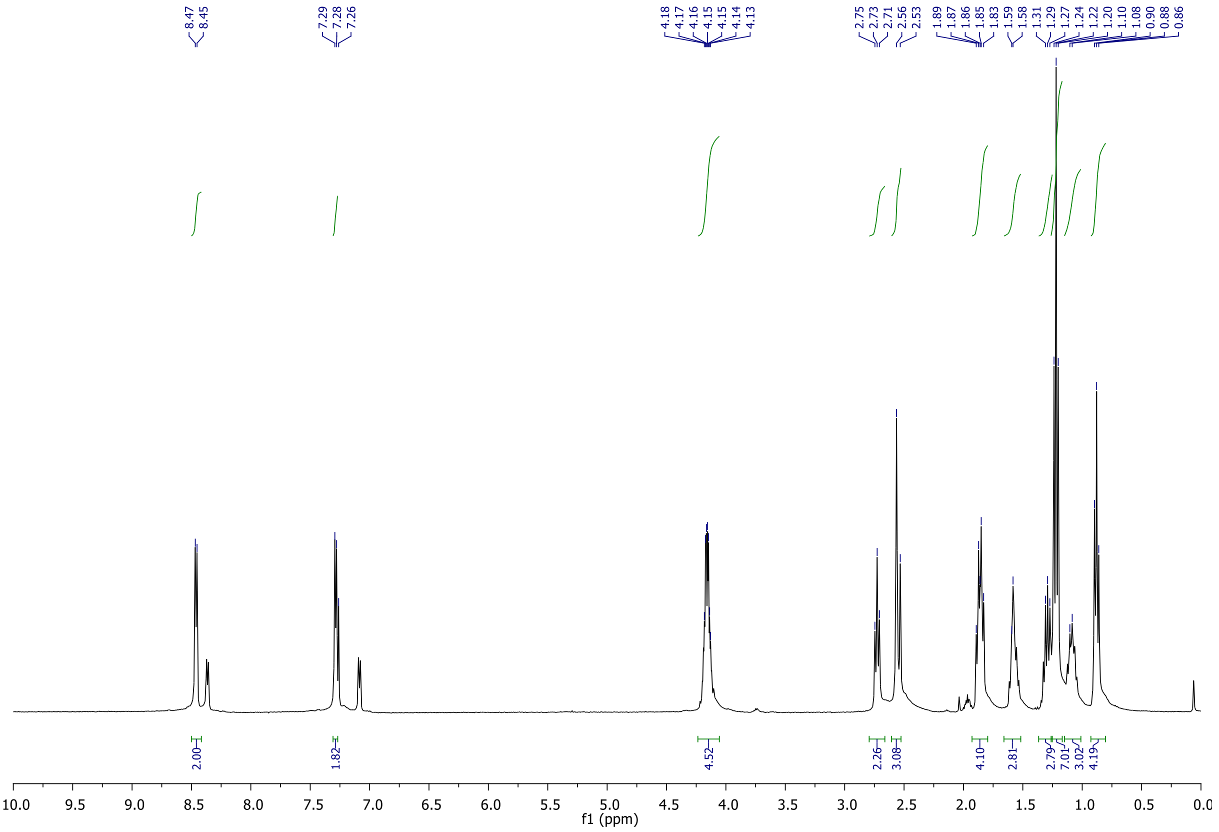

8-BH$_3$: ^{13}C NMR

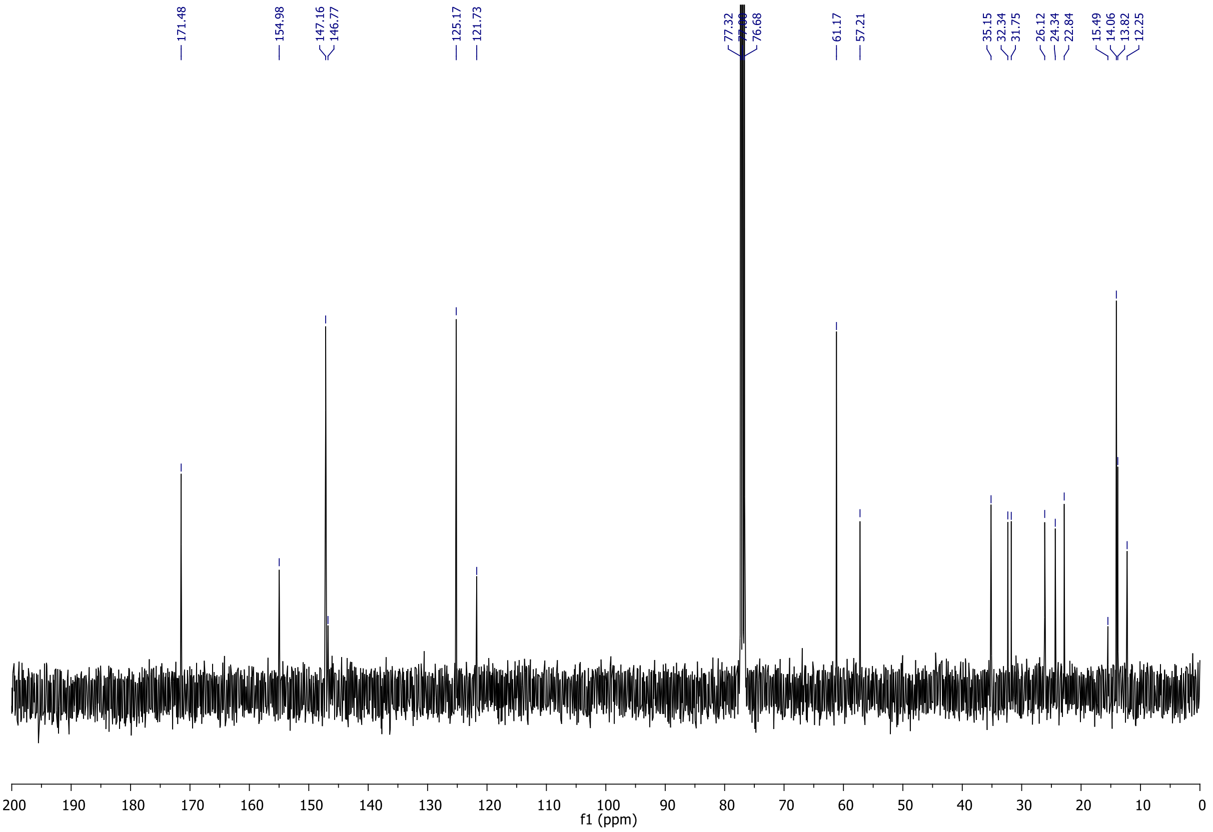

8-BH$_3$: ^{11}B (normal glass)

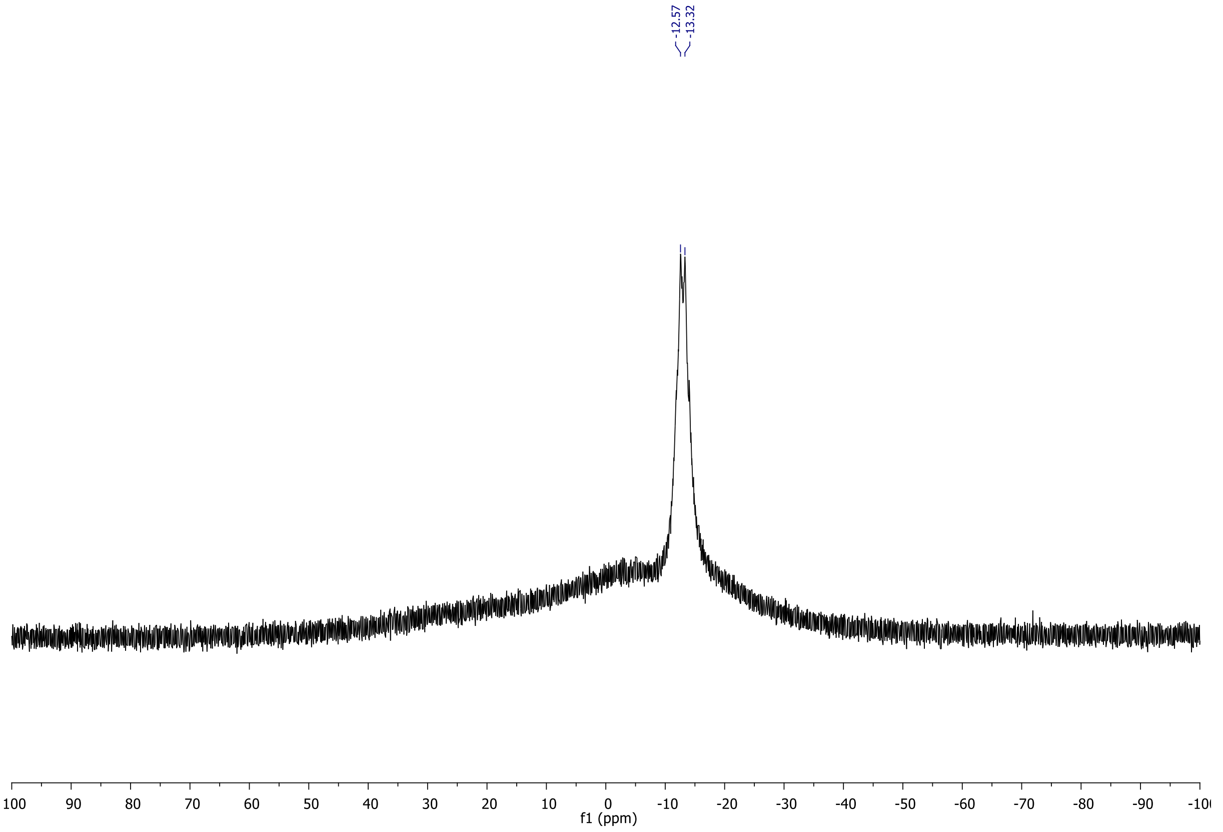

8: ^{1}H NMR

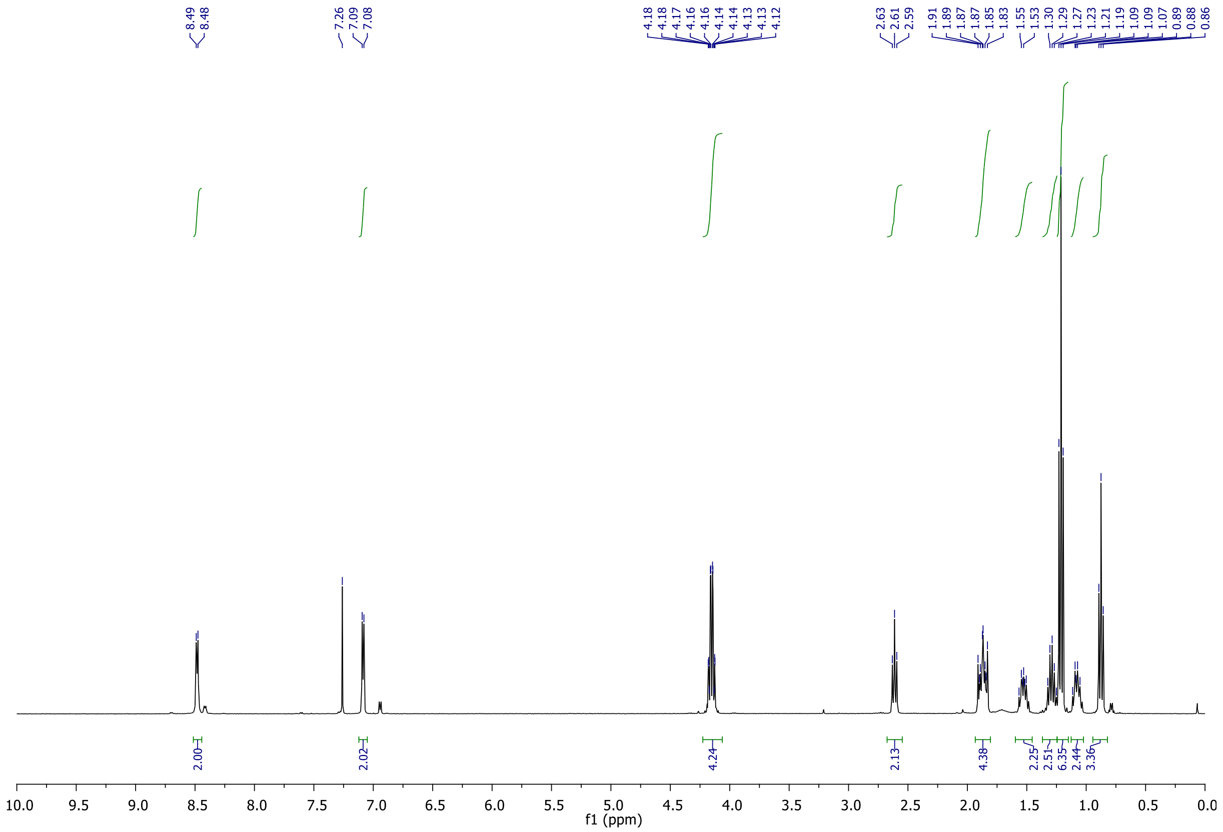

8: ^{13}C NMR

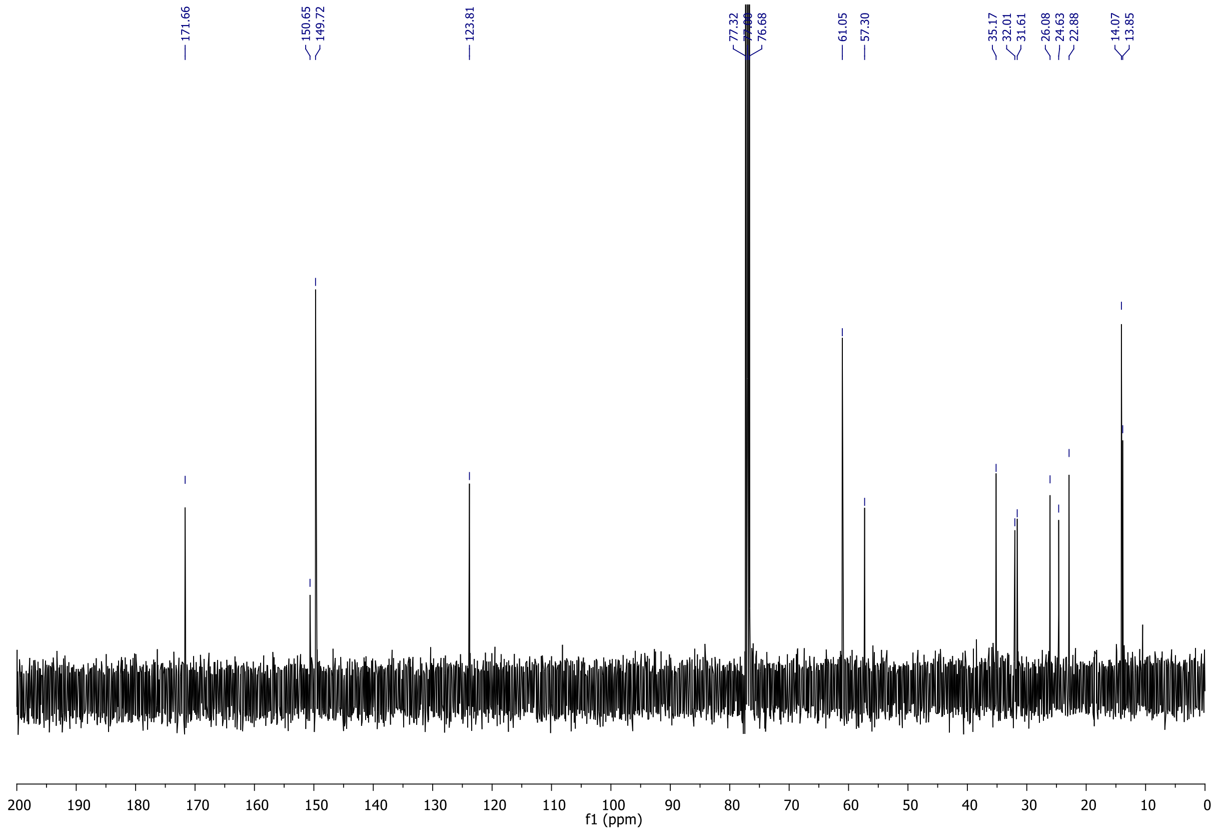

Synthesis of pB3

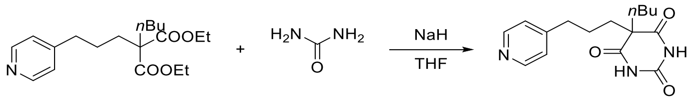

NaH (60% in mineral oil, 57 mg, 1.4 mmol) was flushed with nitrogen and DMF (2 mL) was added. The mixture was cooled to 0 °C and urea (197 mg, 3.28 mmol) was added. After 90 minutes of stirring, **8** (102 mg, 0.30 mmol) in DMF (4 mL) was added at 0 °C. The mixture was stirred for 5 days at RT and a saturated solution of NH_4Cl (5 mL) was added with cooling to 0 °C. Water (10 mL) was added and the mixture was extracted with dichloromethane (3 × 50 mL). The collected organic phase was dried over $MgSO_4$ and the solvents were removed under reduced pressure (60 °C, <14 mBar). A combliflash of residue on silica (Celite loading, PE/EtOAc, EtOAc: 0%→100%) provided after drying in vacuum oven at 40 °C overnight the title product (350 mg, 50% yield) as a white crystalline solid.

^{1}H NMR (CD_3OD, 400 MHz, 298 K): δ 8.40 (dd, J = 4.5, 1.5 Hz, 2H), 7.25 (dd, J = 4.5, 1.5 Hz, 2H), 2.65 (t, J = 7.5 Hz, 2H), 1.95 – 1.92 (m, 2H), 1.90 – 1.85 (m, 2H), 1.60 – 1.51 (m, 2H), 1.33 – 1.23 (m, 2H), 1.20 – 1.12 (m, 2H), 0.87 (t, J = 7.0 Hz, 3H).
^{13}C NMR (CD_3OD, 101 MHz, 298 K): δ 175.0, 153.3, 149.9, 125.6, 56.9, 40.1, 39.0, 35.6, 28.1, 26.5, 23.7, 14.0.

HR-MS (ESI): Required for $C_{16}H_{22}N_3O_3$ $[M+H]^+$ 304.1656, found: 304.1644 (Δ = 3.91 ppm).

FT-IR (thin film): 3226, 3100, 2959, 2930, 2858, 1723, 1701, 1607, 1417, 1384, 1357, 1260, 1217 cm^{-1}.

MP: 174 – 176 °C

EA: Required for $C_{16}H_{21}N_3O_3$: C 63.35, H 6.98, N 13.85; found: C 62.67, H 7.01, N 13.44.

LCMS Method: Col3_ MeCN_FAST_5%-35%

^{1}H NMR

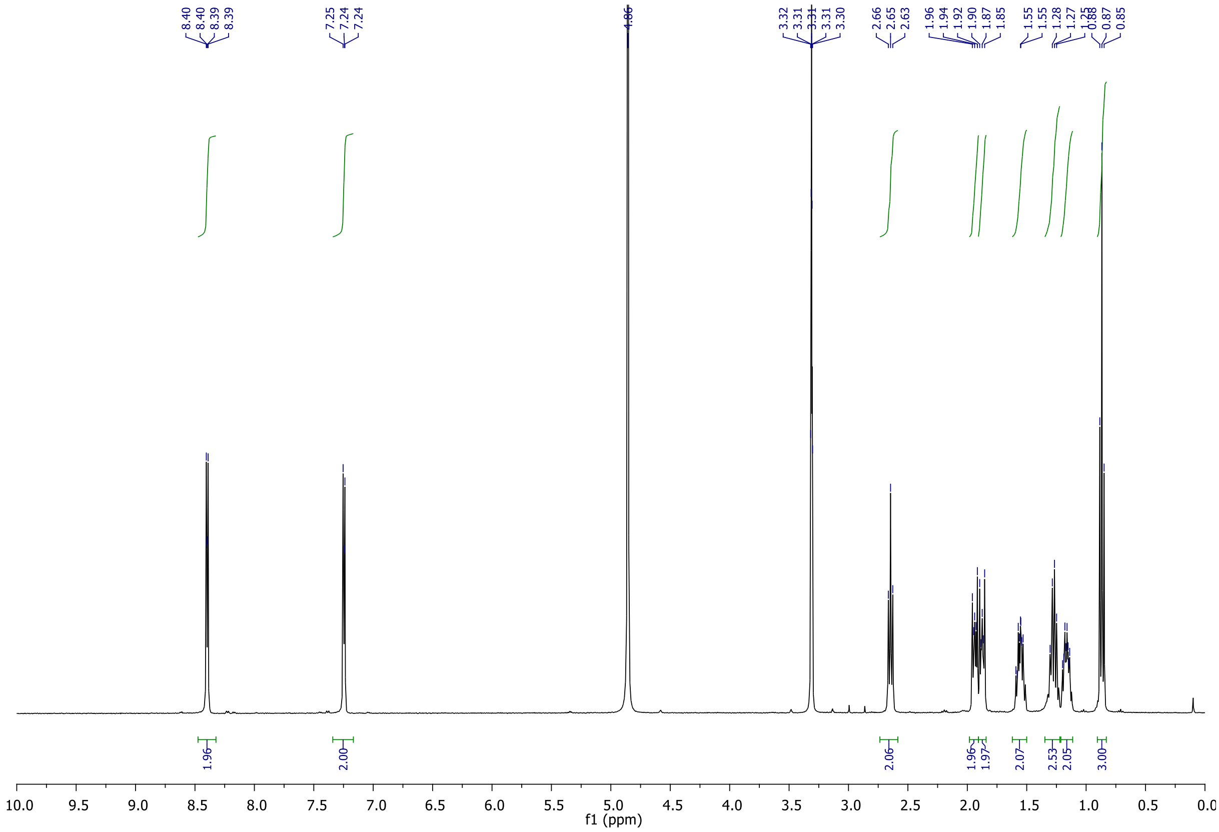

^{13}C NMR

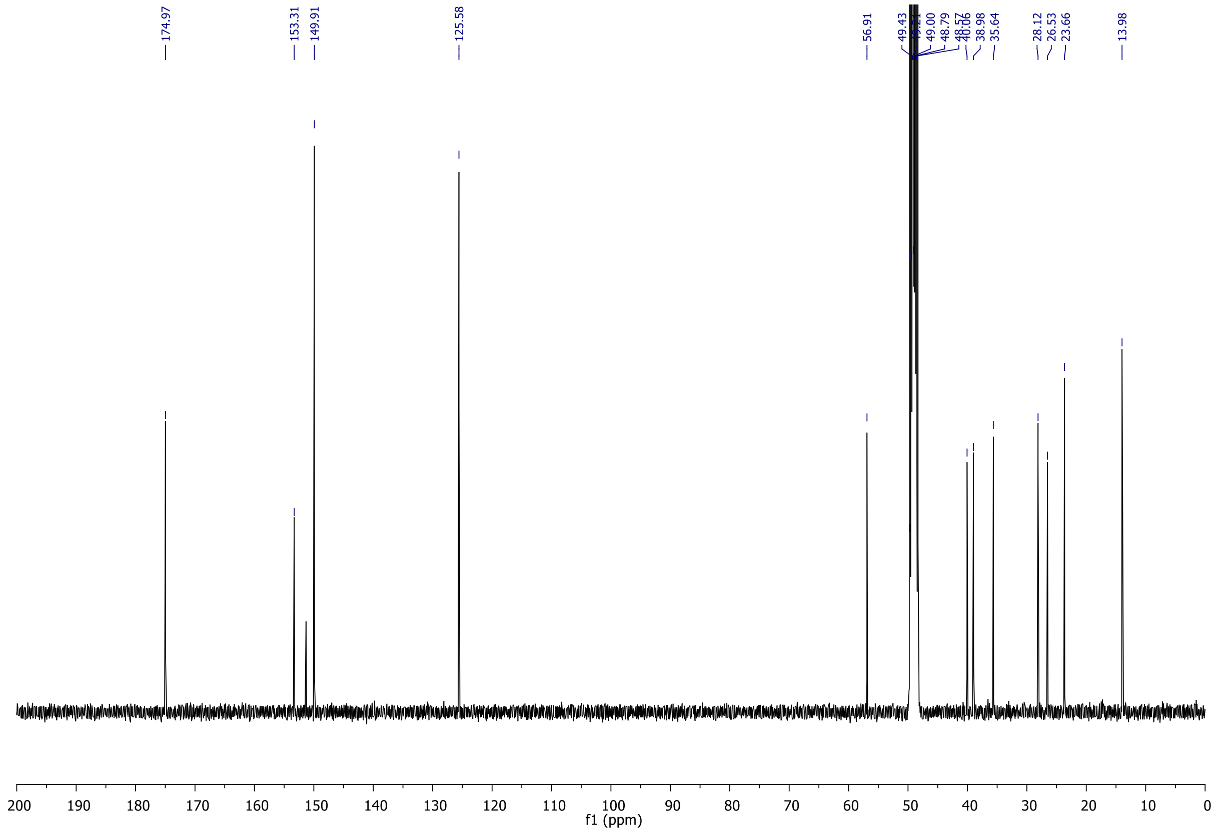

S3: UV-vis-NIR titrations

S3.1 General

The UV-vis-NIR titrations were recorded on a Cary 60 UV-Vis machine (Agilent Technologies) at 298 K. Freshly opened $CHCl_3$ (HPLC quality, stabilised with amylene) was used as a solvent and was filtered over basic alumina plug before use. A host solution with known concentration was prepared and fraction of this solution was transferred into a glass cuvette. Then, a guest solution with known concentration was prepared. A change of UV-vis-NIR absorption upon addition of aliquots of guest solution was followed. The observed changes of UV-vis-NIR absorbance were analysed using a purpose-written fitting macro in Microsoft Excel. A 1:1 binding isotherm was used to fit the experimental data assuming that all six porphyrin units of **c-P6** coordinate a pyridine ligand and act identically and independently. The experiments were measured at least two times on at least two different days with freshly prepared solutions in order to eliminate possible systematic errors. The results are stated as average values and errors are quoted as two times the standard deviation.

S3.2 Two component titrations

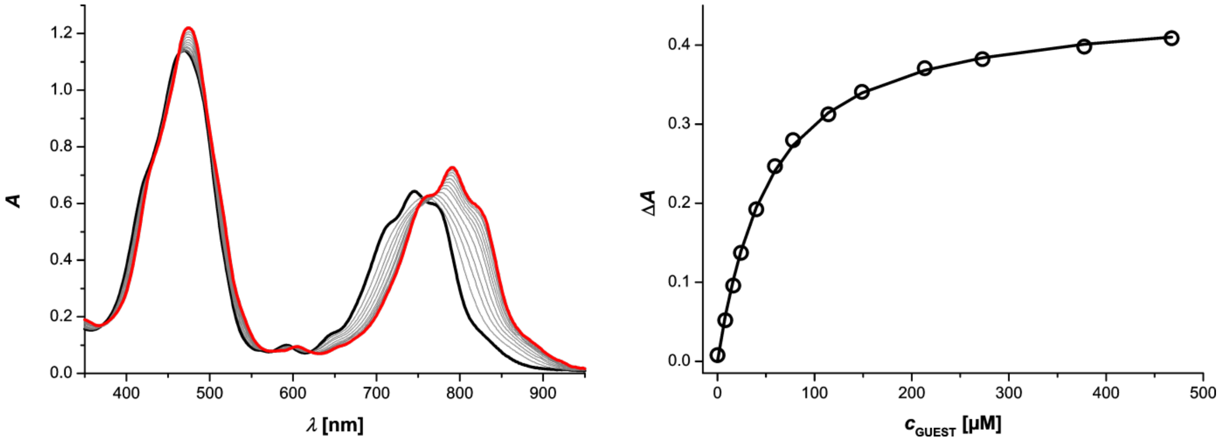

Figure S1. UV-vis-NIR titration ($CHCl_3$, 298 K) of **pPy** to **c-P6**. UV-vis-NIR spectra are shown on the left with the spectrum of unbound **c-P6** in thick black line and the end spectrum in red thick line. On the right are shown the experimental data at 833 nm (circles) and calculated values (line) based on a 1:1 binding isotherm assuming that the six porphyrin units of **c-P6** act identically and independently.

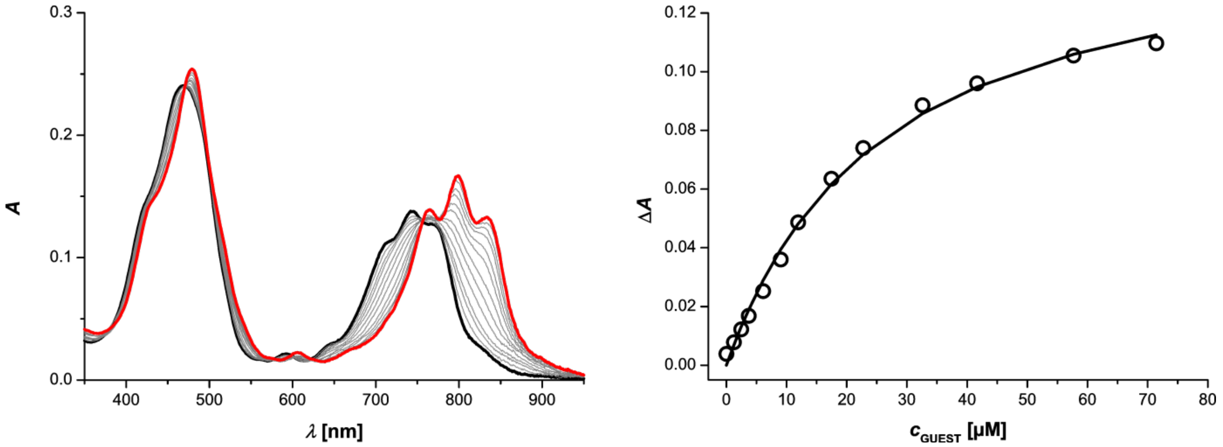

Figure S2. UV-vis-NIR titration ($CHCl_3$, 298 K) of **mB3** to **c-P6**. UV-vis-NIR spectra are shown on the left with the spectrum of unbound **c-P6** in thick black line and the end spectrum in red thick line. On the right are shown the experimental data at 842 nm (circles) and calculated values (line) based on a 1:1 binding isotherm assuming that the six porphyrin units of **c-P6** act identically and independently.

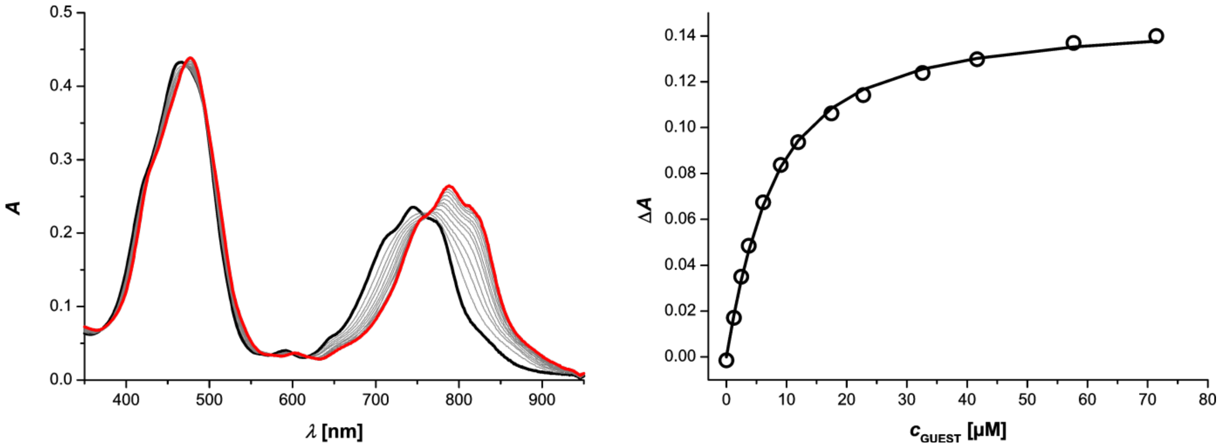

Figure S3. UV-vis-NIR titration ($CHCl_3$, 298 K) of **pB3** to **c-P6**. UV-vis-NIR spectra are shown on the left with the spectrum of unbound **c-P6** in thick black line and the end spectrum in red thick line. On the right are shown the experimental data at 831 nm (circles) and calculated values (line) based on a 1:1 binding isotherm assuming that the six porphyrin units of **c-P6** act identically and independently.

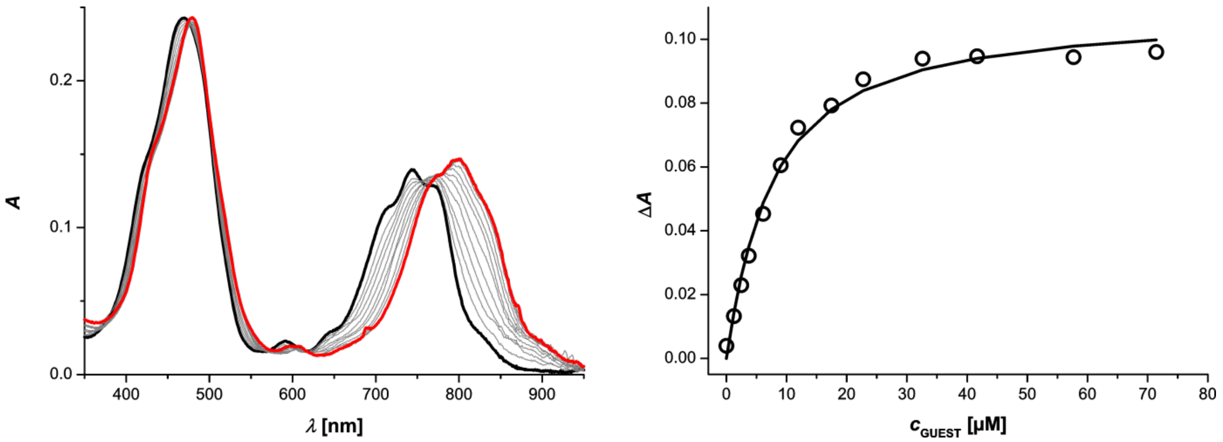

Figure S4. UV-vis-NIR titration ($CHCl_3$, 298 K) of **mA1** to **c-P6**. UV-vis-NIR spectra are shown on the left with the spectrum of unbound **c-P6** in thick black line and the end spectrum in red thick line. On the right are shown the experimental data at 831 nm (circles) and calculated values (line) based on a 1:1 binding isotherm assuming that the six porphyrin units of **c-P6** act identically and independently.

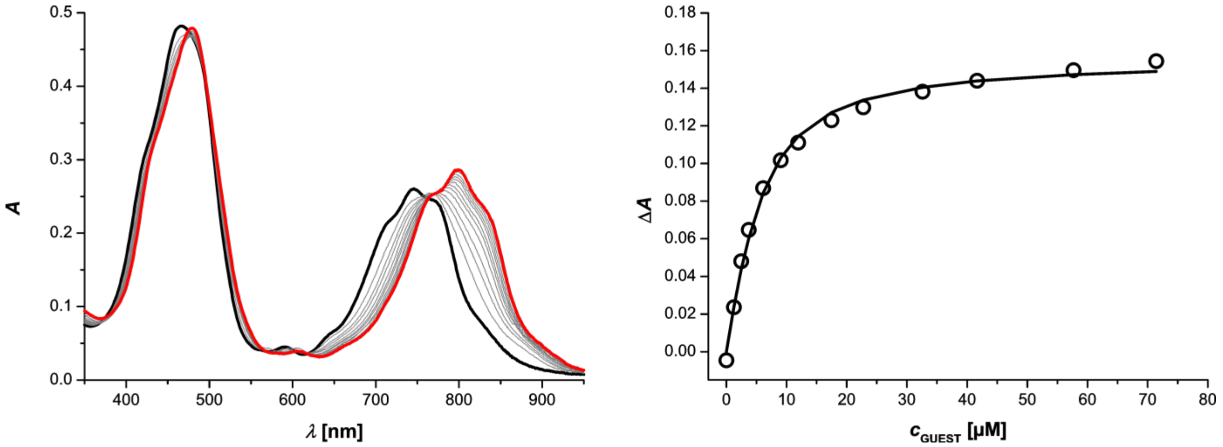

Figure S5. UV-vis-NIR titration ($CHCl_3$, 298 K) of **mA2** to **c-P6**. UV-vis-NIR spectra are shown on the left with the spectrum of unbound **c-P6** in thick black line and the end spectrum in red thick line. On the right are shown the experimental data at 836 nm (circles) and calculated values (line) based on a 1:1 binding isotherm assuming that the six porphyrin units of **c-P6** act identically and independently.

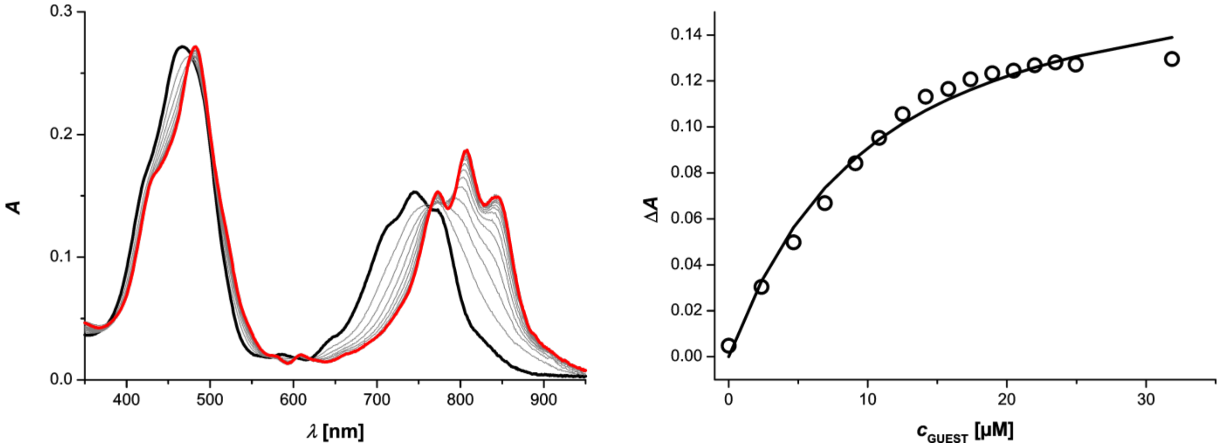

Figure S6. UV-vis-NIR titration ($CHCl_3$, 298 K) of **pA1** to **c-P6**. UV-vis-NIR spectra are shown on the left with the spectrum of unbound **c-P6** in thick black line and the end spectrum in red thick line. On the right are shown the experimental data at 852 nm (circles) and calculated values (line) based on a 1:1 binding isotherm assuming that the six porphyrin units of **c-P6** act identically and independently. A 1:1 binding isotherm struggles to describe the experimental data correctly, but the concentration of ligand for half bound is 7 μM and the best fit for a 1:1 binding isotherm was used to compare this ligand with other systems.

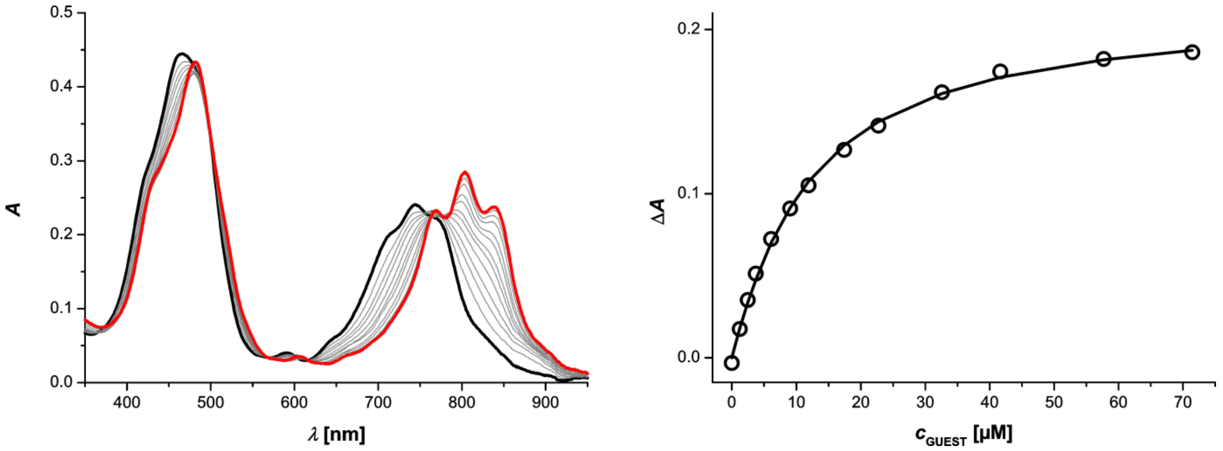

Figure S7. UV-vis-NIR titration ($CHCl_3$, 298 K) of **pA2** to **c-P6**. UV-vis-NIR spectra are shown on the left with the spectrum of unbound **c-P6** in thick black line and the end spectrum in red thick line. On the right are shown the experimental data at 843 nm (circles) and calculated values (line) based on a 1:1 binding isotherm assuming that the six porphyrin units of **c-P6** act identically and independently.

S3.3 Three component titrations fitted with a 1:1 isotherm

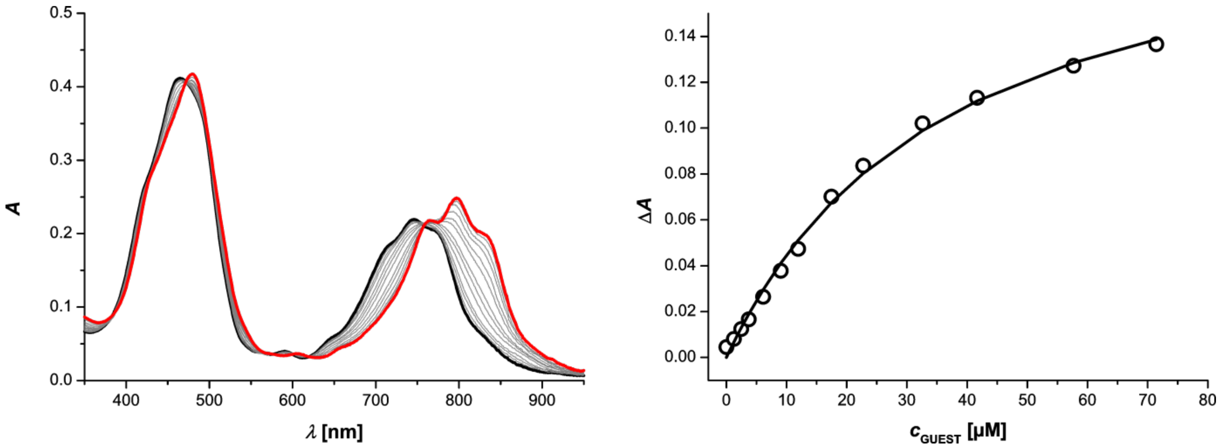

Figure S8. UV-vis-NIR titration ($CHCl_3$, 298 K) of a 1:1 mixture of **A** and **mB3** to **c-P6**. UV-vis-NIR spectra are shown on the left with the spectrum of unbound **c-P6** in thick black line and the end spectrum in red thick line. On the right are shown the experimental data at 831 nm (circles) and calculated values (line) based on a 1:1 binding isotherm assuming that the six porphyrin units of **c-P6** act identically and independently.

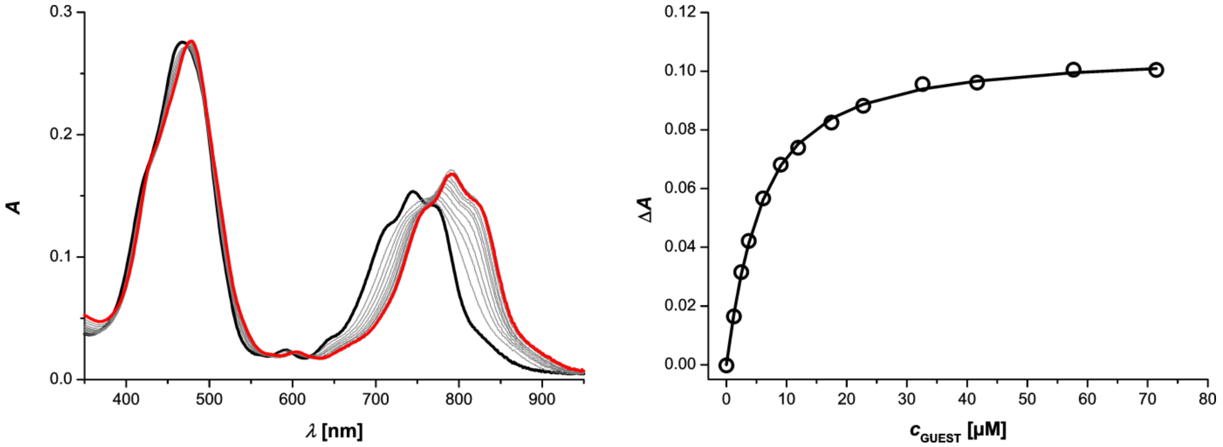

Figure S9. UV-vis-NIR titration ($CHCl_3$, 298 K) of a 1:1 mixture of **A** and **pB3** to **c-P6**. UV-vis-NIR spectra are shown on the left with the spectrum of unbound **c-P6** in thick black line and the end spectrum in red thick line. On the right are shown the experimental data at 831 nm (circles) and calculated values (line) based on a 1:1 binding isotherm assuming that the six porphyrin units of **c-P6** act identically and independently.

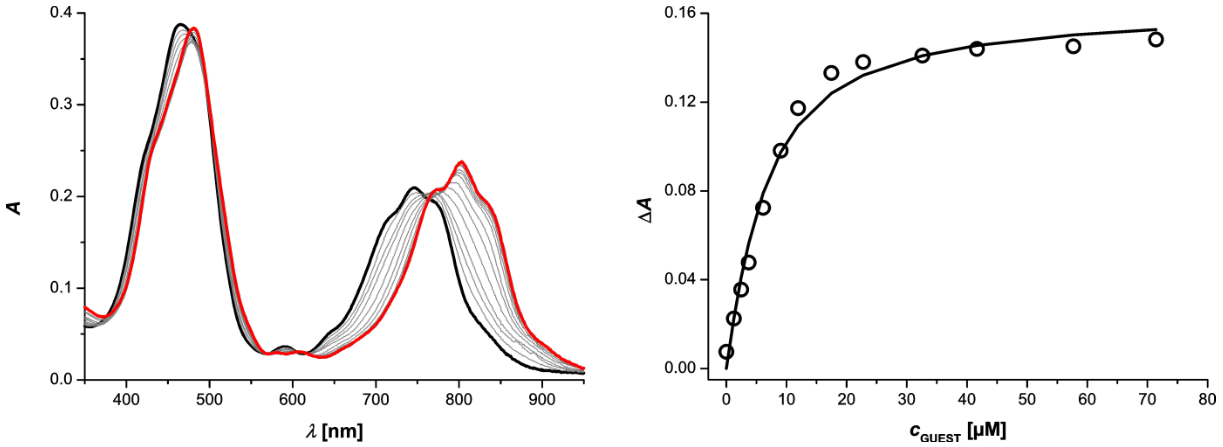

Figure S10. UV-vis-NIR titration ($CHCl_3$, 298 K) of a 1:1 mixture of **B** and **mA1** to **c-P6**. UV-vis-NIR spectra are shown on the left with the spectrum of unbound **c-P6** in thick black line and the end spectrum in red thick line. On the right are shown the experimental data at 831 nm (circles) and calculated values (line) based on a 1:1 binding isotherm assuming that the six porphyrin units of **c-P6** act identically and independently.

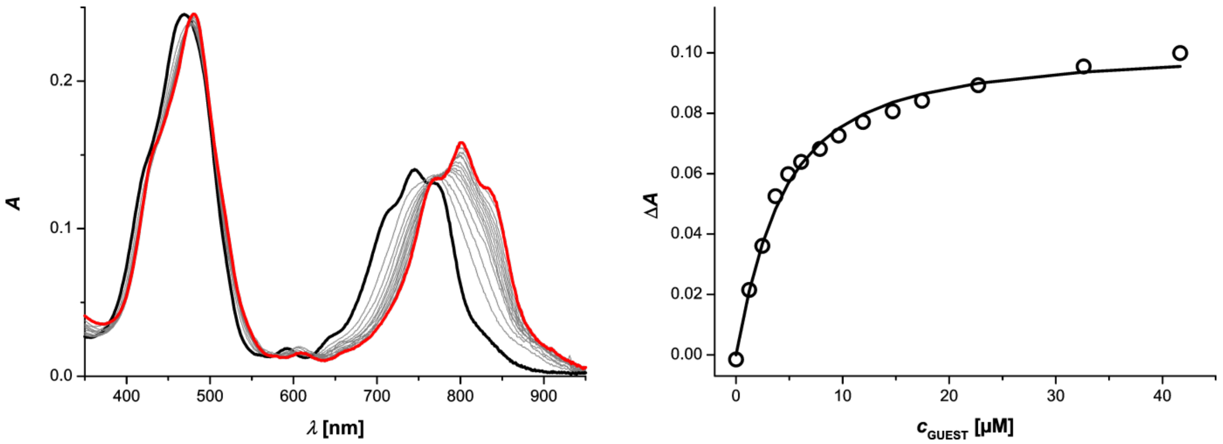

Figure S11. UV-vis-NIR titration ($CHCl_3$, 298 K) of a 1:1 mixture of **B** and **mA2** to **c-P6**. UV-vis-NIR spectra are shown on the left with the spectrum of unbound **c-P6** in thick black line and the end spectrum in red thick line. On the right are shown the experimental data at 836 nm (circles) and calculated values (line) based on a 1:1 binding isotherm assuming that the six porphyrin units of **c-P6** act identically and independently.

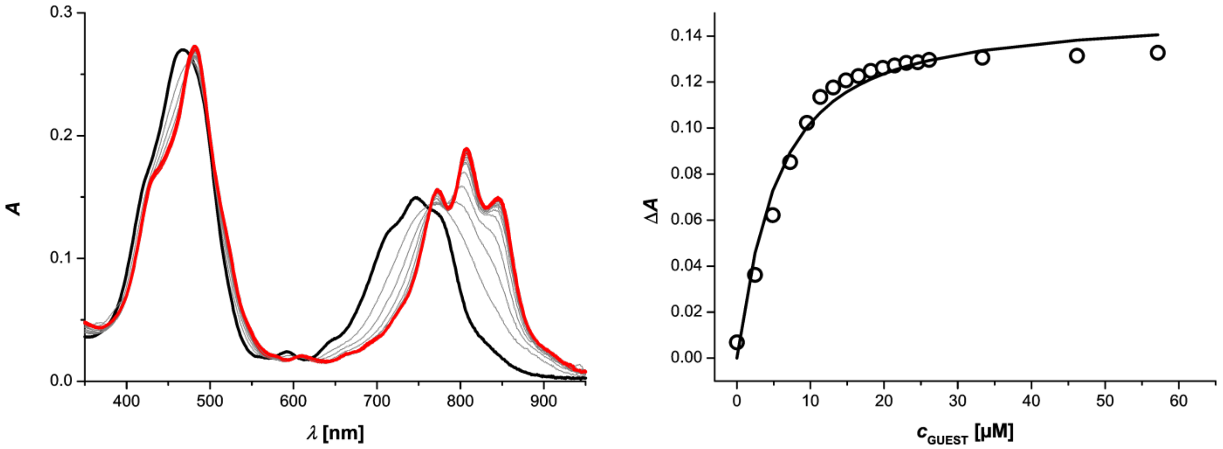

Figure S12. UV-vis-NIR titration ($CHCl_3$, 298 K) of a 1:1 mixture of **B** and **pA1** to **c-P6**. UV-vis-NIR spectra are shown on the left with the spectrum of unbound **c-P6** in thick black line and the end spectrum in red thick line. On the right are shown the experimental data at 852 nm (circles) and calculated values (line) based on a 1:1 binding isotherm assuming that the six porphyrin units of **c-P6** act identically and independently.

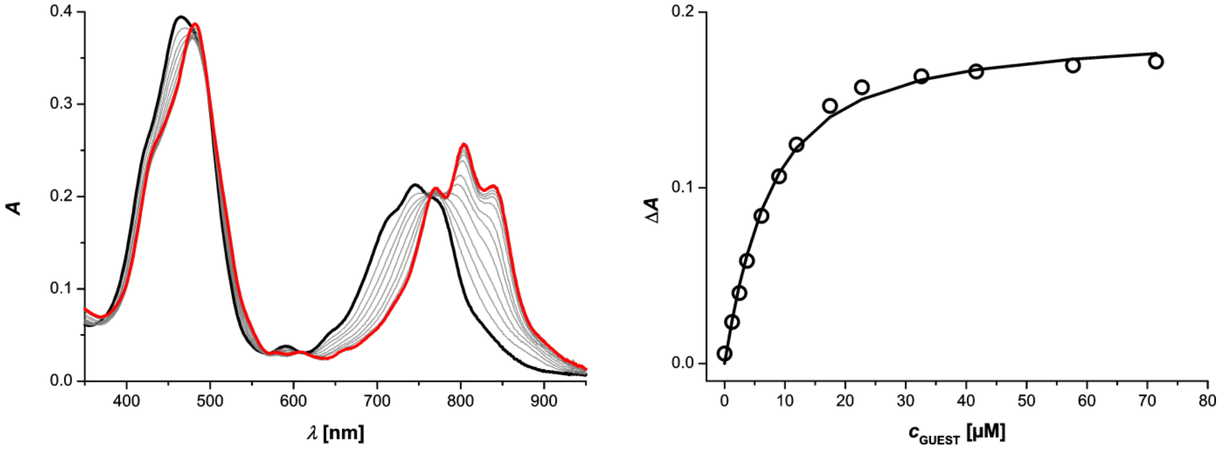

Figure S13. UV-vis-NIR titration ($CHCl_3$, 298 K) of a 1:1 mixture of **B** and **pA2** to **c-P6**. UV-vis-NIR spectra are shown on the left with the spectrum of unbound **c-P6** in thick black line and the end spectrum in red thick line. On the right are shown the experimental data at 843 nm (circles) and calculated values (line) based on a 1:1 binding isotherm assuming that the six porphyrin units of **c-P6** act identically and independently.

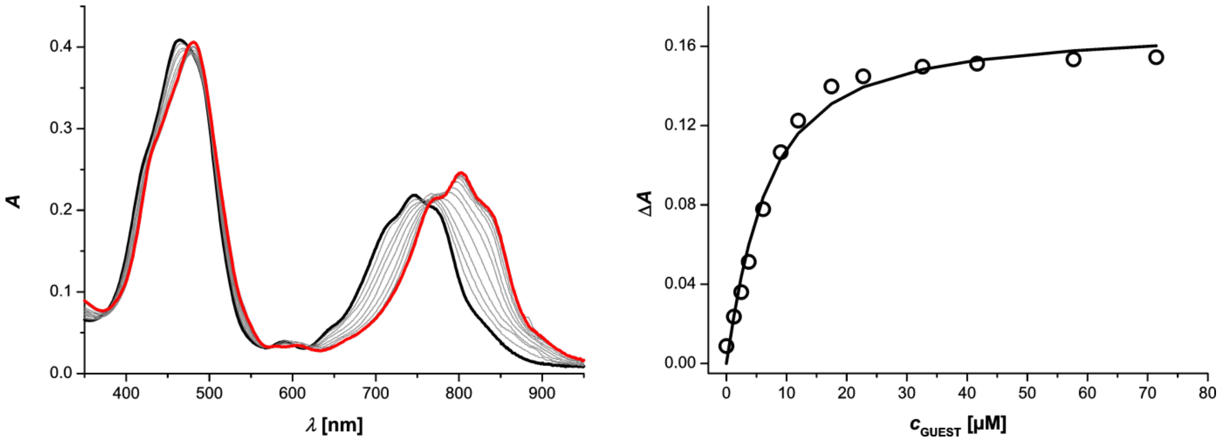

Figure S14. UV-vis-NIR titration ($CHCl_3$, 298 K) of a 1:1 mixture of **C** and **mA1** to **c-P6**. UV-vis-NIR spectra are shown on the left with the spectrum of unbound **c-P6** in thick black line and the end spectrum in red thick line. On the right are shown the experimental data at 831 nm (circles) and calculated values (line) based on a 1:1 binding isotherm assuming that the six porphyrin units of **c-P6** act identically and independently.

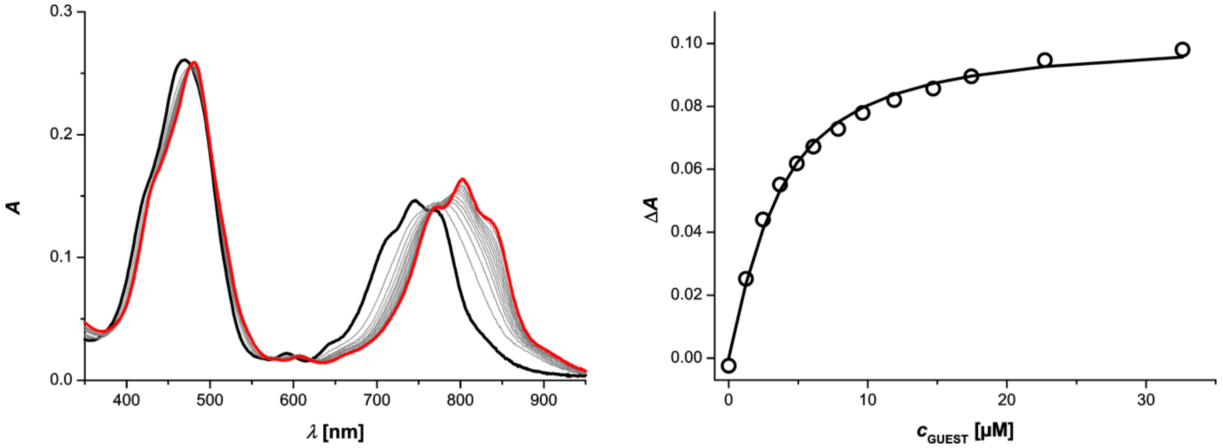

Figure S15. UV-vis-NIR titration ($CHCl_3$, 298 K) of a 1:1 mixture of **C** and **mA2** to **c-P6**. UV-vis-NIR spectra are shown on the left with the spectrum of unbound **c-P6** in thick black line and the end spectrum in red thick line. On the right are shown the experimental data at 836 nm (circles) and calculated values (line) based on a 1:1 binding isotherm assuming that the six porphyrin units of **c-P6** act identically and independently.

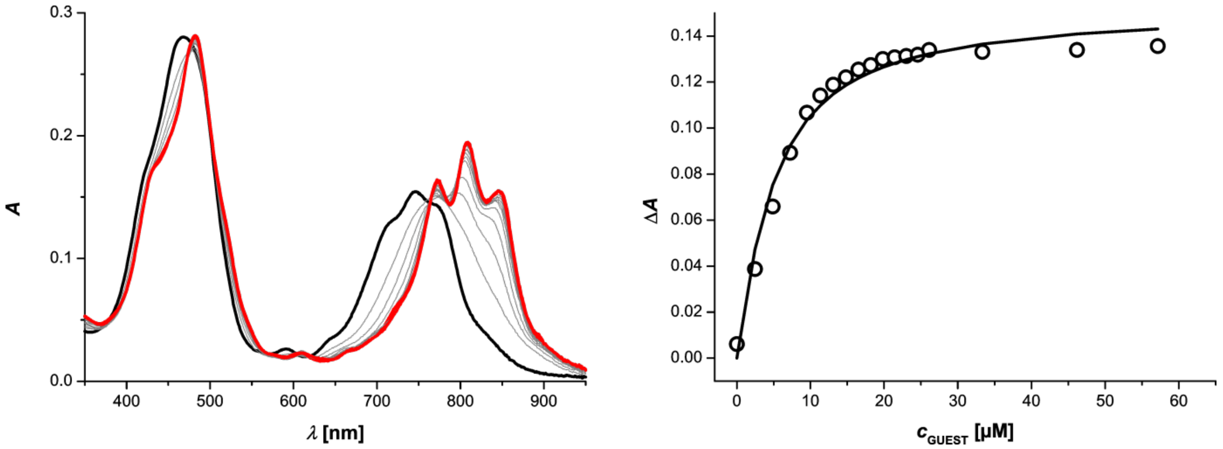

Figure S16. UV-vis-NIR titration ($CHCl_3$, 298 K) of a 1:1 mixture of **C** and **pA1** to **c-P6**. UV-vis-NIR spectra are shown on the left with the spectrum of unbound **c-P6** in thick black line and the end spectrum in red thick line. On the right are shown the experimental data at 852 nm (circles) and calculated values (line) based on a 1:1 binding isotherm assuming that the six porphyrin units of **c-P6** act identically and independently.

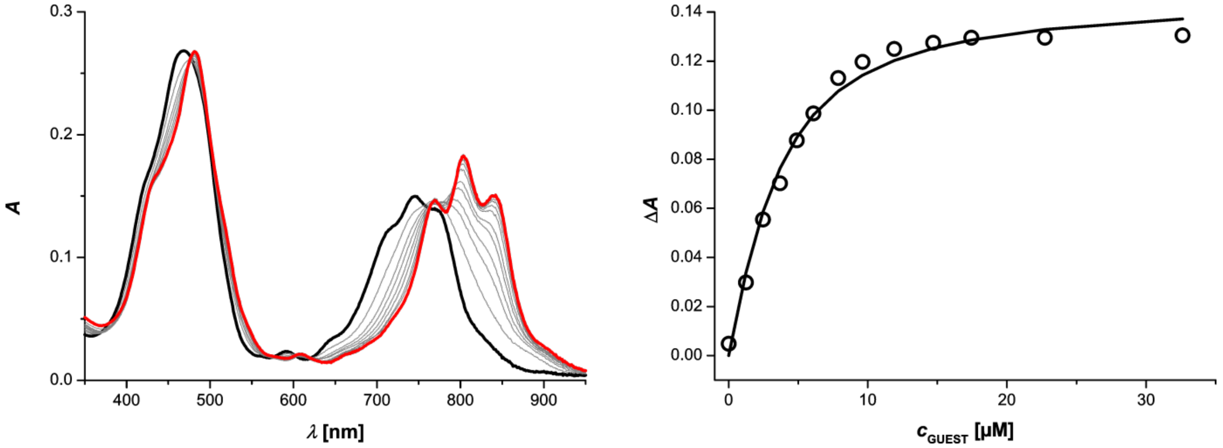

Figure S17. UV-vis-NIR titration ($CHCl_3$, 298 K) of a 1:1 mixture of **C** and **pA2** to **c-P6**. UV-vis-NIR spectra are shown on the left with the spectrum of unbound **c-P6** in thick black line and the end spectrum in red thick line. On the right are shown the experimental data at 843 nm (circles) and calculated values (line) based on a 1:1 binding isotherm assuming that the six porphyrin units of **c-P6** act identically and independently.

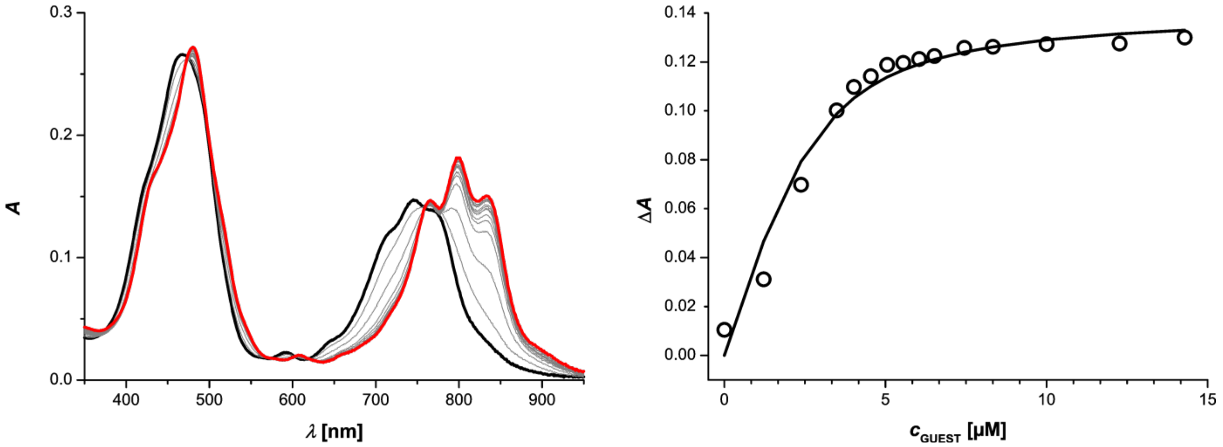

Figure S18. UV-vis-NIR titration ($CHCl_3$, 298 K) of a 1:1 mixture of **mB3** and **mA1** to **c-P6**. UV-vis-NIR spectra are shown on the left with the spectrum of unbound **c-P6** in thick black line and the end spectrum in red thick line. On the right are shown the experimental data at 835 nm (circles) and calculated values (line) based on a 1:1 binding isotherm assuming that the six porphyrin units of **c-P6** act identically and independently.

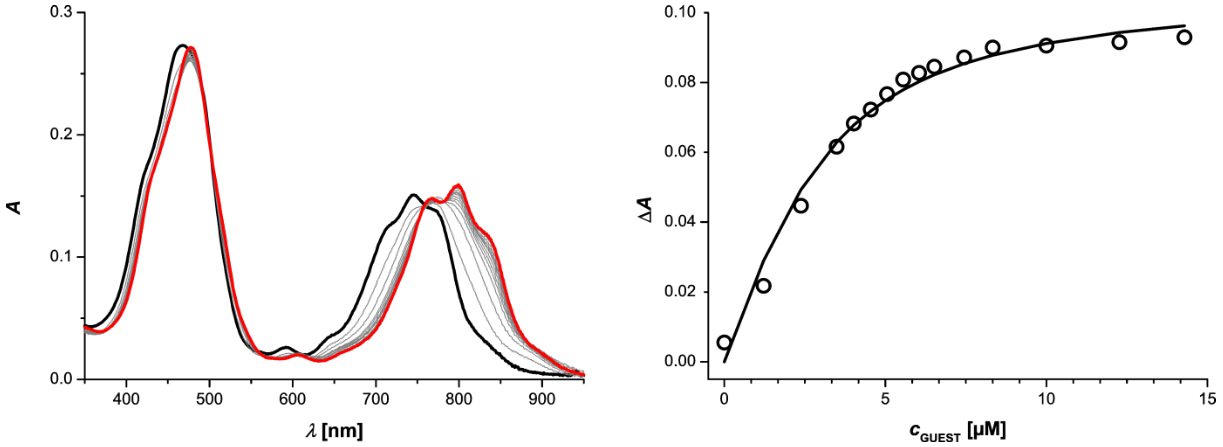

Figure S19. UV-vis-NIR titration ($CHCl_3$, 298 K) of a 1:1 mixture of **mB3** and **mA2** to **c-P6**. UV-vis-NIR spectra are shown on the left with the spectrum of unbound **c-P6** in thick black line and the end spectrum in red thick line. On the right are shown the experimental data at 838 nm (circles) and calculated values (line) based on a 1:1 binding isotherm assuming that the six porphyrin units of **c-P6** act identically and independently.

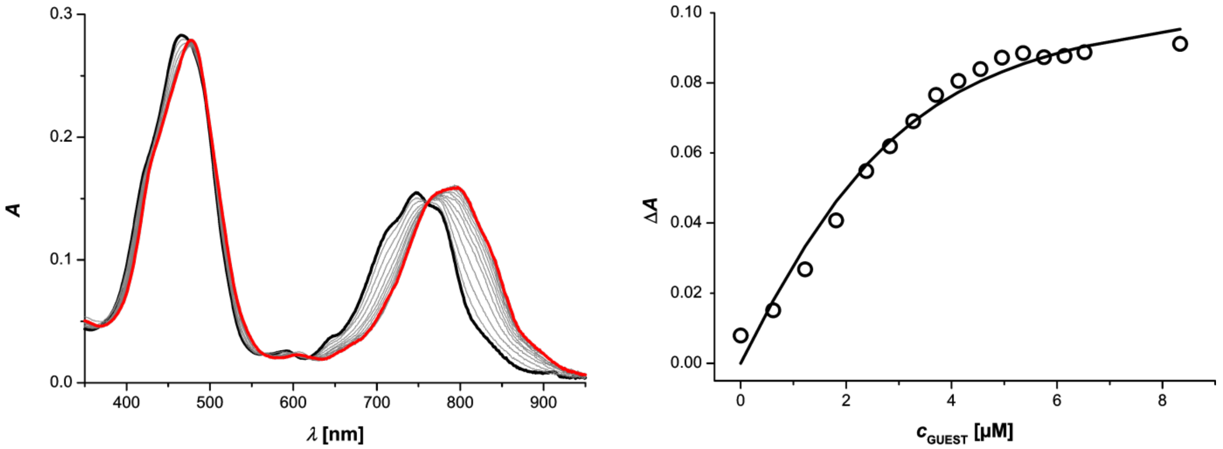

Figure S20. UV-vis-NIR titration ($CHCl_3$, 298 K) of a 1:1 mixture of **mB3** and **pA1** to **c-P6**. UV-vis-NIR spectra are shown on the left with the spectrum of unbound **c-P6** in thick black line and the end spectrum in red thick line. On the right are shown the experimental data at 831 nm (circles) and calculated values (line) based on a 1:1 binding isotherm assuming that the six porphyrin units of **c-P6** act identically and independently.

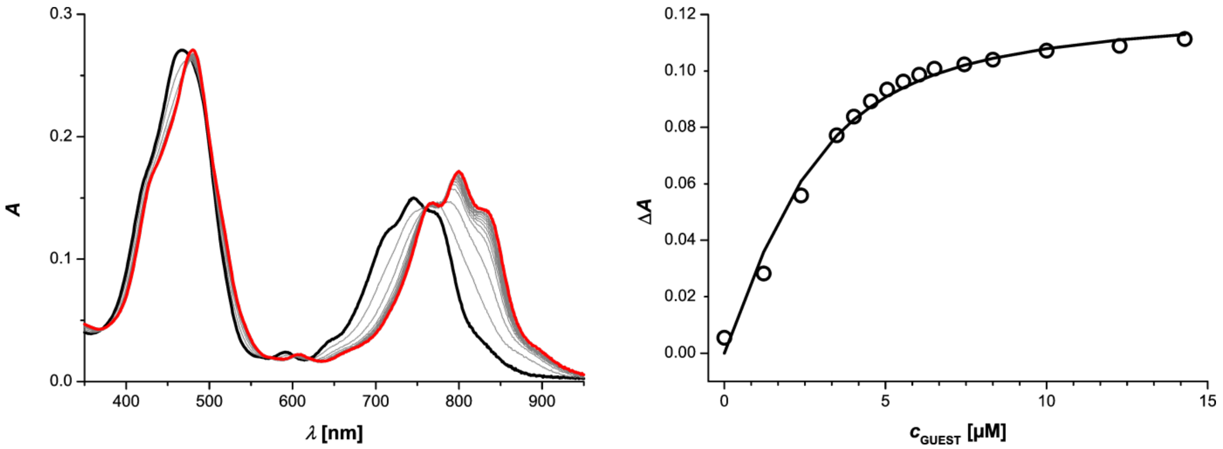

Figure S21. UV-vis-NIR titration ($CHCl_3$, 298 K) of a 1:1 mixture of **mB3** and **pA2** to **c-P6**. UV-vis-NIR spectra are shown on the left with the spectrum of unbound **c-P6** in thick black line and the end spectrum in red thick line. On the right are shown the experimental data at 842 nm (circles) and calculated values (line) based on a 1:1 binding isotherm assuming that the six porphyrin units of **c-P6** act identically and independently.

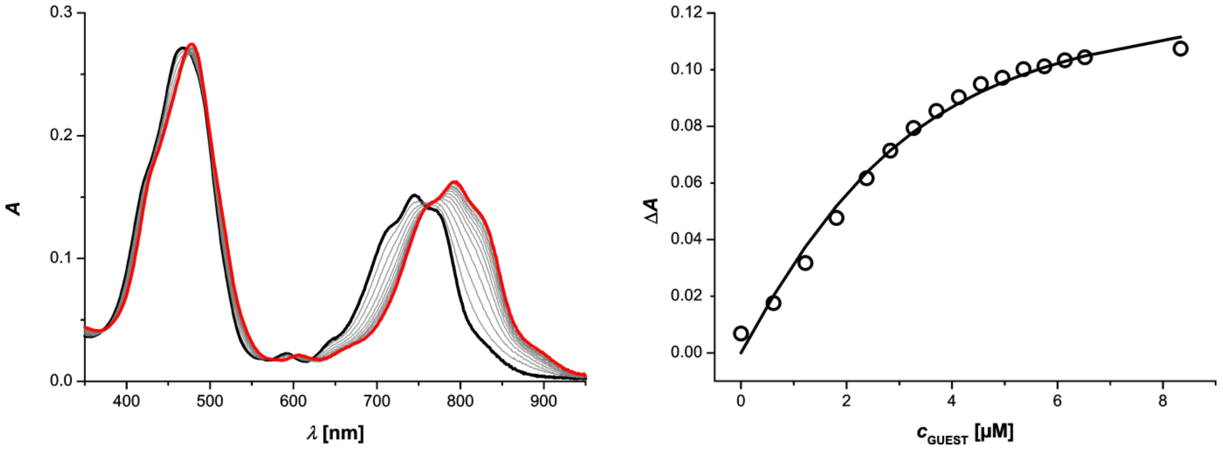

Figure S22. UV-vis-NIR titration ($CHCl_3$, 298 K) of a 1:1 mixture of **pB3** and **mA1** to **c-P6**. UV-vis-NIR spectra are shown on the left with the spectrum of unbound **c-P6** in thick black line and the end spectrum in red thick line. On the right are shown the experimental data at 831 nm (circles) and calculated values (line) based on a 1:1 binding isotherm assuming that the six porphyrin units of **c-P6** act identically and independently.

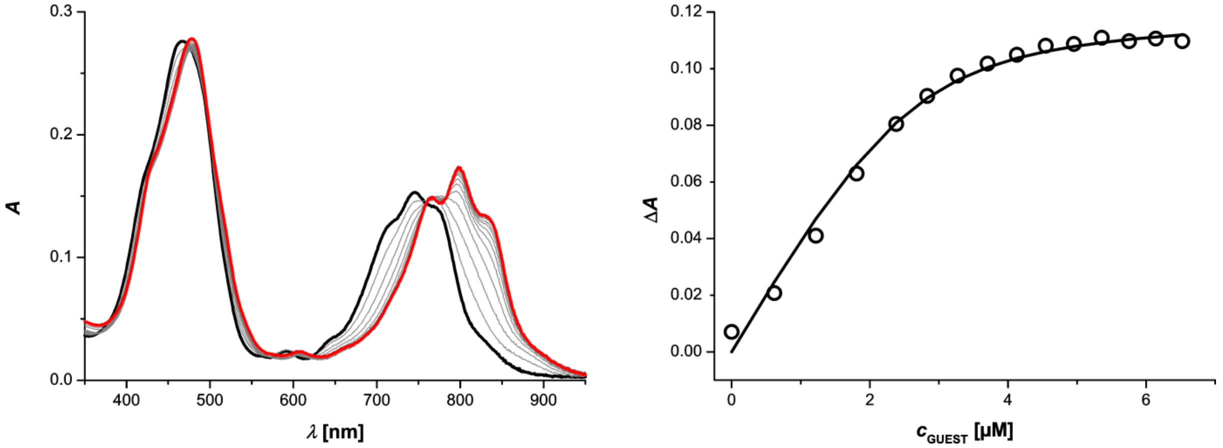

Figure S23. UV-vis-NIR titration ($CHCl_3$, 298 K) of a 1:1 mixture of **pB3** and **mA2** to **c-P6**. UV-vis-NIR spectra are shown on the left with the spectrum of unbound **c-P6** in thick black line and the end spectrum in red thick line. On the right are shown the experimental data at 837 nm (circles) and calculated values (line) based on a 1:1 binding isotherm assuming that the six porphyrin units of **c-P6** act identically and independently.

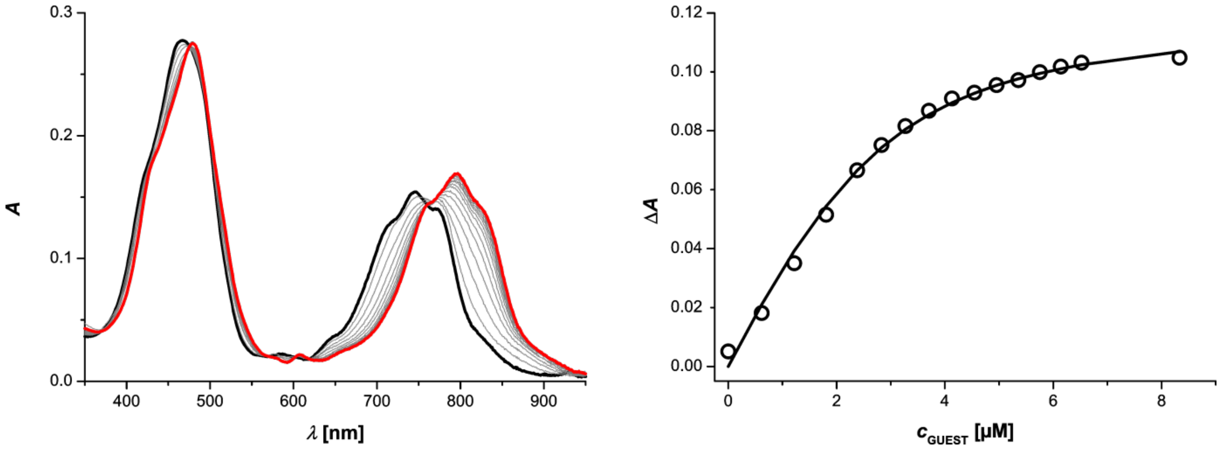

Figure S24. UV-vis-NIR titration ($CHCl_3$, 298 K) of a 1:1 mixture of **pB3** and **pA1** to **c-P6**. UV-vis-NIR spectra are shown on the left with the spectrum of unbound **c-P6** in thick black line and the end spectrum in red thick line. On the right are shown the experimental data at 834 nm (circles) and calculated values (line) based on a 1:1 binding isotherm assuming that the six porphyrin units of **c-P6** act identically and independently.

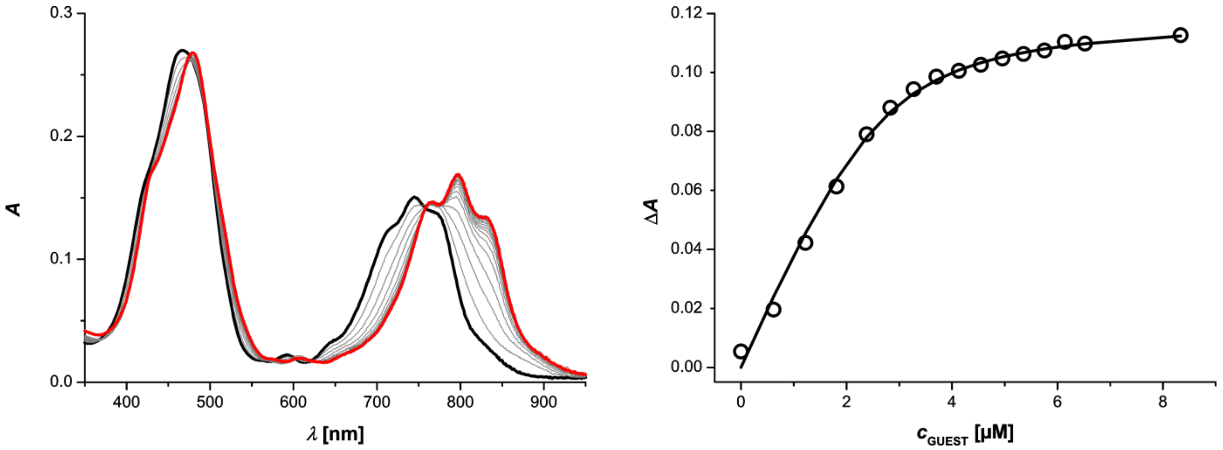

Figure S25. UV-vis-NIR titration ($CHCl_3$, 298 K) of a 1:1 mixture of **pB3** and **pA2** to **c-P6**. UV-vis-NIR spectra are shown on the left with the spectrum of unbound **c-P6** in thick black line and the end spectrum in red thick line. On the right are shown the experimental data at 835 nm (circles) and calculated values (line) based on a 1:1 binding isotherm assuming that the six porphyrin units of **c-P6** act identically and independently.

S3.4 Three component titrations fitted with an All-or-Nothing 1:3:3 isotherm

The titrations were analysed via non-linear regression using a global analysis multiple regression to model the entire spectrum simultaneously (ReactLab EQUILIBRIA software by Jplus Consulting). This allowed us to determine both the binding constants directly (expressed as log(K_f)) and the spectra of the species involved in the equilibrium processes (if not already known). The absorption spectra in the window 600-950 nm was used. The titrations were fit to a model where all ligands bind in one step (all-or-nothing) and one model where the ligands bind stepwise in pairs (stepwise). Graphical illustrations of the fits to both models at multiple wavelengths are shown below.

mB3+mA1

	Reactants	Reaction Type	Products	Label	Parameters logK/β	±	Fit þ
	M+3A+3B	=	MA3B3		38.29	0.011	TRUE

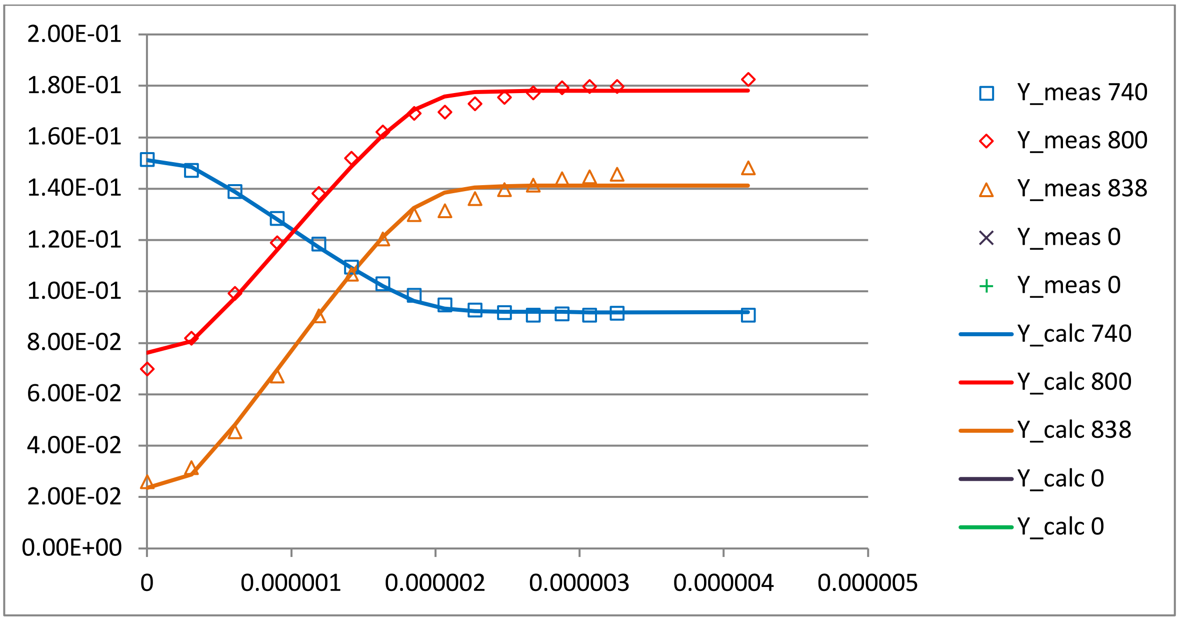

mB3+mA2

Reactants	Reaction Type	Products	Label	Parameters logK/β	±	Fit þ
M+3A+3B	=	MA3B3		37.78	0.027	TRUE

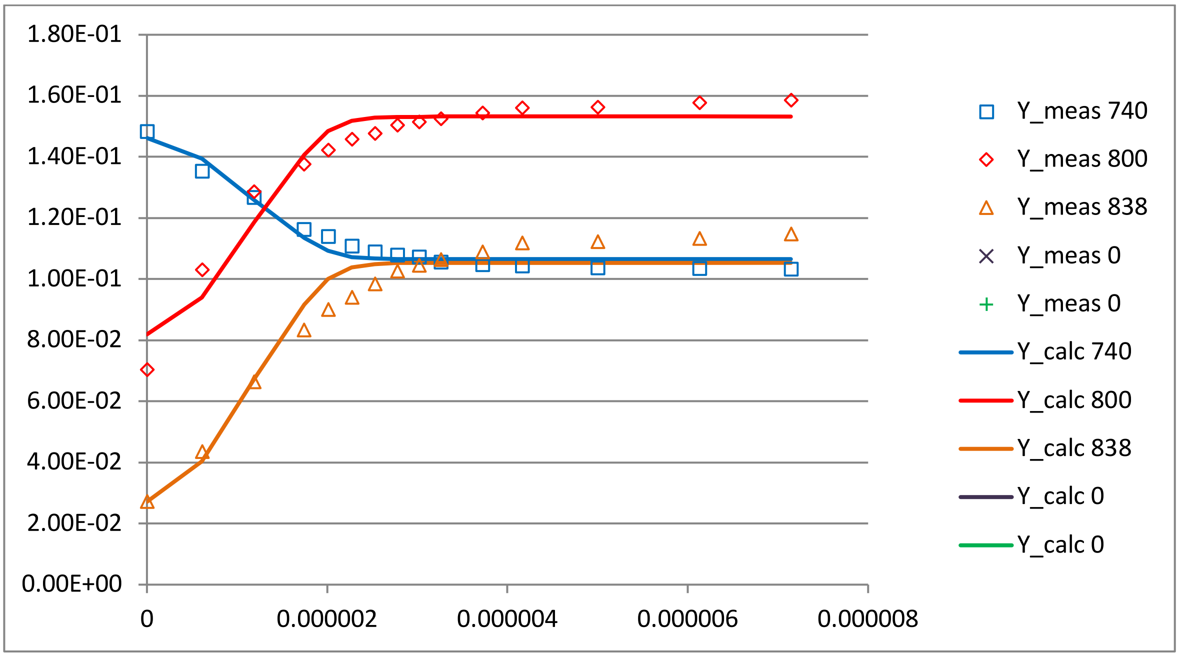

mB3+pA1

Reactants	Reaction Type	Products	Label	Parameters logK/β	±	Fit þ
M+3A+3B	=	MA3B3		38.01	0.016	TRUE

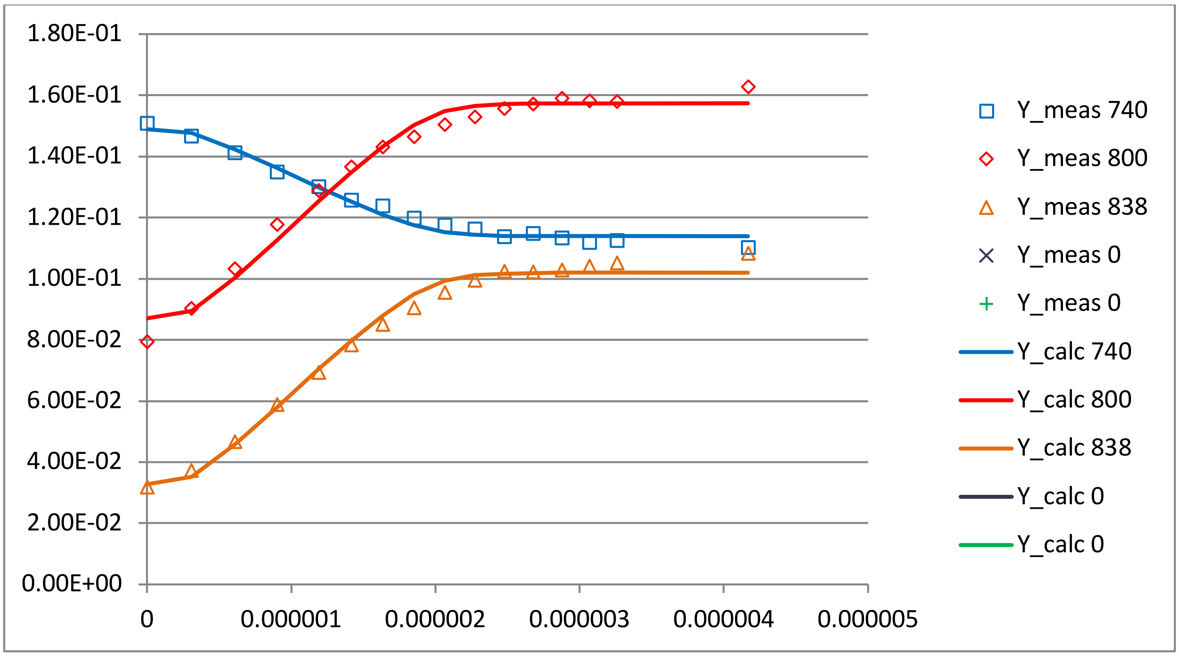

mB3+pA2

Reactants	Reaction Type	Products	Label	Parameters logK/β	±	Fit þ
M+3A+3B	=	MA3B3		38.48	0.031	TRUE

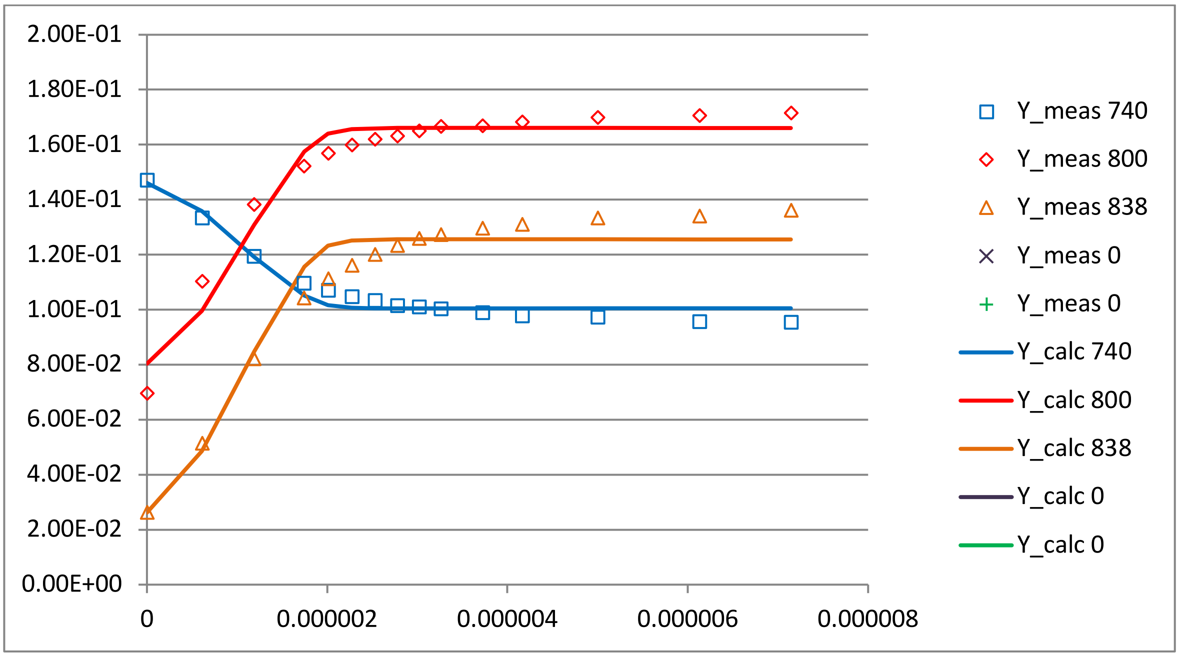

pB3+mA1

Reactants	Reaction Type	Products	Label	Parameters logK/β	±	Fit þ
M+3A+3B	=	MA3B3		38.64	0.024	TRUE

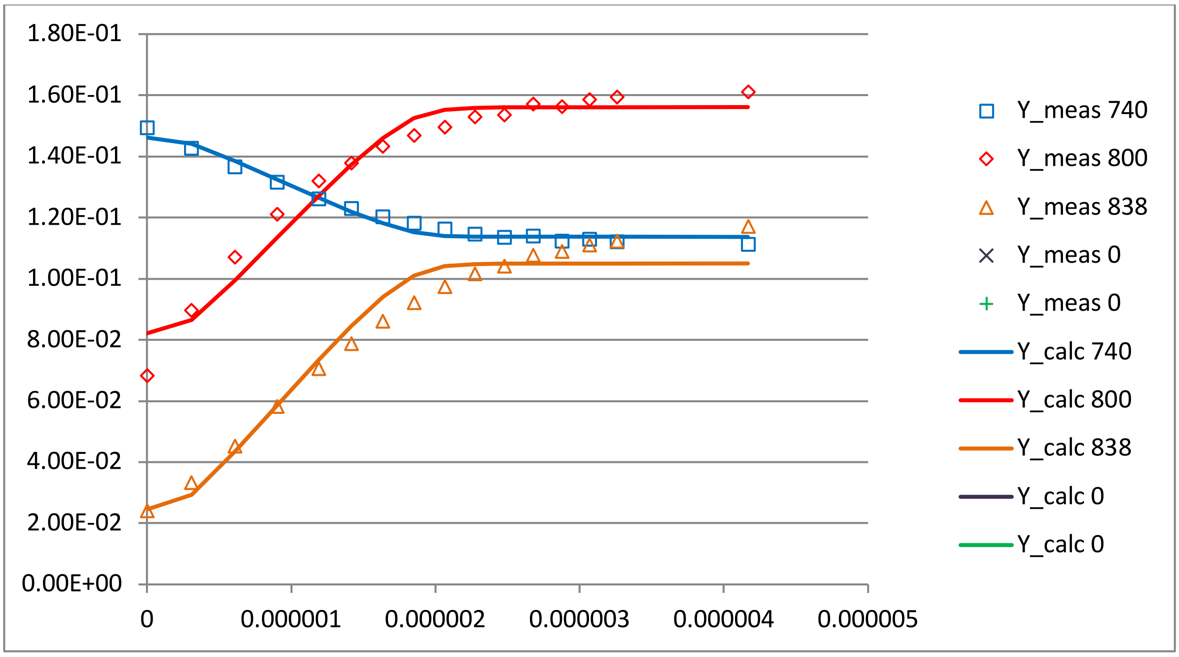

pB3+mA2

	Reactants	Reaction Type	Products	Label	Parameters logK/β	±	Fit þ
	M+3A+3B	=	MA3B3		42.09	0.063	TRUE

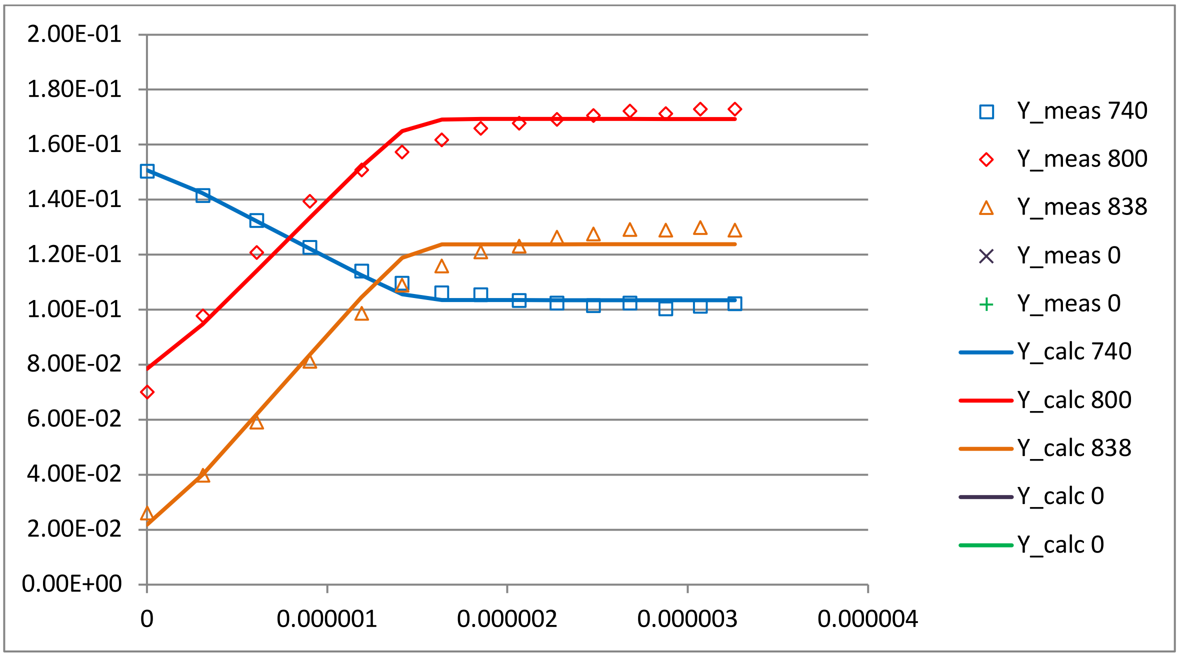

pB3+pA1

	Reactants	Reaction Type	Products	Label	Parameters logK/β	±	Fit þ
	M+3A+3B	=	MA3B3		39.14	0.026	TRUE

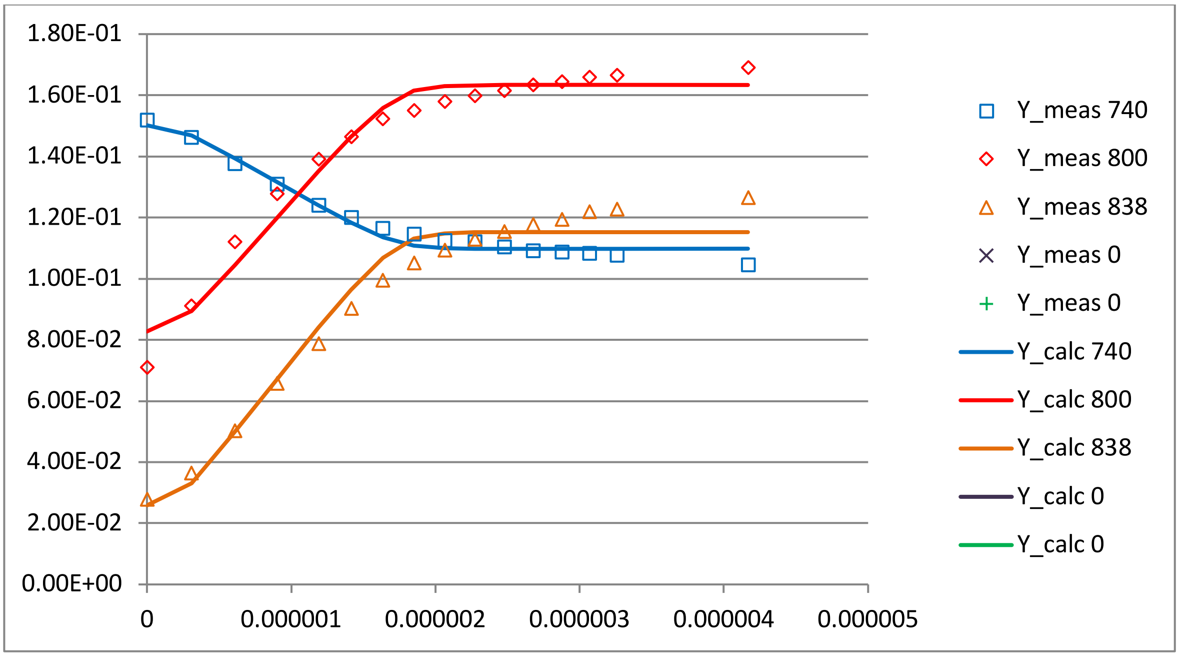

pB3+pA2

	Reactants	Reaction Type	Products	Label	Parameters logK/β	±	Fit þ
	M+3A+3B	=	MA3B3		41.84	0.060	TRUE

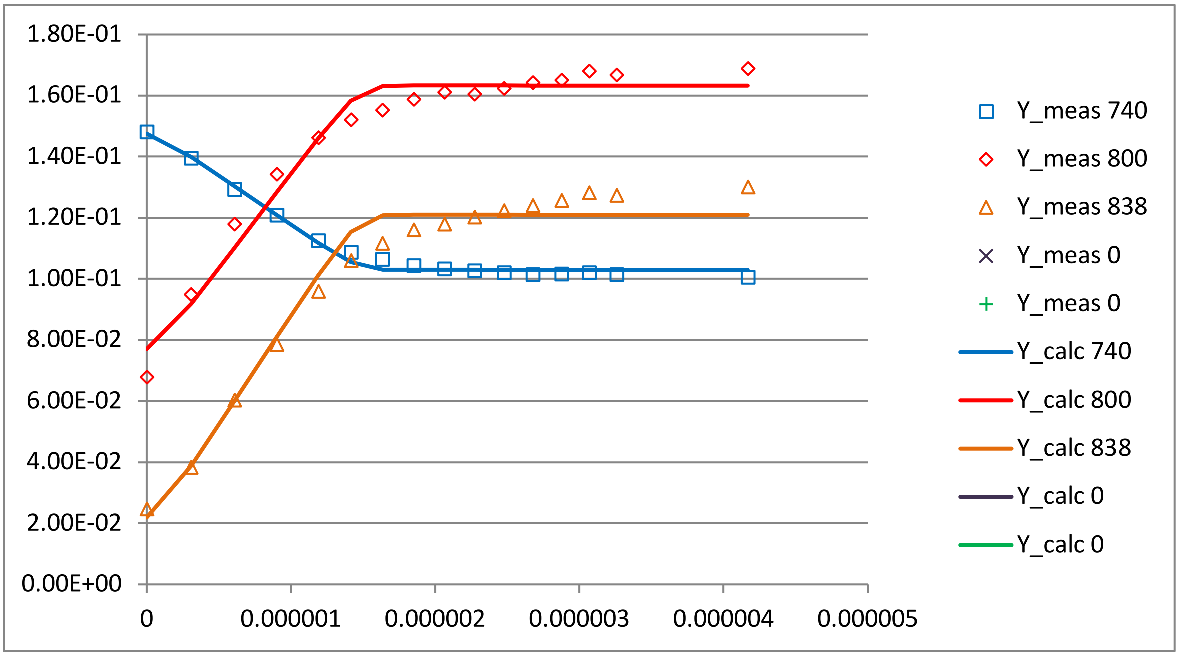

S3.5 Three component titrations fitted with a Stepwise 1:3:3 isotherm

ReactLab EQUILIBRIA software was also used to fit the titration data to a model where the ligands bind stepwise in pairs as indicated below (stepwise). Graphical illustrations of the fits at multiple wavelengths are shown below.

mB3+mA1

Reactants	Reaction Type	Products	Label	Parameters logK/β	±	Fit þ
M+A+B	=	MAB		13.55	0.034	TRUE
MAB+A+B	=	MA2B2		12.56	0.003	TRUE
MA2B2+A+B	=	MA3B3		11.16	0.053	TRUE

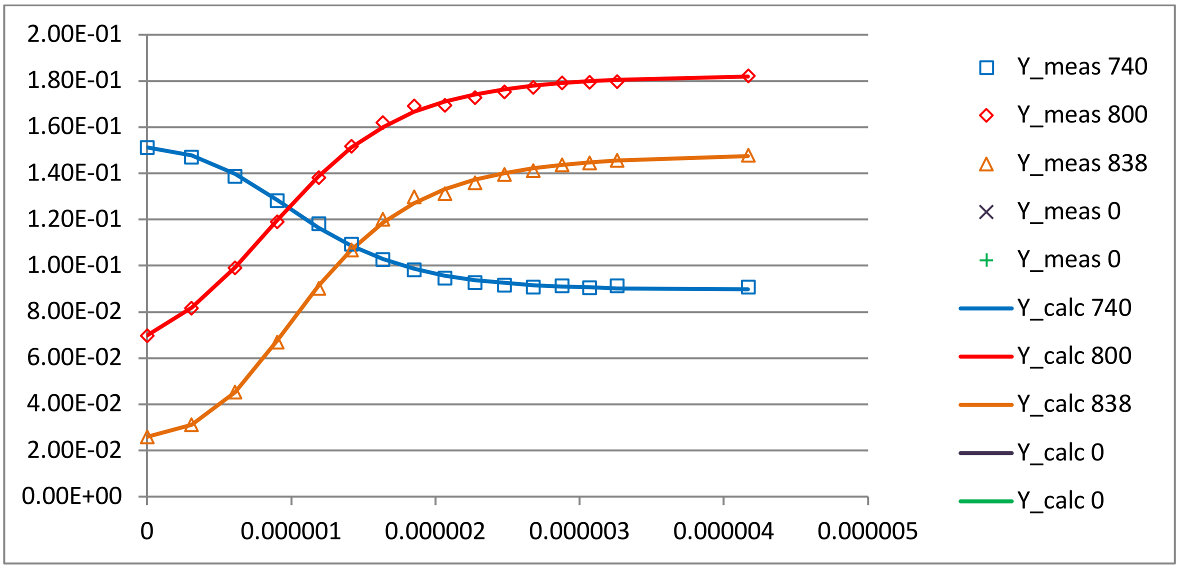

mB3+mA2

Reactants	Reaction Type	Products	Label	Parameters logK/β	±	Fit þ
M+A+B	=	MAB		12.88	0.019	TRUE
MAB+A+B	=	MA2B2		11.96	0.016	TRUE
MA2B2+A+B	=	MA3B3		11.64	0.037	TRUE

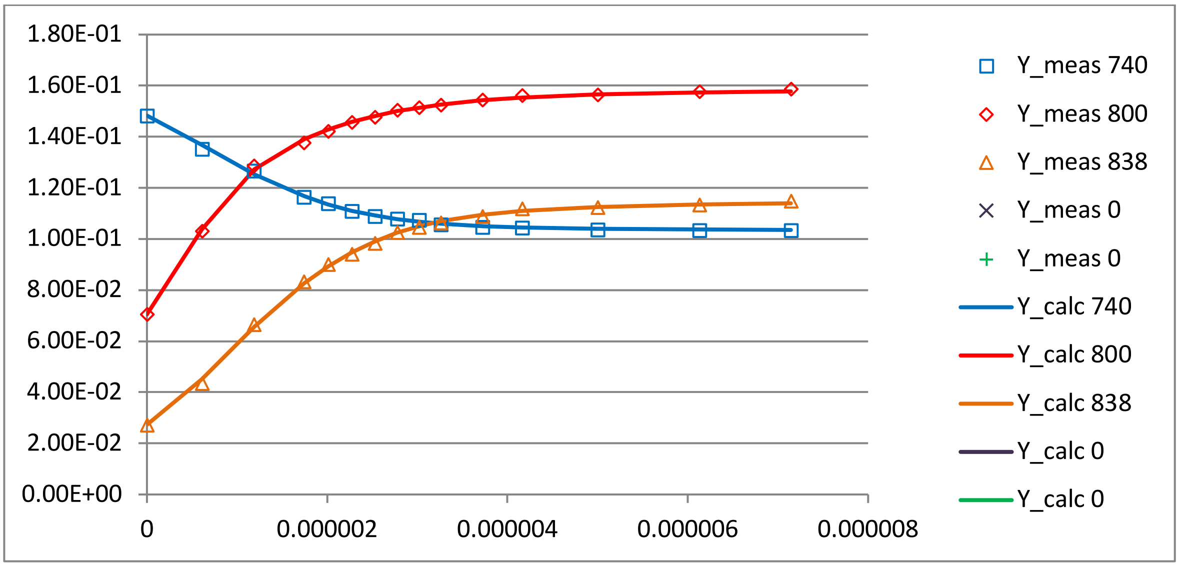

mB3+pA1

Reactants	Reaction Type	Products	Label	Parameters logK/β	±	Fit þ
M+A+B	=	MAB		13.05	0.040	TRUE
MAB+A+B	=	MA2B2		12.43	0.014	TRUE
MA2B2+A+B	=	MA3B3		11.93	0.022	TRUE

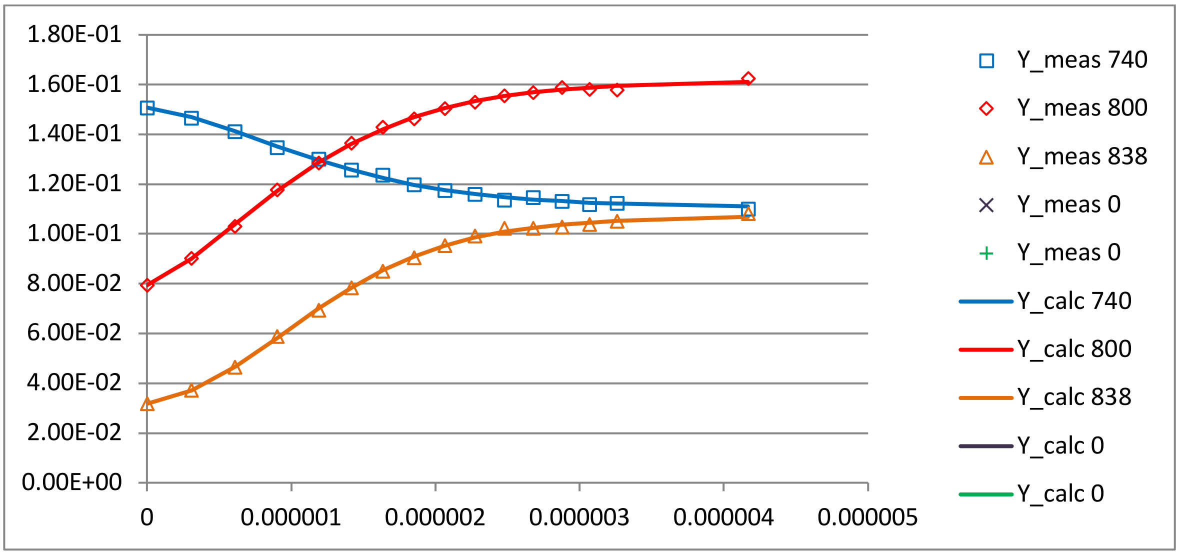

mB3+pA2

Reactants	Reaction Type	Products	Label	Parameters logK/β	±	Fit þ
M+A+B	=	MAB		13.12	0.013	TRUE
MAB+A+B	=	MA2B2		12.32	0.007	TRUE
MA2B2+A+B	=	MA3B3		11.38	0.009	TRUE

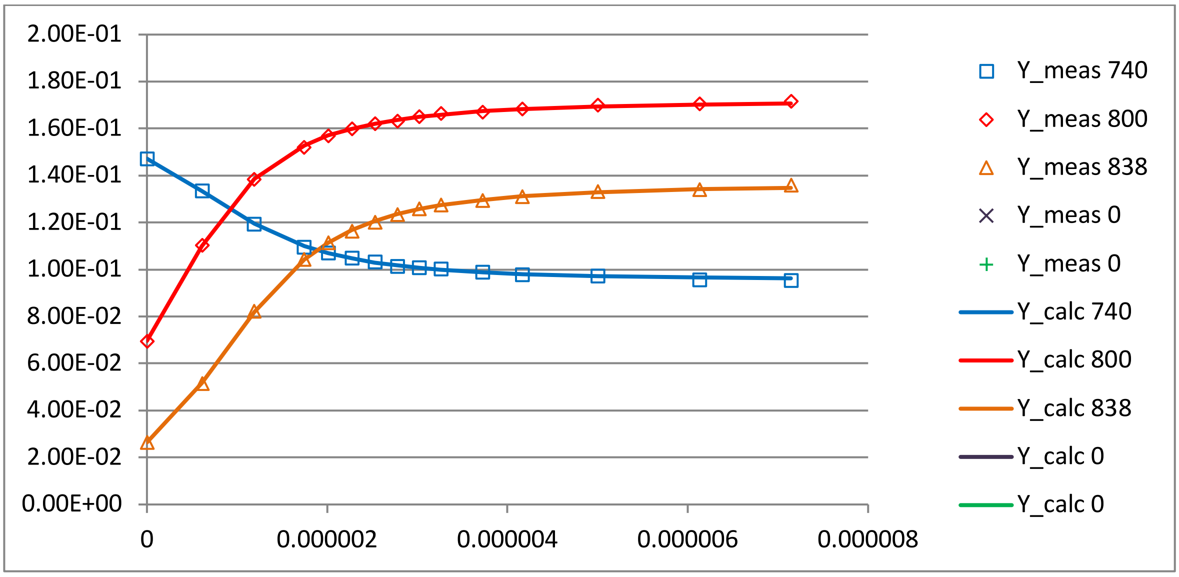

pB3+mA1

Reactants	Reaction Type	Products	Label	Parameters logK/β	±	Fit þ
M+A+B	=	MAB		13.49	0.022	TRUE
MAB+A+B	=	MA2B2		12.52	0.012	TRUE
MA2B2+A+B	=	MA3B3		12.07	0.012	TRUE

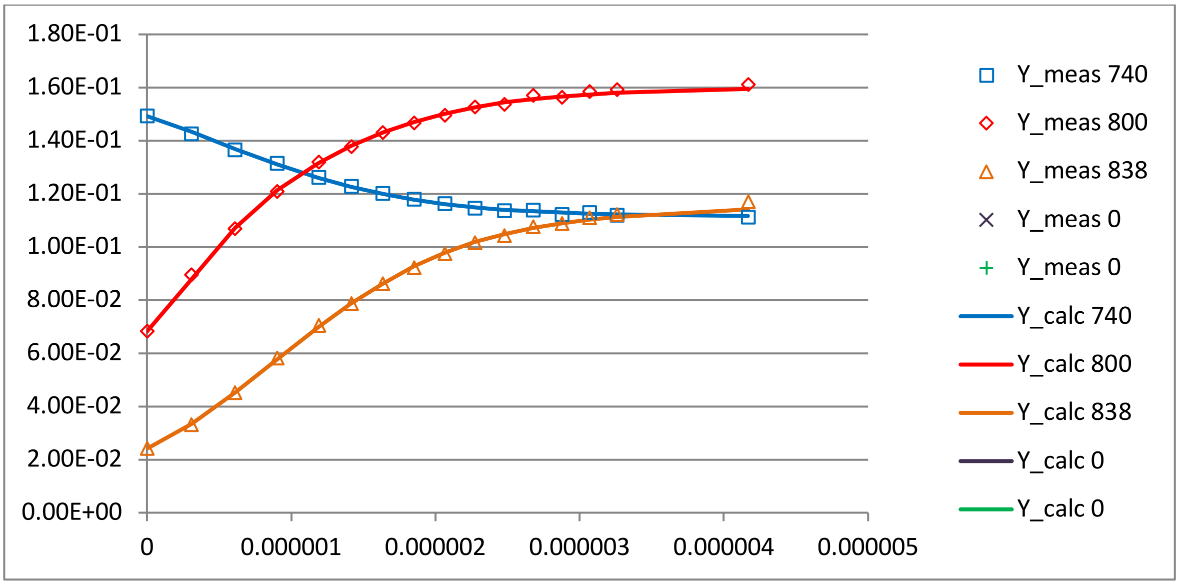

pB3+mA2

Reactants	Reaction Type	Products	Label	Parameters logK/β	±	Fit þ
M+A+B	=	MAB		13.67	0.021	TRUE
MAB+A+B	=	MA2B2		12.67	0.008	TRUE
MA2B2+A+B	=	MA3B3		11.87	0.030	TRUE

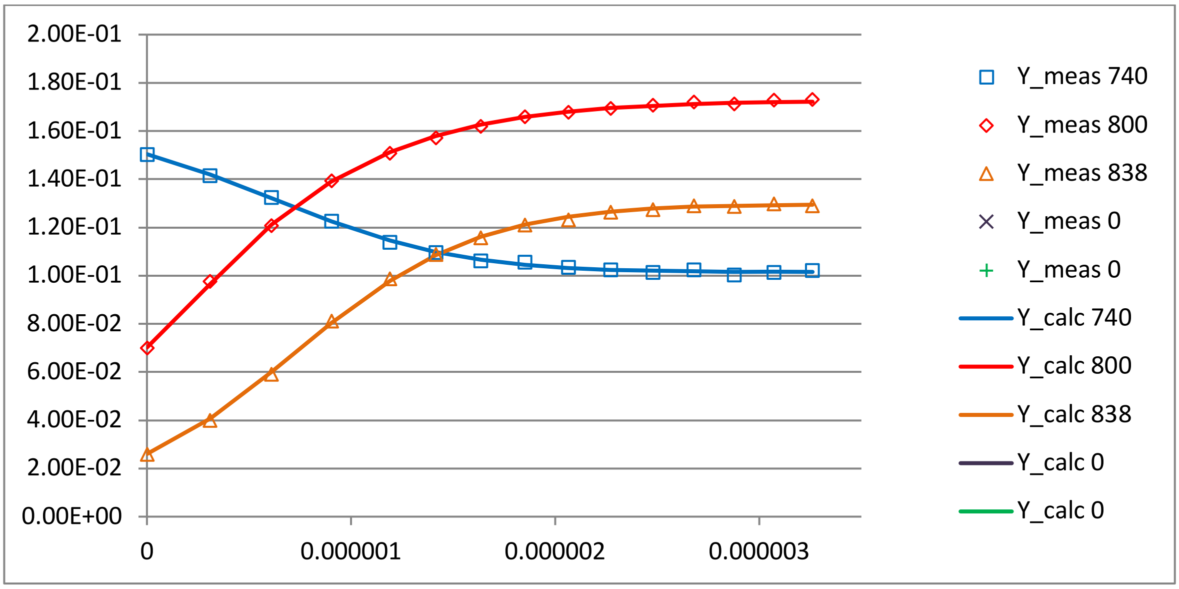

pB3+pA1

Reactants	Reaction Type	Products	Label	Parameters logK/β	±	Fit þ
M+A+B	=	MAB		13.32	0.036	TRUE
MAB+A+B	=	MA2B2		12.93	0.019	TRUE
MA2B2+A+B	=	MA3B3		12.15	0.012	TRUE

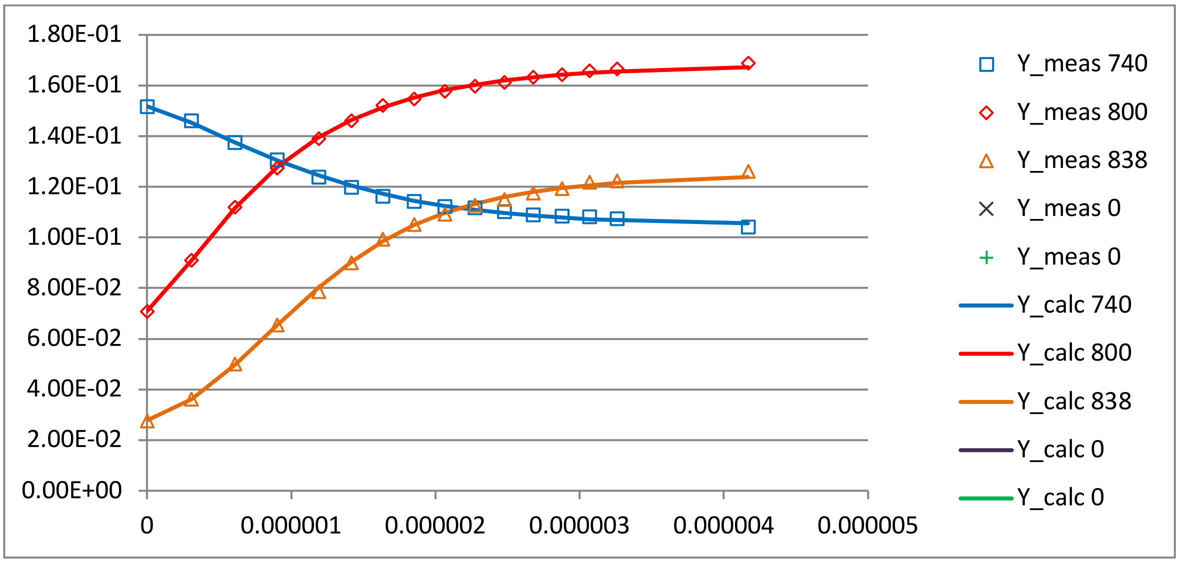

pB3+pA2

Reactants	Reaction Type	Products	Label	Parameters logK/β	±	Fit þ
M+A+B	=	MAB		13.98	0.030	TRUE
MAB+A+B	=	MA2B2		12.95	0.010	TRUE
MA2B2+A+B	=	MA3B3		11.75	0.016	TRUE

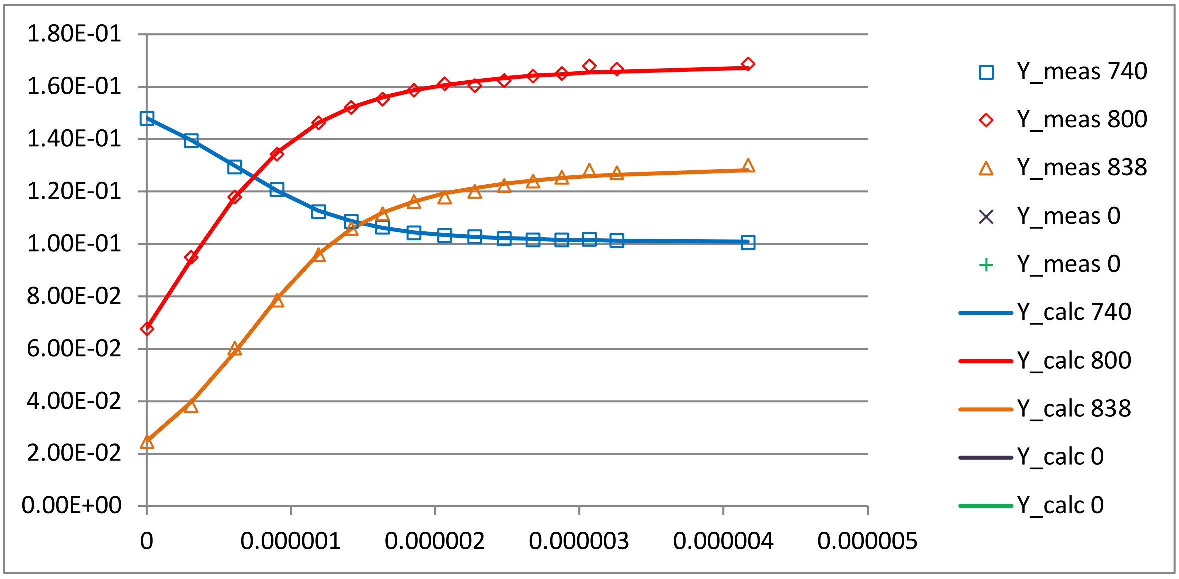

S3.6 Statistical Analysis of Quality of Fit to Different 1:3:3 Models

The statistical significance of the difference in the quality of the fit to the two different 1:3:3 models was tested using two statistical analyses: the extra sum-of-squares F-test and Akaike's information criterion.[3] The F-test compares the difference in sum-of-squares (SSQ) between a null-hypothesis and an alternative hypothesis with respect to the number of data points and the number of variable parameters used to fit the model. The sum-of-squares is calculated by taking all the deviations of the fit from the data points at each wavelength (residuals), squaring them, and adding them all together. The simpler model is used as the null-hypothesis and can then only be rejected if the alternative hypothesis with more variable parameters gives a significant improvement. The two models have to be nested, meaning that the null hypothesis is a special case of the alternative hypothesis. In this analysis, the number of data points minus the number of fitted parameters is called degrees of freedom (DoF). The value of F is calculated as the relative difference in SSQ divided by the relative difference in DoF using equation S1.

$$F = \frac{\mathrm{SSQ_{null}} - \mathrm{SSQ_{alternative}}}{\mathrm{SSQ_{alternative}}} / \frac{\mathrm{DoF_{null}} - \mathrm{DoF_{alternative}}}{\mathrm{DoF_{alternative}}} \qquad \text{(S1)}$$

The closer the ratio F is to 1, the more likely it is that the null hypothesis is correct and the improvement of the SSQ value in the alternative hypothesis is simply due to the higher flexibility of the fit. To interpret the significance of the F-test, its p-value has to be calculated.

Akaike's information criterion (AIC) is an alternative approach to comparing models which does not require the models to be nested and does not rely on p-values or the concept of statistical significance. The logic is not one of hypothesis testing, so you do not state a null hypothesis. Rather, the method lets you determine which model is more likely to be correct and quantify how much more likely. For each model, an AICc score is calculated using equation S2.

$$AIC_c = \mathrm{N} \cdot \ln\left(\frac{SSQ}{N}\right) + 2K + \frac{2K(K+1)}{N-K-1} \qquad \text{(S2)}$$

Where N is the number of data points and K is the number of variable parameters. The model which has the lowest AICc score is more likely to be correct. How much more likely it is to be correct depends on the absolute difference and can be calculated as the evidence ratio (equation S3).

$$\text{evidence ratio} = \frac{1}{\mathrm{e}^{-0.5 \cdot \Delta \mathrm{AIC_c}}} \qquad \text{(S3)}$$

The evidence ratio is the probability that the model with the lower AICc value is correct divided by the probability that the model with the higher AICc value is correct.

The number of data points is the total number of recorded spectra and the number of fitted parameters is the sum of the number of fitted spectra of species in solution and the number of fitted binding constants. For the all-or-nothing model there is one binding constant and two fitted spectra (***c*-P6** and ***c*-P6·A_{mB3}**), so the number of variable parameters is 3. For the stepwise model, there are three binding constants and four fitted spectra (***c*-P6**, ***c*-P6·AB**, ***c*-P6·A_2B_2** and ***c*-P6·A_{mB3}**), so the number of variable parameters is 7.

For the F-test analysis, the all-or-nothing model was used as the null hypothesis and the stepwise model was used as the alternative hypothesis. The p-values were calculated using an automatic calculator on the website https://www.socscistatistics.com/pvalues/fdistribution.aspx.

The details are provided below. In all cases, the p-value is less than 0.0015 and the evidence ratio is greater than 80 indicating that the stepwise model is most appropriate and that the increase in goodness of fit cannot be explained by the increase in parameters.

mB3+mA1	F-test			Akaike's Criterion	Information
	SSQ	DoF	var. para.		AICc
**all-or-nothing**	1.76E-02	13	3		-101.01
**stepwise**	2.90E-03	9	7		-109.85
**difference**	1.47E-02	4			8.84
**relative difference**	5.06	0.44			
**N**	16.00				
**F**	11.40				
**p-value**	0.001426			**evidence ratio**	83

mB3+mA2	F-test			Akaike's Criterion	Information
	SSQ	DoF	var. para.		AICc
**all-or-nothing**	5.26E-02	12.00	3		-76.60
**stepwise**	2.54E-03	8.00	7		-100.27
**difference**	5.01E-02	4.00			23.66
**relative difference**	19.74	0.50			
**N**	15.00	12.00			
**F**	39.48				
**p-value**	0.000026			**evidence ratio**	137455

mB3+pA1	F-test			Akaike's Criterion	Information
	SSQ	DoF	var. para.		AICc
**all-or-nothing**	2.14E-02	13.00	3		-97.87
**stepwise**	2.84E-03	9.00	7		-110.21
**difference**	1.86E-02	4.00			12.34
**relative difference**	6.55	0.44			
**N**	16.00				
**F**	14.73				
**p-value**	0.00055			**evidence ratio**	477

mB3+pA2	F-test			Akaike's Criterion	Information
	SSQ	DoF	var. para.		AICc
**all-or-nothing**	5.89E-02	13	3		-74.91
**stepwise**	9.31E-04	9	7		-115.32
**difference**	5.80E-02	4			40.40
**relative difference**	62.31	0.44			
**N**	15.00				
**F**	124.62				
**p-value**	< 0.00001			**evidence ratio**	593209999

**pB3+mA1**	**F-test**			**Akaike's Information Criterion**	
	SSQ	DoF	var. para.		AICc
**all-or-nothing**	4.02E-02	13.00	3		-87.80
**stepwise**	1.51E-03	9.00	7		-120.29
**difference**	3.87E-02	4.00			32.50
**relative difference**	25.61	0.44			
**N**	16.00				
**F**	57.61				
**p-value**	< 0.00001			**evidence ratio**	11397107

**pB3+mA2**	**F-test**			**Akaike's Information Criterion**	
	SSQ	DoF	var. para.		AICc
**all-or-nothing**	3.46E-02	12.00	3		-82.92
**stepwise**	2.42E-03	8.00	7		-101.00
**difference**	3.21E-02	4.00			18.08
**relative difference**	13.30	0.50			
**N**	15.00				
**F**	26.59				
**p-value**	0.000113			**evidence ratio**	8442

**pB3+pA1**	**F-test**			**Akaike's Information Criterion**	
	SSQ	DoF	var. para.		AICc
**all-or-nothing**	4.06E-02	13.00	3		-87.61
**stepwise**	2.48E-03	9.00	7		-112.38
**difference**	3.82E-02	4.00			24.77
**relative difference**	15.41	0.44			
**N**	16.00				
**F**	34.67				
**p-value**	0.000018			**evidence ratio**	238747

**pB3+pA2**	**F-test**			**Akaike's Information Criterion**	
	SSQ	DoF	var. para.		AICc
**all-or-nothing**	3.86E-02	13.00	3		-88.42
**stepwise**	2.81E-03	9.00	7		-110.38
**difference**	3.58E-02	4.00			21.96
**relative difference**	12.77	0.44			
**N**	16.00				
**F**	28.74				
**p-value**	0.000039			**evidence ratio**	58757

S4: DOSY Spectrum

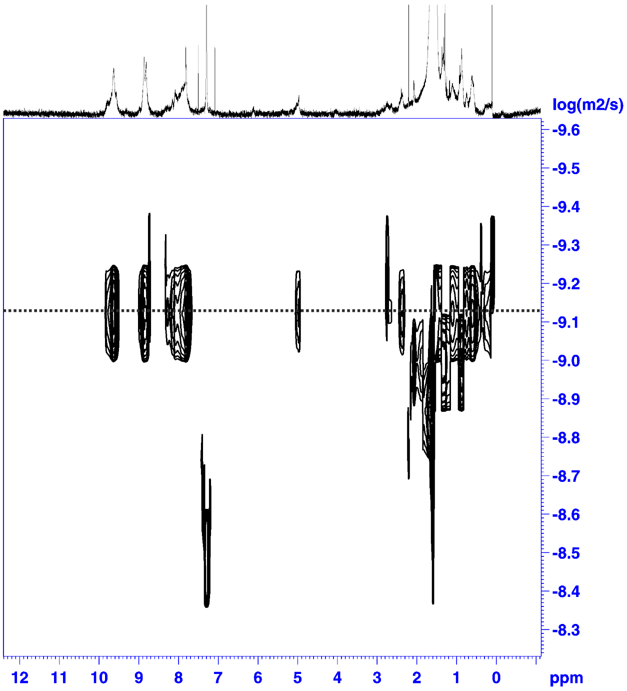

Figure S26. ^{1}H NMR DOSY spectrum (500 MHz, $CDCl_3$, 298 K) of a 1:3:3 mixture of ***c*-P6** (0.16 mM), **pB3** and **mA2** (0.48 mM).

S5: Molecular Modelling

The structure of **T** from the X-ray structure of **c-P6•T** was used as a template to determine the positions of the six pyridines when they are bound to the porphyrin nanoring. The side chain H-bonding groups required for rosette formation were then built onto the six pyridine fragments. A conformational search was carried out using the OPLS3 force field in MacroModel implemented in the Schrödinger Suite 2016-4. Calculations were carried out with no solvent and with no cut-off for non-covalent interactions, but the coordinates of the nitrogen atoms and carbon atoms in the position 4 were constrained, allowing the pyridine units to rotate around their axes but not to move (Figure S27). Figure S28 shows an example of the outcome: the lowest energy structure obtained for **mB3$_3$•mA2$_3$** is the H-bonded rosette.

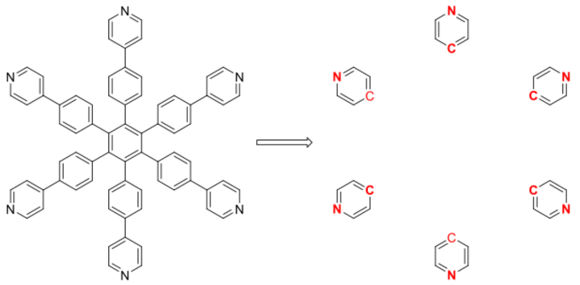

Figure S27. Using **T** as a framework for construction of rosette models. The coordinated of the atoms in red were constrained for the calculations.

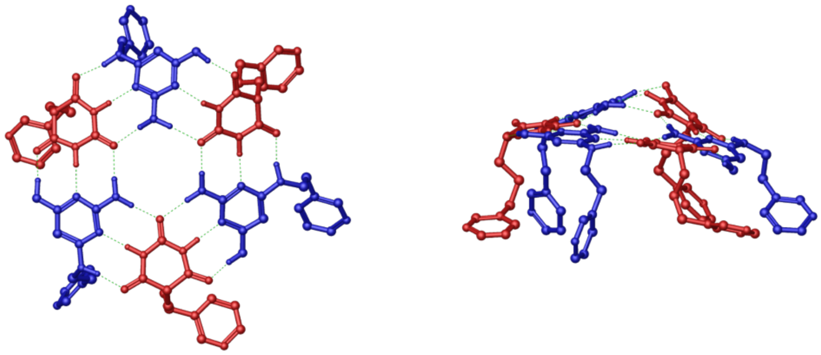

Figure S28. Top and side views of the lowest energy structure of **mB3$_3$•mA2$_3$** rosette system from a conformational search, where the coordinates of the nitrogen atoms and carbon atoms in the position 4 of pyridine units were constrained (the alkyl groups of both barbiturate-pyridines and pyrimidine-pyridines were replaced by hydrogen atoms in the calculations). Hydrogen atoms that do not contribute to H-bonding are not shown for clarity; colouring: **mB$_3$** in red, **mA$_2$** in blue, green dashed line is the H-bond.

The pyridine groups from the lowest energy structure obtained using molecular mechanics were superimposed on the positions of the pyridine groups in the X-ray crystal structure of **c-P6•T**, and the resulting structure was optimised using the semi-empirical PM6 method as implemented in Gaussian 09.[4] In this way, it was possible to find structures of the rosette motif bound inside the porphyrin nanoring for several ligand combinations, and the results are shown in Figure S29.

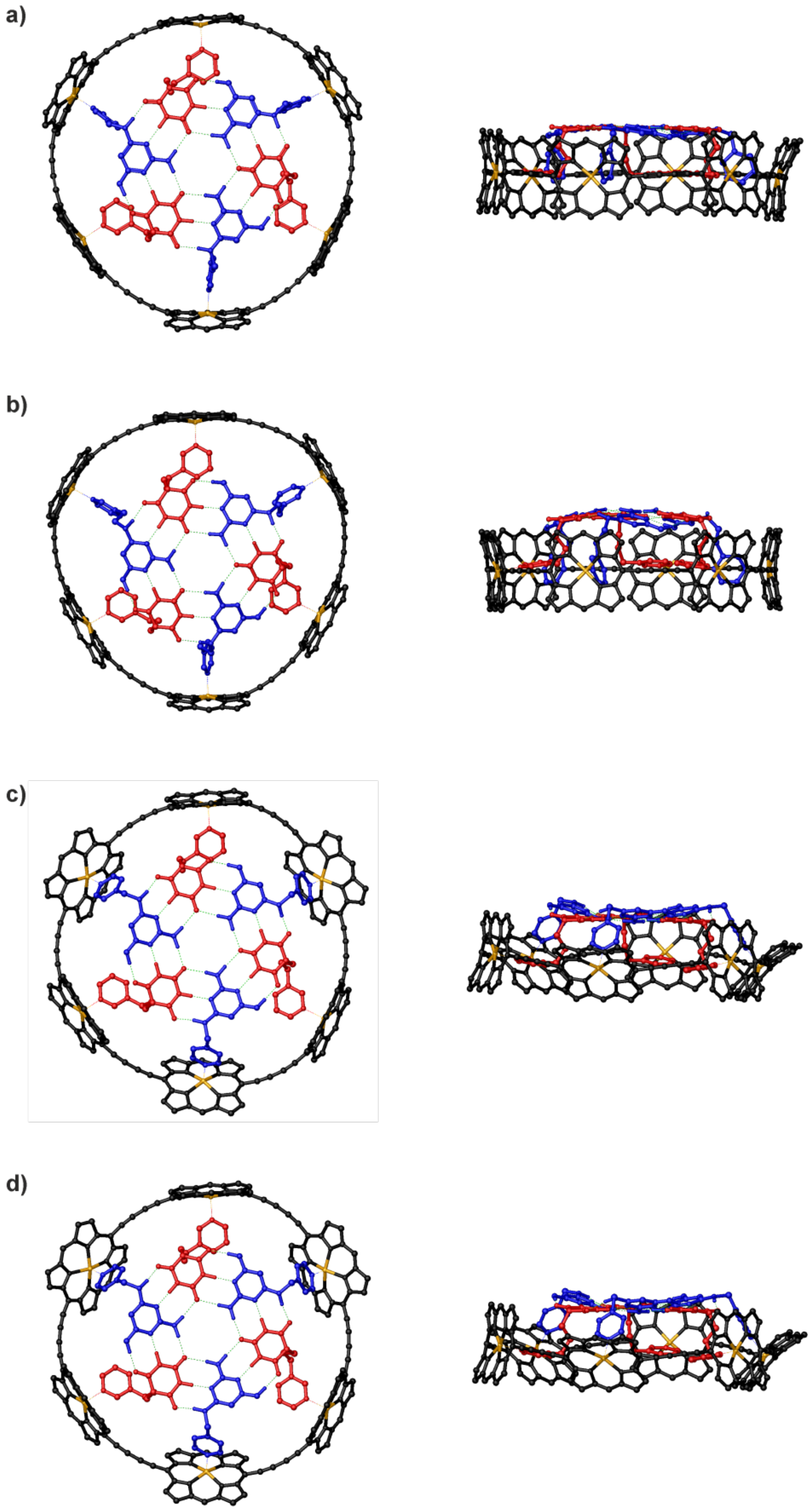

Figure S29. Top and side views of the PM6-optimised structures of H-bonded rosettes bound inside the porphyrin nanoring (a) **c-P6•mB3$_3$•mA1$_3$**, (b) **c-P6•mB3$_3$•mA2$_3$**, (c) **c-P6•mB3$_3$•pA1$_3$**, (d) **c-P6•mB3$_3$•pA2$_3$** (alkyl groups on barbiturate-pyridines and pyrimidine-pyridines and 3,5-bis(t-butyl)phenyl on **c-P6** were replaced by hydrogen atoms in the calculations). Hydrogen atoms that do not contribute to H-bonding are not shown for clarity. Colouring: **c-P6** in black with highlighted Zn atoms in yellow, barbiturate-pyridines in red, pyrimidine-pyridines in blue, green dashed line is the H-bond.

It was also possible to obtain a model of rosette-like structure of **c-P6•mB3$_6$** using the same approach (Figure S30).

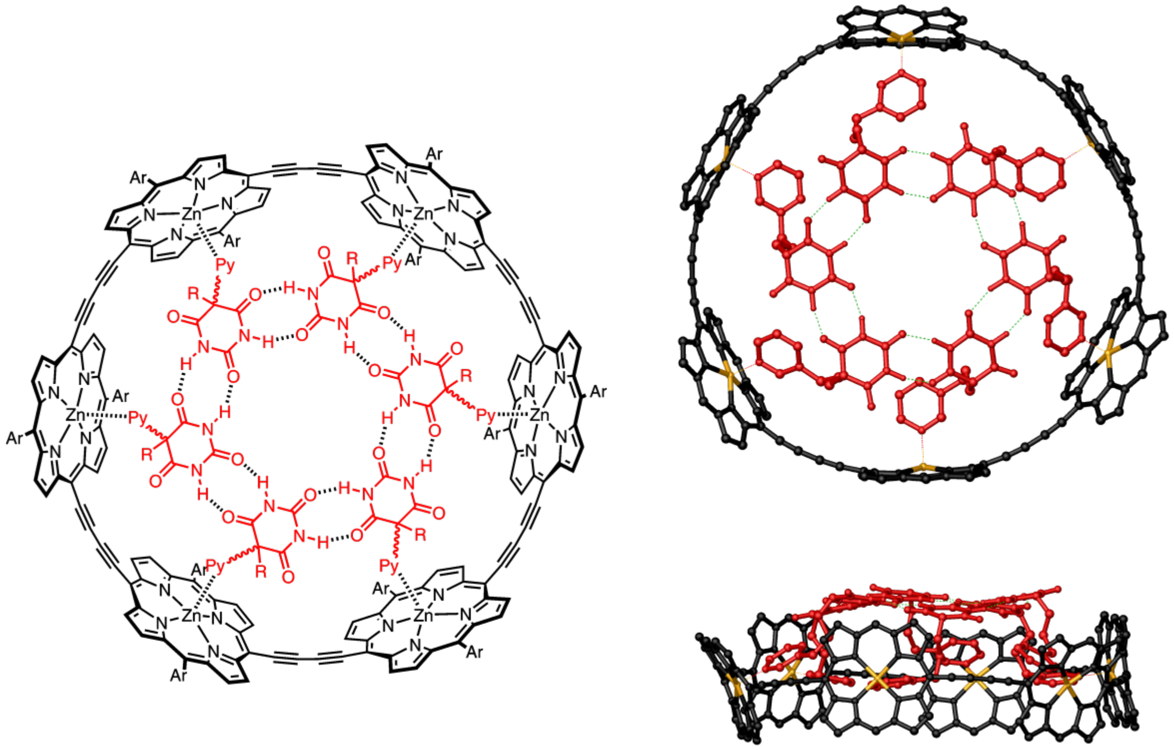

Figure S30. Top and side views of the PM6-optimised structure of **c-P6•mB3$_6$** (zinc centres in yellow and H-bonding interactions in green). Alkyl groups on **mB3** and the 3,5-bis(t-butyl)phenyl groups on **c-P6** were replaced by hydrogen atoms in the calculations, and hydrogen atoms that do not contribute to H-bonding are not shown for clarity.

S6: References

1) Motloch, P., Hunter, C. A. *Org. Biomol. Chem.* **2020**, *18*, 1602–1606.
2) (a) Hoffmann, M.; Kärnbratt, J.; Chang, M.-H.; Herz, L. M.; Albinsson, B.; Anderson, H. L. *Angew. Chem. Int. Ed.* **2008**, *47*, 4993-4996. (b) Sprafke, J. K.; Kondratuk, D. V.; Wykes, M.; Thompson, A. L.; Hoffmann, M.; Drevinskas, R.; Chen, W.-H.; Yong, C. K.; Kärnbratt, J.; Bullock, J. E.; Malfois, M.; Wasielewski, M. R.; Albinsson, B.; Herz, L. M.; Zigmantas, D.; Beljonne, D.; Anderson, H. L. *J. Am. Chem. Soc.* **2011**, *133*, 17262-17273.
3) Motulsky, H. J.; Christopoulos, A. *Fitting models to biological data using linear and nonlinear regression. A practical guide to curve fitting.* (Chapters 21-23). 2003, GraphPad Software Inc., San Diego CA, www.graphpad.com.
4) Frisch, M. J.; Trucks, G. W.; Schlegel, H. B.; Scuseria, G. E.; Robb, M. A.; Cheeseman, J. R.; Scalmani, G.; Barone, V.; Mennucci, B.; Petersson, G. A.; Nakatsuji, H.; Caricato, M.; Li, X.; Hratchian, H. P.; Izmaylov, A. F.; Bloino, J.; Zheng, G.; Sonnenberg, J. L.; Hada, M.; Ehara, M.; Toyota, K.; Fukuda, R.; Hasegawa, J.; Ishida, M.; Nakajima, T.; Honda, Y.; Kitao, O.; Nakai, H.; Vreven, T.; Montgomery Jr., J. A.; Peralta, J. E.; Ogliaro, F.; Bearpark, M. J.; Heyd, J.; Brothers, E. N.; Kudin, K. N.; Staroverov, V. N.; Kobayashi, R.; Normand, J.; Raghavachari, K.; Rendell, A. P.; Burant, J. C.; Iyengar, S. S.; Tomasi, J.; Cossi, M.; Rega, N.; Millam, N. J.; Klene, M.; Knox, J. E.; Cross, J. B.; Bakken, V.; Adamo, C.; Jaramillo, J.; Gomperts, R.; Stratmann, R. E.; Yazyev, O.; Austin, A. J.; Cammi, R.; Pomelli, C.; Ochterski, J. W.; Martin, R. L.; Morokuma, K.; Zakrzewski, V. G.; Voth, G. A.; Salvador, P.; Dannenberg, J. J.; Dapprich, S.; Daniels, A. D.; Farkas, Ö.; Foresman, J. B.; Ortiz, J. V.; Cioslowski, J.; Fox, D. J.; Gaussian, Inc.: Wallingford, CT, USA, 2009.
